# Functional divergence of two soybean cytosolic serine hydroxymethyltransferases in development and defense against soybean cyst nematode

**DOI:** 10.3389/fpls.2026.1942553

**Published:** 2026-08-31

**Authors:** Vinavi A. Gamage, Luckio F. Owuocha, Feng Lin, Zenglu Li, Lesa J. Beamer, Melissa G. Mitchum

**Affiliations:** 1 Department of Plant Pathology and Institute of Plant Breeding, Genetics and Genomics, University of Georgia, Athens, GA, United States; 2 Department of Biochemistry, University of Missouri, Columbia, MO, United States; 3 Division of Plant Science and Technology, University of Missouri, Columbia, MO, United States; 4 Department of Crop and Soil Sciences, and Institute of Plant Breeding, Genetics and Genomics, University of Georgia, Athens, GA, United States

**Keywords:** 1-C folate metabolism, *Glycine max* (L), *Heterodera glycines* Ichinohe, resistance, serine hydroxymethyltransferase (SHMT), soybean, soybean cyst nematode

## Abstract

Serine hydroxymethyltransferase (SHMT) is an enzyme essential for one-carbon metabolism. In higher plants, multiple *SHMT* genes code for isoforms that function in the cytosol, nucleus, mitochondria, and chloroplasts. The soybean genome contains two cytosolic *SHMT*s, *GmSHMT05* and *GmSHMT08*, sharing high sequence identity and similar expression throughout soybean development. In certain soybean genotypes, two amino acid substitutions negatively impact GmSHMT08’s ability to bind to tetrahydrofolate (THF), leading to a gain-of-function in resistance to the soybean cyst nematode (SCN). Whether this perturbation to the enzyme has other functional consequences for soybean growth and development remains unknown. Here, we investigated the roles of cytosolic *GmSHMTs* in soybean growth and development. We determined that the 3D structure and folate-binding affinity of GmSHMT05 are highly similar to the version of GmSHMT08 found in susceptible soybeans. We further measured phenotypic traits of two ethyl methanesulfonate-derived *Gmshmt08* mutant plants in an SCN-resistant soybean background. Aboveground soybean growth and development were similar, except the *Gmshmt08* mutant plants showed a significant increase in pods/plant in field phenotyping trials. Belowground analyses revealed a significant increase in lateral root and total root length in mutant plants, and CRISPR-Cas9 editing demonstrated an essential role of cytosolic *SHMTs* in root growth. Taken together, our results indicate that *GmSHMT05* sustains overall soybean growth and development in the absence of *GmSHMT08*; however, GmSHMT08’s gain-of-function in SCN resistance negatively influences pod and root growth, highlighting a potential trade-off between soybean defense and development that may impact yield when breeding with *GmSHMT08* to develop SCN-resistant varieties.

## Introduction

Serine hydroxymethyltransferases (SHMTs) (EC 2.1.2.1) are a class of α-type pyridoxal-5’-phosphate (PLP)-dependent enzymes that play a pivotal role in folate-dependent one-carbon (1C) metabolism. Folate-dependent 1C metabolism is fundamental to several plant cellular processes, including DNA biosynthesis, methylation, and photorespiration. SHMTs catalyze the reversible conversion of serine and tetrahydrofolate (THF) into glycine and methylene-5,10-tetrahydrofolate, a donor of 1C units. Methylene-5,10-THF is subsequently converted into 10-formyl-THF through 5,10-methenyl-THF, or into 5-methyl-THF. Methylene-5,10-THF is crucial for the synthesis of thymidylate and pantothenate, while 10-formyl-THF is essential for the biosynthesis of purines and formylmethionyl-tRNA, the latter being necessary for translation initiation in plastids and mitochondria. On the other hand, 5-methyl-THF serves exclusively in the synthesis of methionine, which in turn produces S-adenosylmethionine (AdoMet/SAM), a vital methyl donor required for a wide array of biological methylation reactions and plant defense compounds (Schirch, 1982; Chen et al., 1997; Ravanel et al., 1998; Mouillon et al., 1999; Hanson et al., 2000; Hanson and Roje, 2001).

In higher plants, multiple *SHMT* genes code for isoforms that function in the cytosol, nucleus, mitochondria, and chloroplasts (McClung et al., 2000; Lakhssassi et al., 2019; Liu et al., 2022a). Genome-wide analyses have identified *SHMT* genes from a number of different plant species, including rice (five members, Pan et al., 2024), *Arabidopsis* (seven members, Nogués et al., 2022), tomato (seven members, Liu et al., 2022a), soybean (14 members, Wu et al., 2016), alfalfa (14 members, Gao et al., 2024), and wheat (17 members, Liu et al., 2022b).

Overexpression and mutant studies, primarily in *Arabidopsis*, have begun to shed light on the essential function of SHMTs in plant growth and development. *A. thaliana* SHMT isoforms localize to the mitochondria (AtSHMT1, AtSHMT2), chloroplast (AtSHMT3), cytosol (AtSHMT4, AtSHMT5), and nucleus (AtSHMT6, AtSHMT7) (Nogués et al., 2022). In *Arabidopsis* and rice, the mitochondria-localized SHMT1 plays a crucial role in the photorespiratory pathway (Somerville and Ogren, 1981; Wu et al., 2015). Biochemical analysis demonstrated that cytosolic AtSHMT4 likely functions in methionine and SAM biosynthesis, whereas AtSHMT5–7 showed catalytic inactivity resulting from weak or no PLP binding, suggesting they might have other biological functions, such as regulatory functions or forming multi-protein complexes (Guiducci et al., 2019; Nogués et al., 2022). The nuclear-localized AtSHMT6 has been linked to protein ubiquitination, histone binding, and DNA methylation. Additionally, plants overexpressing *AtSHMT6* exhibited increased flower, silique, seed, and root size (Singh et al., 2024). The function of nuclear-targeted AtSHMT7 has been linked to epigenetic regulation of sulfur and SAM homeostasis (Huang et al., 2016).

Several studies have explored the involvement of SHMTs in plant responses to abiotic stress across different plant species. For example, the rice chloroplast-localized OsSHMT3 has been shown to play an important role in conferring tolerance toward salt stress when overexpressed in *Arabidopsis* (Mishra et al., 2019). Similarly, the cotton mitochondria-localized GhSHMT11 has also been shown to be involved in salt stress, and its overexpression in *Arabidopsis* enhanced salt tolerance by reducing reactive oxygen species (ROS) accumulation (Yan et al., 2024). An endoplasmic reticulum-localized SHMT in rice was shown to scavenge excess ROS that accumulates under chilling stress, thereby increasing tolerance to low temperature (Fang et al., 2020). Recent studies have shown that *SHMT* genes in alfalfa and tomato are upregulated in response to high salt, alkaline conditions, and high and low temperatures, suggesting their involvement in responses to abiotic stresses (Gao et al., 2024).

Reports of *SHMT* involvement in biotic stress responses in plants have also increased in recent years. The rice *OsSHMT1* was found to be a key player in mediating resistance to sheath blight through the glutathione (GSH)-ascorbic acid (AsA) antioxidant system (Wang et al., 2021). The wheat *TaSHMT3A-1* was found to play an important role in resistance to the fungal pathogen *Fusarium graminearum* (Hu et al., 2022). Loss-of-function *Atshmt6* mutants exhibited reduced ethylene and lignan production, whereas overexpression led to an increase in these compounds coupled with enhanced resistance to bacterial (*Pseudomonas syringae*) and fungal (*F. oxysporum*) pathogens (Singh et al., 2024). The soybean *GmSHMT08* was reported to play a major role in resistance to the soybean cyst nematode (SCN, Heterodera glycines Ichinohe), possibly through perturbations to folate binding and intracellular redox homeostasis (Liu et al., 2012; Lakhssassi et al., 2019). Overexpression of *AtSHMT4*, the *Arabidopsis* ortholog of *GmSHMT08*, also enhanced *Arabidopsis* resistance against the beet cyst nematode (Zhao et al., 2022).

Little is known about the function of *SHMTs* in soybean. The soybean *SHMT* gene family consists of 14 family members encoding proteins that vary in size between 471 amino acids (aa) and 603 aa, and one truncated SHMT that is 244 aa. These SHMTs are localized to different compartments, including five in mitochondria, four in the nucleus, two in the chloroplast, and two in the cytosol (Wu et al., 2016). Besides a general function in 1C metabolism and photorespiration in mitochondria, only *GmSHMT08* has been reported to function in SCN resistance in soybean (Liu et al., 2012; Lakhssassi et al., 2019). *GmSHMT08* (*Glyma.08G108900*) is located on chromosome 08 and has a closely related paralog, *GmSHMT05* (*Glyma.05G152100*), on chromosome 05; both code for SHMTs predicted to function in the cytosol. *GmSHMT05* and *GmSHMT08* are believed to result from a recent duplication event in the *Glycine* lineage (Schmutz et al., 2010). The absence of the SCN-resistant *GmSHMT08* allele in wild soybeans suggests it likely arose through artificial selection during soybean domestication (Wu et al., 2016).

Several *GmSHMT08* haplotypes differing by single nucleotide polymorphisms have been reported in soybean: *GmSHMT08-a*, *GmSHMT08-b*, and *GmSHMT08-*c (Patil et al., 2019). *GmSHMT08-b* is present in susceptible soybean cultivars such as Essex and Williams 82. GmSHMT08-a differs from GmSHMT08-b by two amino acid substitutions at positions 130 (P130R, proline to arginine) and 358 (N358Y/H, asparagine to tyrosine/histidine) and is present in SCN-resistant cultivars such as Peking and Forrest. These two amino acid changes in GmSHMT08-a result in the gain-of-function in SCN resistance observed in soybean cultivar Forrest compared to the susceptible cultivar Essex (Liu et al., 2012; Wu et al., 2016; Patil et al., 2019). *GmSHMT08-c* has only been reported in the plant introduction (PI) 437654, a broad-spectrum source of SCN resistance. In addition to the amino acid substitutions at residues 130 and 358, GmSHMT08-c has an additional substitution at amino acid 37 (Patil et al., 2019). An epistatic interaction between *GmSHMT08-a* (also known as *Rhg4*) and *GmSNAP18-a*, an α-SNAP (soluble NSF attachment protein) (also known as *rhg1-a*) governs resistance to SCN HG-type 0 (Liu et al., 2012; Kandoth et al., 2017; Liu et al., 2017).

The X-ray crystal structures of Forrest GmSHMT08-a and Essex GmSHMT08-b were recently solved. Both are tetramers composed of two obligate dimers. In the Essex GmSHMT08-b, a mobile loop near the entrance of the THF binding site was identified in the THF-free enzyme structure, which becomes ordered upon the binding of 5-formyl-THF, consistent with high-affinity binding. In Forrest GmSHMT08-a, the two amino acid polymorphisms were found to impair the mobility of this loop, resulting in reduced folate binding affinity and impaired enzyme activity (Korasick et al., 2020).

To further characterize the role of cytosolic *GmSHMTs* in soybean growth and development, we carried out structural and biochemical analysis of GmSHMT05, characterized the developmental phenotypes of two soybean *Gmshmt08* mutants (Kandoth et al., 2017), and used CRISPR/Cas9 gene editing to validate the role of cytosolic *GmSHMTs* in soybean and their function in soybean root growth and defense.

## Results

### Sequence similarity and tissue-specific expression of cytosolic *GmSHMTs* during soybean development

**Figure 1:**
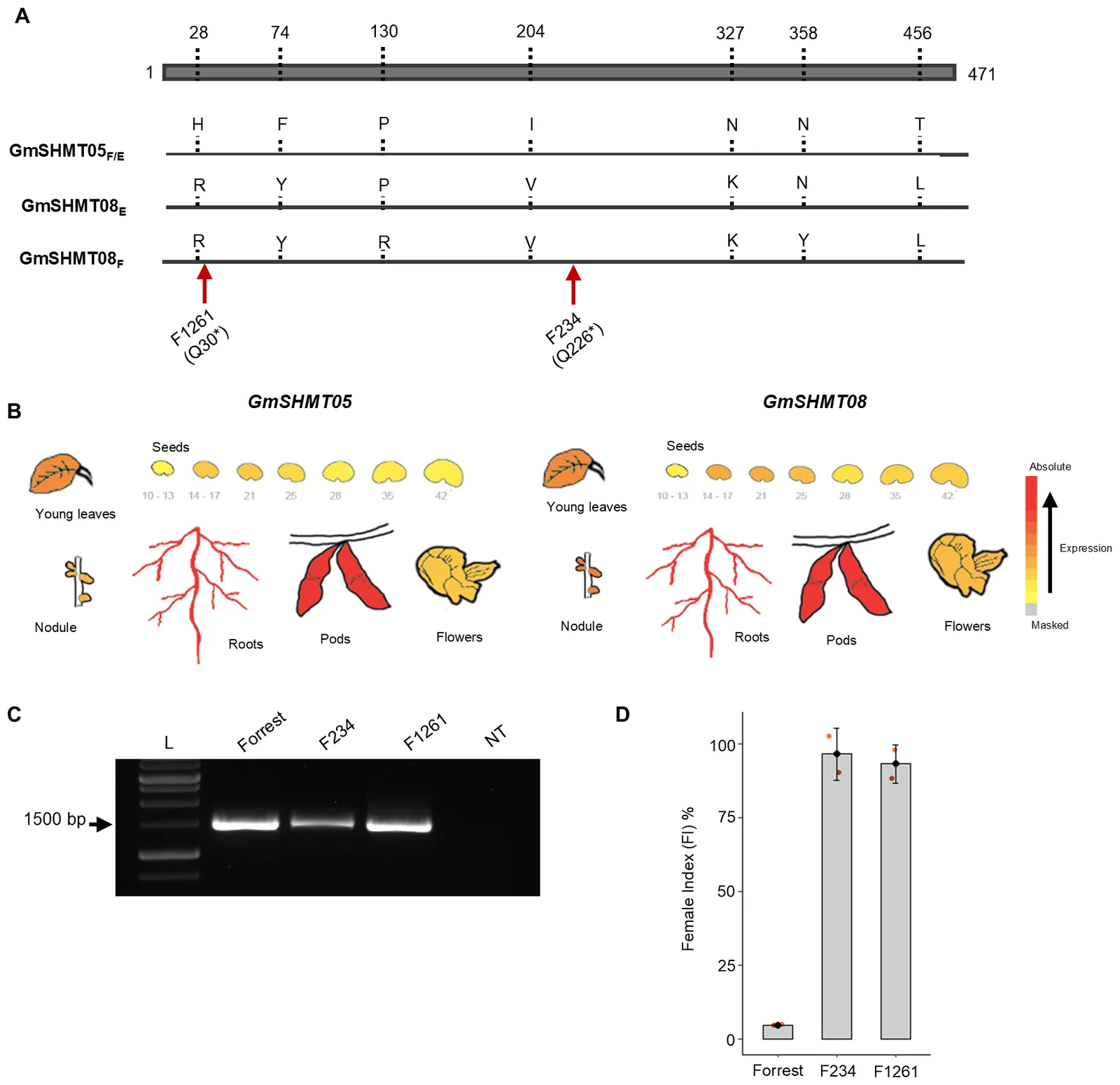
Comparative expression and structural variation of soybean cytosolic SHMTs. **(A)** A schematic diagram of amino acid (aa) sequences of GmSHMT05 and GmSHMT08. Both proteins are 471-aa long. GmSHMT05 in Essex **(E)** and Forrest **(F)** are identical. There are five non-synonymous aa polymorphisms between GmSHMT05 and Essex GmSHMT08-b, and seven non-synonymous aa polymorphisms between GmSHMT05 and Forrest GmSHMT08-a. The Forrest GmSHMT08-a and Essex GmSHMT08-b only differ by two non-synonymous aa polymorphisms. The red arrows indicate two *Gmshmt08-a* EMS mutants, F1261 and F234, with nonsense mutations at aa positions 30 (Q30*) and 226 (Q226*), respectively. **(B)** *GmSHMT05* and *GmSHMT08* expression profile. Both genes are highly expressed in roots and pods (adapted from https://www.soybase.org/). **(C)** The amplification of cDNA of *GmSHMT08-a* from Forrest, F234, and F1261. **(D)** The female index of HG type 0 SCN on Forrest, F234, and F1261. Data averaged from two independent biological replicates.

**Figure 2:**
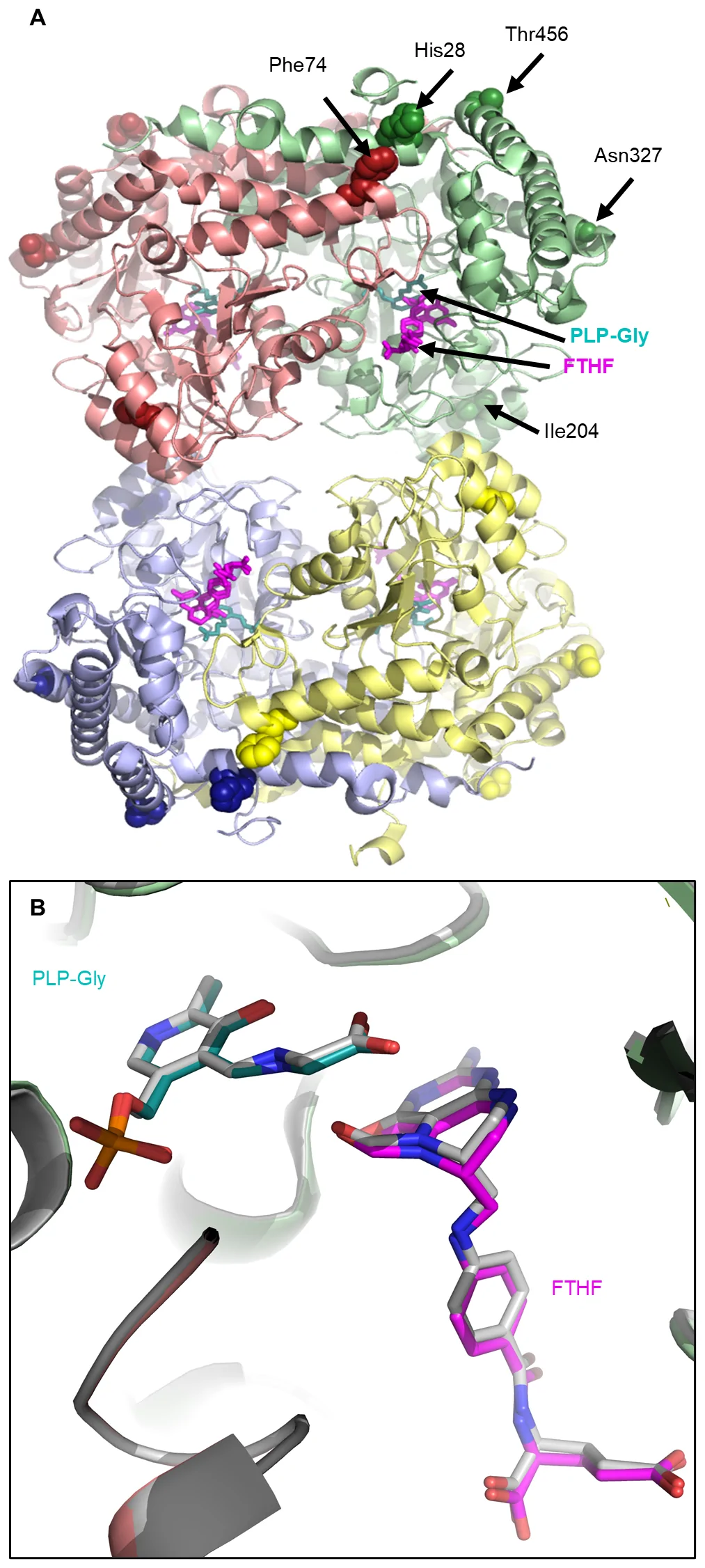
Crystal structure of the GmSHMT05 tetramer. **(A)** Ribbon diagram of the ternary complex of GmSHMT05 with the reaction intermediate PLP-glycine (dark cyan) and 5-formyl-tetrahydrofolate (magenta) bound in each of the four active sites. The four polypeptide chains of the tetramer **(A–D)** are shown in contrasting colors: salmon, green, blue, and yellow. Residues of GmSHMT05 that differ from GmSHMT08-b are shown in spheres (all chains) and labeled for one chain. **(B)** Overlay of the PLP-Gly and folate-binding region for one active site of GmSHMT05 with that of GmSHMT08-b (PDB ID 6UXJ). Colors for GmSHMT05 and its ligands are as shown in panel **(A)**; GmSHMT08-b is in gray. High structural similarity (0.38 Å root-mean-square deviation for 1,892 C{σβ}α{/σβ} atoms) is seen in both the proteins and the binding pose of the ligands, reflecting the high sequence identity between the two enzymes.

The soybean *SHMT* gene family includes two members, *GmSHMT05* (*Glyma.05G152100*) and *GmSHMT08* (*Glyma.08G108900*), that code for cytosol-localized isoforms of the enzyme. GmSHMT05 and GmSHMT08 share 98.5% amino acid identity, differing by only five amino acids (**Figure 1A**; surface residues; **Figure 2**), and exhibit overlapping expression patterns during soybean growth and development, with the highest level of expression in pods and roots (**Figure 1B**) (Severin et al., 2010). The GmSHMT05 protein sequence is identical between the SCN-resistant soybean cv. Forrest and the SCN-susceptible cv. Essex (**Figure 1A**). In contrast, the Forrest and Essex *GmSHMT08* alleles code for proteins that differ by two amino acid changes: the SCN-resistant GmSHMT08-a contains arginine and tyrosine at positions 130 and 358 of the polypeptide chain, respectively, compared to proline and asparagine in SCN-susceptible GmSHMT08-b (i.e., P130R and N358Y). Previously, we identified an allelic series of soybean cv. Forrest ethyl methanesulfonate-derived *Gmshmt08-a* mutants, including mutants F1261 and F234 that have nonsense mutations resulting in premature stop codons at amino acid positions 30 (Q30*) and 226 (Q226*), respectively (**Figure 1A**) (Liu et al., 2012; Kandoth et al., 2017). The F234 mutant has a reduced level of expression (**Figure 1C**) and, if made, the truncated protein would represent less than half of the intact protomer, thereby preventing dimerization and tetramerization; would lack the key active site residue Lys244 responsible for covalent linkage to the PLP cofactor; and would be predicted to have no activity (**Supplementary Figure 1**). Similarly, despite the production of transcripts, the F1261 mutation would result in a severely truncated 30-aa protein (**Figure 1C**). Consistent with a loss-of-function in SCN resistance, both *Gmshmt08-a* mutants are highly susceptible to SCN, exhibiting female indices (FI) greater than 90% (**Figure 1D**). These mutants were backcrossed twice to Forrest to reduce background mutations. A 50K SNP analysis confirmed the mutants were derived from Forrest, with F234 and F1261 exhibiting 98% and 97.5% genetic similarity to Forrest, respectively, and 98.5% similarity to each other (**Supplementary Figure 2**). Both mutant alleles were included for analysis of soybean growth and development phenotypes to confidently attribute any phenotypic effects to *Gmshmt08-a*.

### Structural and biochemical characterization of GmSHMT05

GmSHMT05 has five amino acid differences compared to GmSHMT08-b (**Figure 2A**). To investigate the potential impacts of these residue changes on the 3D structure and function of GmSHMT05, we expressed the protein recombinantly in *E. coli* and used X-ray crystallography and several biochemical assays for further characterization. Two high-resolution crystal structures of GmSHMT05 were determined in complex with various ligands: 1) a complex with the PLP-glycine (PLP-Gly) external aldimine (1.93 Å resolution); and 2) a ternary complex with PLP-Gly and the substrate analog 5-formyl THF (1.96 Å resolution) (**Supplementary Table 1**). The crystal structures show that GmSHMT05 adopts the conserved tetrameric assembly seen in the structure of GmSHMT08-b (Korasick et al., 2020) and other eukaryotic SHMT family members. The GmSHMT05 tetramer is composed of two obligate dimers, formed by two tightly intertwined protomers. The obligate dimer has two active sites, each containing residues from both protomers. The five amino acid differences between GmSHMT05 and GmSHMT08-b are seen to affect surface-exposed residues (**Figure 2A**) without known functional roles and do not cause any noticeable structural changes relative to GmSHMT08-b. Moreover, the active site and bound ligands in the GmSHMT05 structures superimpose with high similarity to those of GmSHMT08-b (**Figure 2B**).

To investigate the folate-binding affinity of GmSHMT05, we determined binding constants in a two-substrate system that exploits a spectrophotometrically observable change associated with folate binding (**Supplementary Table 2**; **Supplementary Figures 3A, B**). We observed saturable binding of FTHF to GmSHMT05 with glycine-dependent affinity, similar to what was previously observed in GmSHMT08-b (Korasick et al., 2020). We also characterized the oligomeric state of GmSHMT05 by size exclusion chromatography (**Supplementary Figure 3C**), which shows it forms a stable tetramer in solution. GmSHMT05 also displays nearly identical kinetic parameters to GmSHMT08-b when assaying the conversion of THF to 5,10-methylene THF (**Supplementary Figures 3D, E**) as well as an alternative, THF-independent enzymatic reaction: retro-aldol cleavage of the β-hydroxy-amino acid L-phenylserine (**Supplementary Table 2**). Together, these results suggest that GmSHMT05 is a functional enzyme, likely to provide SHMT activity in the soybean cytosol.

### GmSHMT05 exhibits weak self-association and interaction with GmSHMT08

Because cytosolic GmSHMT05 and GmSHMT08 are highly similar and co-expressed, we tested whether they could undergo heterotypic subunit interactions that might permit mixed oligomer formation in the cytosol. A GAL4-based yeast two-hybrid system was used to assess potential protein-protein interactions. The SHMT bait constructs showed no toxicity or self-activation when co-transformed with empty prey vectors (**Supplementary Figure 4**). All bait and prey plasmids were successfully co-transformed into yeast and grew on SD/-Leu/-Trp medium, and positive and negative controls behaved as expected. Yeast co-expressing pGBKT7-SHMT5 with either pGADT7-SHMT5 or pGADT7-SHMT8 grew and developed blue coloration on SD/-Leu/-Trp/-His/X-α-Gal but failed to grow on SD/-Leu/-Trp/-His/-Ade/X-α-Gal, indicating weak but specific interactions between the GmSHMT05 subunits as well as between GmSHMT05 and GmSHMT08.

### Effect of *GmSHMT08-a* on soybean above-ground growth and development

To assess any phenotypic effects of *GmSHMT08-a* on soybean growth and development, wild-type soybean cv. Forrest and the two Forrest *Gmshmt08-a* mutants (F234 and F1261) were evaluated under field and greenhouse (GH) conditions, followed by multi-location yield trials.

**Figure 3:**
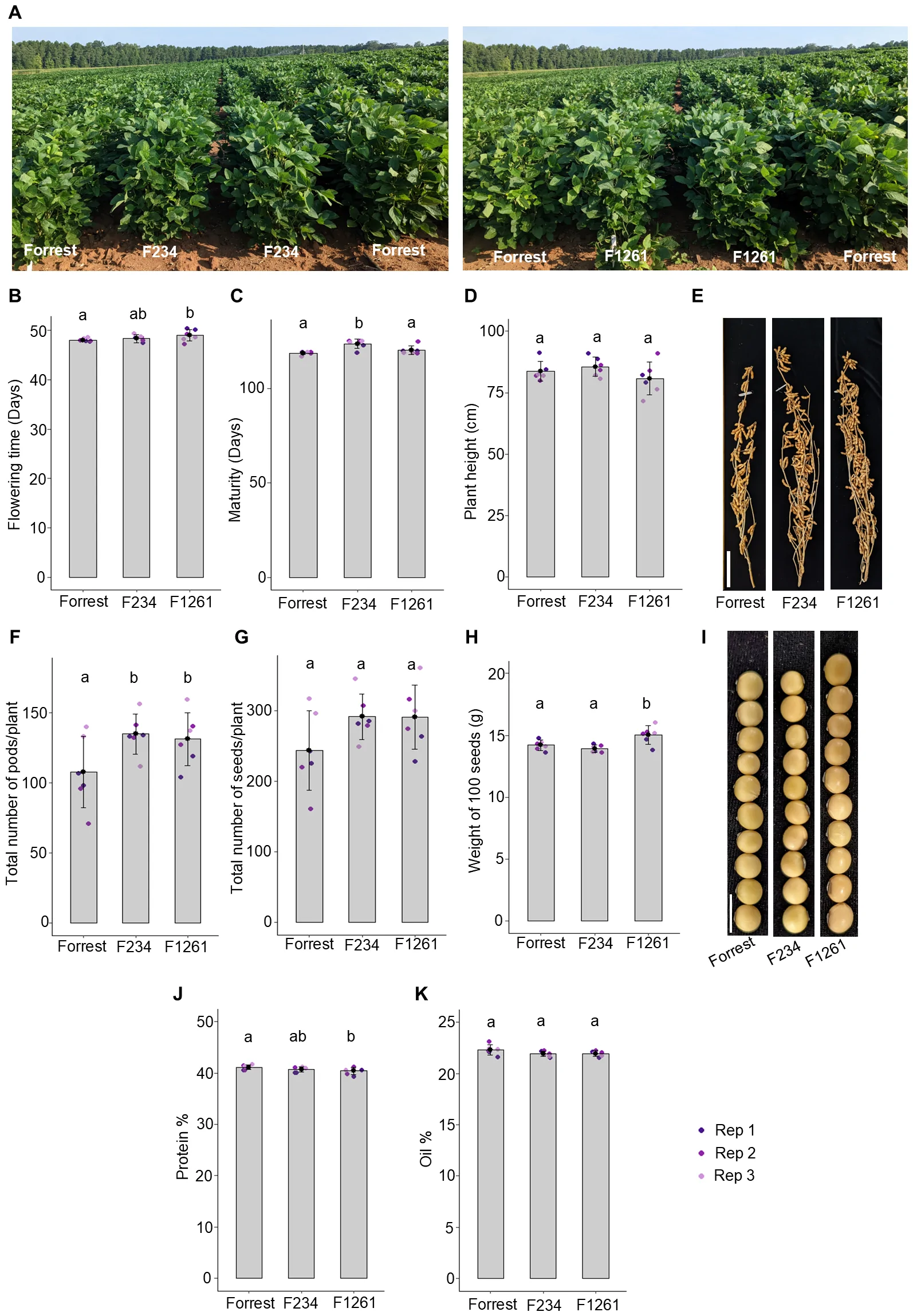
The comparison of agronomically important phenotypic characters between Forrest and two Forrest *Gmshmt08-a* mutants, F234 and F1261, under field conditions. **(A)** The growth of Forrest, F234, and F1261 at flowering time. **(B)** Flowering time. **(C)** Time to maturity. **(D)** Height at maturity. **(E)** Phenotypes of Forrest, F234, and F1261 under the field conditions at maturity. **(F)** Total number of pods per plant. **(G)** Total number of seeds per plant. **(H)** Average weight of 100 seeds. **(I)** Length of 10 seeds. **(J)** Average protein content, dry basis (%). **(K)** Average oil content, dry basis (%). Means were compared using ANOVA followed by an LSD test at *p <*0.05. Different letters indicate significant differences. All data are presented as mean ± SD. Each point represents the average of a single row within a plot per genotype (two rows per plot). Colors denote replications.

In the field-grown environment, compared to Forrest, *Gmshmt08-a* mutant plants did not show noticeable differences in aboveground growth at the flowering stage (**Figure 3A**), although F1261 exhibited a significant delay (one day) in flowering time (**Figure 3B**; **Supplementary Table 3**) and F234 showed a 5-day delay in maturity relative to Forrest (**Figure 3C**; **Supplementary Table 3**). No significant differences in plant height were observed among the genotypes (**Figure 3D**; **Supplementary Table 3**). Interestingly, both F234 (135) and F1261 (131) produced significantly more pods per plant compared to Forrest (108), and seed numbers were numerically higher, but not significantly so, in both mutants (F234 = 291; F1261 = 290) compared to Forrest (244) (**Figures 3E–G**). The observed increase in the number of seeds per plant was not due to a higher number of seeds per pod (**Supplementary Table 3**). Seed size of F1261 (15 g) was also affected, showing a significantly greater 100-seed weight than the other genotypes (F234 = 13.9 g; Forrest = 14.2 g) (**Figures 3H, I**; **Supplementary Table 3**). No significant differences among genotypes were detected in protein, oil, amino acid, and fatty acid content (**Figures 3J, K**; **Supplementary Tables 3–5**).

When evaluated under greenhouse conditions, Forrest and *Gmshmt08-a* mutant plants appeared similar in aboveground growth at flowering (**Supplementary Figure 5A**), although F1261 exhibited a significant delay (2 days) in flowering time (**Supplementary Figure 5B**; **Supplementary Table 6**). Maturity and height were not significantly different among genotypes (**Supplementary Figures 5C, D**; **Supplementary Table 6**). The mutants had numerically higher pod (F234 = 298; F1261 = 295) and seed numbers (F234 = 628; F1261 = 625) per plant compared to Forrest (pod number = 276; seed number = 602); however, these values were not statistically significant (**Supplementary Figures 5E, F**; **Supplementary Table 6**). The observed increase in the number of seeds per plant was not due to a higher number of seeds per pod (**Supplementary Table 6**). Differences in seed size were also not statistically significant, even though mutants displayed higher average values (Forrest = 10.6 g; F234 = 10.8 g; F1261 = 10.9 g) (**Supplementary Figure 5G**; **Supplementary Table 6**). Neither protein nor oil content differed significantly (**Supplementary Figures 5H, I**; **Supplementary Table 6**), and no variation was observed in amino acid or fatty acid profiles under greenhouse conditions (**Supplementary Tables 7**, **S8**).

### Effect of *GmSHMT08-a* on soybean yield performance

Based on the observed increase in pod and seed number in the mutant lines in field and greenhouse phenotyping trials, yield (kg/ha) and agronomic performance were further evaluated across four field locations: two locations in GA and two locations in MO. Yield was significantly affected by genotype, location, and their interaction (genotype × location) (*p <*0.05; **Table 1**). Compared to Forrest across locations, F234 had numerically higher yield in Athens (F234 = 4321.8; Forrest = 4046.8) and Plains (F234 = 3844.5; Forrest = 3472), and significantly higher yield in Portageville (F234 = 3922.5; Forrest = 3211), whereas F1261 had numerically higher yield in Plains (F1261 = 3913.1; Forrest = 3472) and Portageville (F1261 = 3490.5; Forrest = 3211). Both F234 and F1261 showed delayed maturity (3 days) compared to Forrest across locations. Plant height did not differ among genotypes in Athens or Fisk, but F234 was significantly taller than Forrest in Portageville. No location effect was detected for 100-seed weight. Similar to field and greenhouse studies, F1261 (14.6 g) had significantly greater 100-seed weight than Forrest (14.2 g) and F234 (14 g). Protein content was significantly higher in Forrest and F234 than in F1261, whereas oil content did not differ among genotypes. Amino acid analysis revealed no differences for cysteine, methionine, threonine, or histidine; however, lysine, isoleucine, leucine, valine, and phenylalanine were significantly reduced in F1261 compared to Forrest. The level of all amino acids was numerically lower in both mutants, except for cysteine (**Supplementary Table 9**). Among major fatty acids, only linolenic acid varied significantly across locations, with no consistent genotype-dependent trend (**Supplementary Table 10**).

**Table 1:** Mean performance for yield and phenotypic traits of Forrest and the *Gmshmt08* mutants, F234 and F1261, evaluated across four locations in summer 2025.

Source of variation	Yield (kg/ha)	Maturity (Days)	Plant height	Seed weight	Protein^d^	Oil^d^
	kg ha^−1^	d after 31 Aug.	Cm	g per 100 seeds	(%)	(%)
Athens, GA						
Forrest	4,046.8 ± 218.1^a^	–	104.6 ± 1.7^a^	–	–	–
F234	4,321.8 ± 306.4^a^	–	102.9 ± 3.3^a^	–	–	–
F1261	3,992.1 ± 241.4^a^	–	98.3 ± 3.5^a^	–	–	–
Plains, GA						
Forrest	3,472 ± 108^a^	–	ND	–	–	–
F234	3,844.5 ± 206.3^a^	–	ND	–	–	–
F1261	3,913.1 ± 271.6^a^	–	ND	–	–	–
Fisk, MO						
Forrest	4,449.8 ± 331.3^a^	–	107 ± 8.7^a^	–	–	–
F234	4,410.9 ± 75.9^a^	–	101 ± 9.6^a^	–	–	–
F1261	4,115.2 ± 247.3^a^	–	104.3 ± 9.3^a^	–	–	–
Portageville, MO						
Forrest	3,211 ± 460.4^a^	–	52.7 ± 18.9^a^	–	–	–
F234	3,922.5 ± 445.4^b^	–	72 ± 3.6^b^	–	–	–
F1261	3,490.5 ± 289.5^ab^	–	62 ± 10.2^ab^	–	–	–
Genotype main effect						
Forrest	3,794.9 ± 570	32 ± 6^a^	88.1 ± 28.6	14.2 ± 1.4^ab^	40.5 ± 1.1^a^	22 ± 0.4^a^
F234	4,124.9 ± 357	35 ± 5^b^	92 ± 15.9	14 ± 1.1^b^	40.1 ± 0.9^a^	22 ± 0.6^a^
F1261	3,877.7 ± 332.5	35 ± 6^b^	88.2 ± 21.1	14.6 ± 1.3^a^	39.2 ± 1.4^b^	22 ± 0.7^a^
Location main effect						
Athens	4,120.2 ± 270.8	28 ± 3^a^	101.9 ± 3.8	14.1 ± 0.5^b^	39 ± 0.9^b^	22.2 ± 0.4^a^
Plains	3,743.2 ± 272.5	ND	ND	15.6 ± 0.6^a^	41.1 ± 1^a^	21.6 ± 0.6^b^
Fisk	4,325.3 ± 263.2	40 ± 2^b^	104.1 ± 8.3	ND	ND	ND
Portageville	3,541.3 ± 468.9	33 ± 2^c^	62.2 ± 13.7	13 ± 0.5^c^	39.7 ± 0.7^b^	22.2 ± 0.2^a^
ANOVA (p-values)						
Genotype (G)	4.8 × 10^-3**^	3 × 10^−4***^	0.4	2.8 × 10^−2*^	2.1 × 10^−2*^	0.9
Location (L)	3.3 × 10^-6***^	6.2 × 10^−10***^	1.5 × 10^−8***^	1 × 10^−6***^	7.1 × 10^−4***^	2.4 × 10^−2*^
G × L	4.5 × 10^-2*^	0.2	4.5 × 10^−2*^	0.8	0.7	0.6
Block (L)	1.8 × 10^-2*^	0.1	2.4 × 10^−2*^	0.4	0.8	0.6
LSD_(0.05)_	373.1	1.4	11.8	0.5	0.9	0.5

^a-c^Mean comparison within each location was done using ANOVA followed by Least Significant Difference (LSD) test at p <0.05. Different letters indicate significant differences. All data arepresented as mean ± SD. , if p <0.001; , if p <0.01; , if p <0.05. ND, data not available.

^d^Protein and oil (%) → Values are expressed on a dry matter basis.

Mean comparison within each location was done using ANOVA followed by Least Significant Difference (LSD) test at *p* <0.05. Different letters indicate significant differences. All data are presented as mean ± SD. ***, if *p* <0.001; **, if *p* <0.01; *, if *p* <0.05. ND, data not available.

### Effect of cytosolic *GmSHMTs* on root growth and development

**Figure 4:**
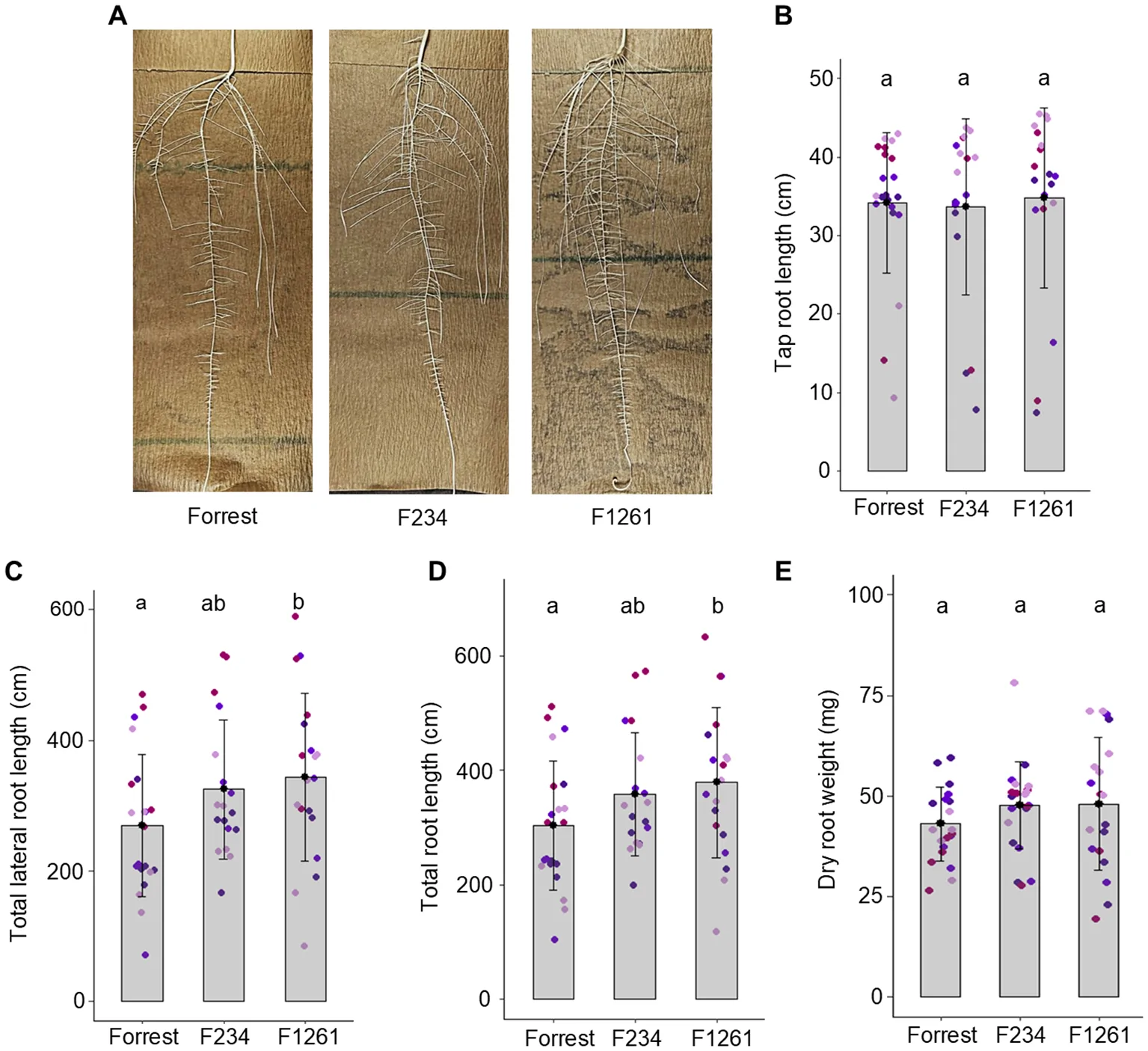
The comparison of root traits between Forrest and two Forrest *Gmshmt08-a* mutants, F234 and F1261. **(A)** Ten-day-old roots grown in pouches for root length measurements. **(B)** Tap root length of Forrest, F234, and F1261. **(C)** Total lateral root length of Forrest, F234, and F1261. **(D)** Total root length of Forrest, F234, and F1261. **(E)** Dry root weight of Forrest, F234, and F1261. All data are presented as mean ± SD. Means were compared using ANOVA followed by an LSD test at *p <*0.05. Different letters indicate significant differences. One dot represents an individual root. The color of dots in each comparison represents the four independent biological replicates. For root length measurements, n = 18–21 roots for each genotype. For dry weight, n = 15 for each genotype.

Representative root systems of Forrest, F234, and F1261 plants grown in pouches are shown in **Figure 4A**. Analysis of variance revealed no significant differences in taproot length among Forrest, F234, and F1261 (**Figure 4B**; **Supplementary Table 11**). However, both F234 and F1261 showed an increase in lateral (Forrest = 269.2 cm; F234 = 324.4 cm; F1261 = 343.9 cm) and total root length (Forrest = 303.3 cm; F234 = 358.1 cm; F1261 = 378.7 cm) as well as numerically higher root dry weight (Forrest = 43 cm; F234 = 47.6 cm; F1261 = 47.9 cm) (**Figure 4E**; **Supplementary Table 11**) compared to Forrest, although the increase in root length was statistically significant only for F1261 (**Figures 4C, D**; **Supplementary Table 11**).

### Targeted editing of cytosolic *SHMT*s in roots of soybean composite plants

**Figure 5:**
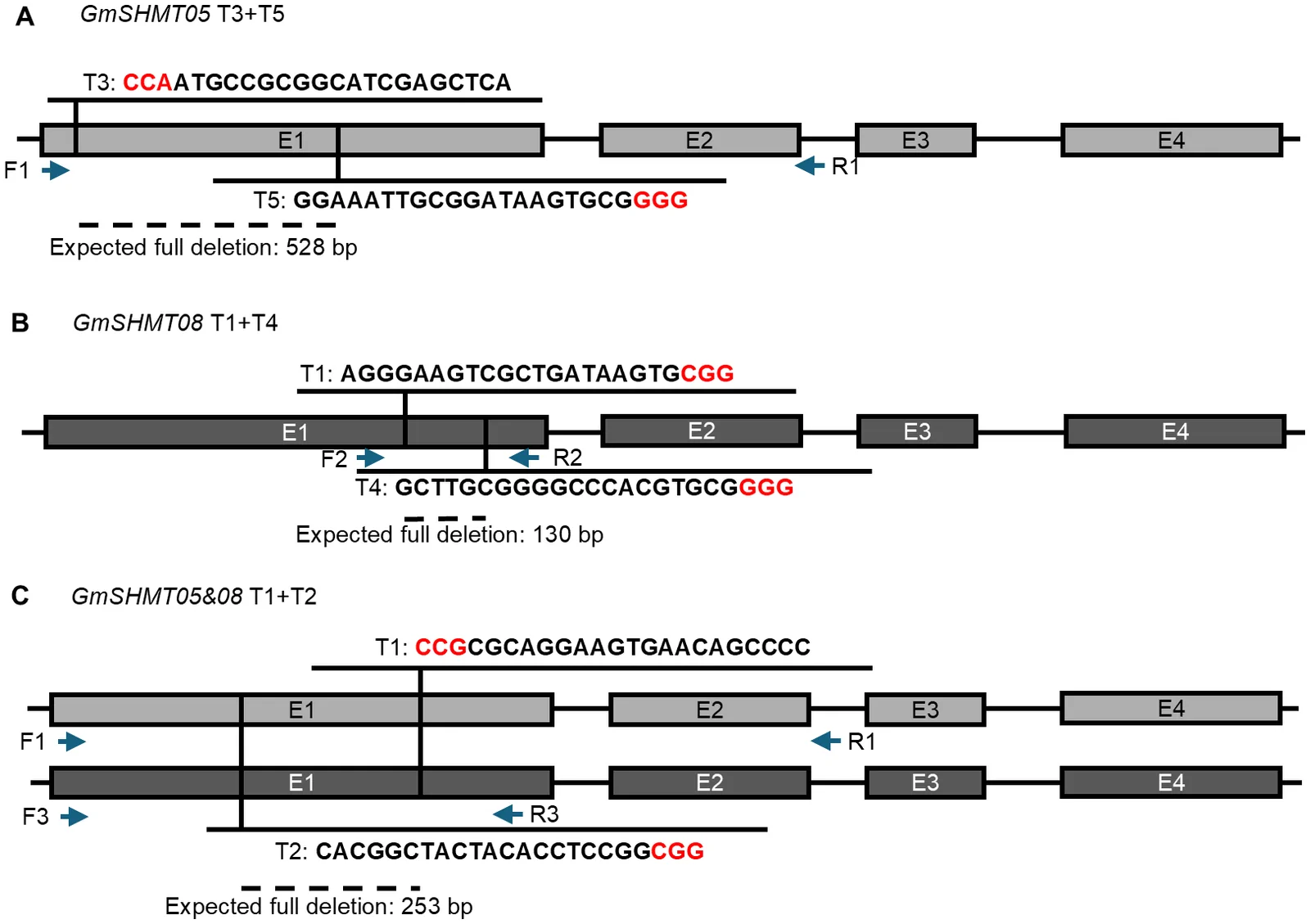
CRISPR/Cas9 editing of *GmSHMTs* in Forrest, F234, and Essex. Diagram showing the position of the single guide RNA (sgRNA) sequences designed to edit **(A)** *GmSHMT05*, **(B)** *GmSHMT08*, and **(C)** *GmSHMT05* and *GmSHMT08* (*GmSHMT05&08*) simultaneously. PAM sequences are in red. sgRNA sequences are bolded. F1 and R1 primers flanking two target sites amplify the region in *GmSHMT05* to confirm edits in hairy roots inoculated by K599 carrying CRISPR/Cas9-*GmSHMT05*-T3+T5 and CRISPR/Cas9-*GmSHMT05&08*-T1+T2. F2 and R2 primers flanking two target sites amplify the region in *GmSHMT08* in roots edited by the CRISPR/Cas9-*GmSHMT08*-T1+T4 construct. F3 and R3 primers were utilized to amplify the region in *GmSHMT08* targeted by CRISPR/Cas9-*GmSHMT05&08*-T1+T2.

To further assess the role of cytosolic *SHMT*s in soybean root growth, we coupled CRISPR/Cas9 editing with hairy root transformation to knock out one or both cytosolic *SHMT*s in roots of soybean composite plants (**Figure 5**). We hypothesized that at least one cytosolic *SHMT* would be necessary for root growth and that *GmSHMT08-a* would not complement the loss of function of *GmSHMT05*. To test this, *GmSHMT05* was edited in the Forrest and F234 backgrounds. Forrest carries *GmSHMT08-a* and *GmSHMT05*, whereas F234 carries the *Gmshmt08-a* nonsense allele and *GmSHMT05*. Targeted editing of *GmSHMT05* was done using the CRISPR/Cas9-*GmSHMT05*-T3+T5 editing construct. This construct targeted two regions in exon 1 of *GmSHMT05* that were 528 bp apart (**Figure 5A**) and was specific to *GmSHMT05*. Composite soybean plants of Forrest and F234 were generated through co-cultivation with *R. rhizogenes* K599 carrying either CRISPR/Cas9-*GmSHMT05*-T3+T5 or an empty vector (EV) control.

**Figure 6:**
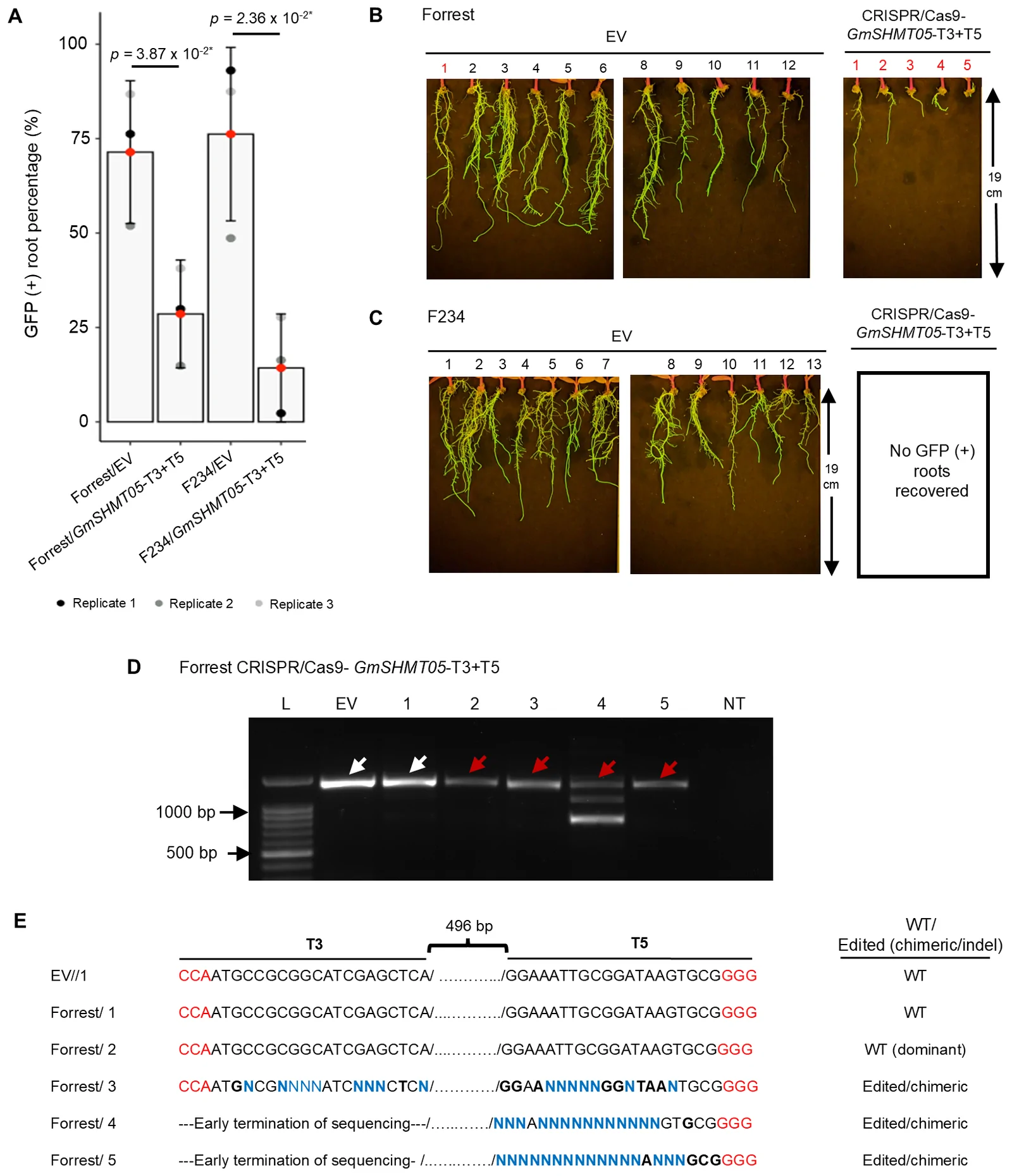
Targeted genome editing of *GmSHMT05* in Forrest and F234 by the CRISPR/Cas9-*GmSHMT05*-T3+T5 dual gRNA system in replicate 1. **(A)** The number of composite plants with GFP (+) roots relative to the total number of plants inoculated as a percentage in Forrest and F234. Data are presented as means ± SD of three biological replicates for each CRISPR/Cas9 target. Statistical analysis was performed by a two-tailed Student’s *t*-test (*p <*0.05). **(B)** Plants with GFP (+) roots inoculated with K599 carrying EV and CRISPR/Cas9-*GmSHMT05*-T3+T5 in Forrest. Genomic DNA was extracted from transgenic hairy roots from plants indicated in redcolor. **(C)** Plants with GFP (+) roots inoculated with K599 carrying EV and CRISPR/Cas9-*GmSHMT05*-T3+T5 in F234. **(D)** Gel image of *GmSHMT05* amplification flanking the T3 and T5 region using F1 and R1 in Forrest. Red arrows indicate the bands with edits, and white arrows indicate the band with WT sequences. **(E)** Sequences of extracted bands indicated by arrows in **(D)**. The bolded bases indicate multiple peaks at that position, but the dominant base matches the WT sequence. The blue-colored “N” denotes chimeric peaks where bases differ from WT, representing a mixture of WT bases and newly introduced bases across multiple amplified alleles. L, 100 bp ladder; WT, wild type; NT, no template.

Composite plants with transgenic roots were identified by the expression of green fluorescent protein (GFP). Composite plants with at least one GFP (+) root longer than 1 cm were considered when calculating the percentage of plants with GFP (+) roots relative to the total number of plants inoculated with K599 (**Figure 6A**). In Forrest, 71% of plants inoculated with K599 carrying the EV had GFP (+) roots. In contrast, only 29% of Forrest composite plants inoculated with K599 carrying CRISPR/Cas9-*GmSHMT05*-T3+T5 constructs had at least one GFP (+) root, showing a significant reduction relative to EV (**Figure 6A**; **Supplementary Table 12**). A similar trend was observed in the F234 genotype. The mean percentage of composite plants with GFP (+) roots inoculated with EV was 76%, whereas the mean percentage of composite plants with GFP (+) roots inoculated with CRISPR/Cas9-*GmSHMT05*-T3+T5 was only 14%, showing a significant reduction in transgenic root formation (**Figure 6A**). This pattern was consistent in three independent biological replicates.

To determine whether *GmSHMT05* was edited in the GFP (+) roots recovered from Forrest and F234 composite plants, genomic DNA was extracted and the region flanking target sites of *GmSHMT05* was amplified. If no editing of *GmSHMT05* occurred, a 1,379 bp product would be amplified using F1 and R1 primers (**Figure 5A**). A full deletion of *GmSHMT05* would result in an 851 bp product. **Figures 6B, C** show the number of GFP (+) roots recovered from Forrest and F234 composite plants, respectively, co-cultivated with K599 carrying either EV or CRISPR/Cas9-*GmSHMT05*-T3+T5 in replicate 1. This figure includes all plants with at least one GFP (+) root emerging from the callus, regardless of the root length. Forrest had five composite plants (29%) with GFP (+) roots compared to 12 plants (79%) with GFP (+) EV roots (**Figure 6B**). A 1,379 bp product was amplified from all five Forrest composite plants with GFP (+) roots (**Figure 6D**). Amplified products were gel extracted and sequenced to confirm whether roots were edited. Plant 1, which had growth similar to EV plants, was not edited (**Figure 6E**; **Supplementary Figure 6**). Plant 2 showed some editing of *GmSHMT05*, but the WT sequence was dominant. Plants 3, 4, and 5 contained chimeric sequences, indicating a mixture of WT and edited cells, explaining their retarded root growth. This pattern was consistent in three biological replicates (**Supplementary Figures 7**–**S10**). No GFP (+) roots were recovered from F234 plants transformed with CRISPR/Cas9-*GmSHMT05*-T3+T5 in replicate 1 (**Figure 6C**). In replicates 2 and 3, very few GFP (+) roots were recovered. A 1,379 bp product was amplified from all roots, and sequencing of the products further confirmed either no editing or a mixture of WT and edited *GmSHMT05* sequences (chimeric) in GFP (+) roots of F234 (**Supplementary Figures 7**, **S8**). Thus, we were unable to recover roots carrying a homozygous deletion of *GmSHMT05* in Forrest and F234, suggesting an essential role for *GmSHMT05* in root growth of these soybean genotypes.

**Figure 7:**
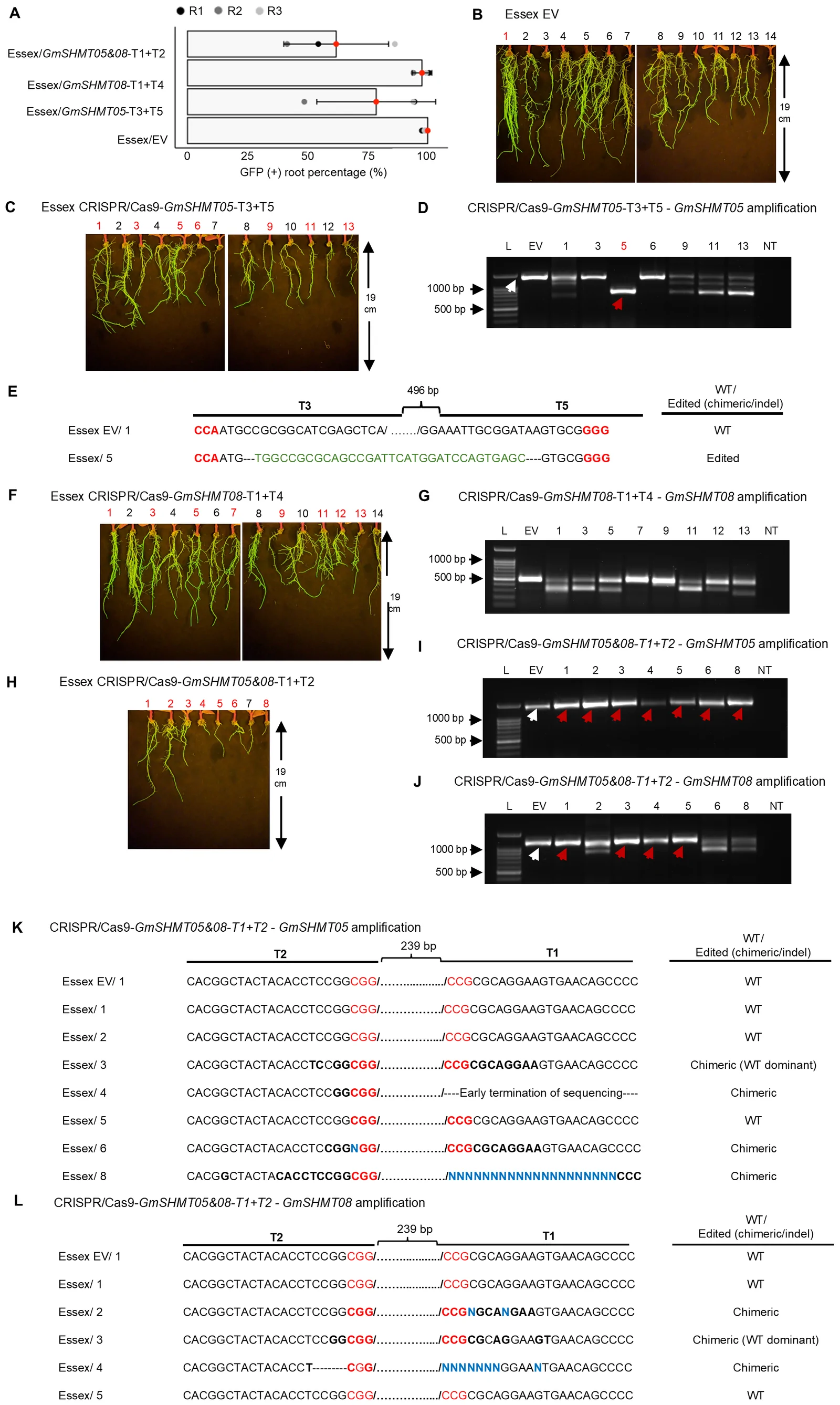
Targeted genome editing of *GmSHMT05* and *GmSHMT08* individually and simultaneously in Essex by the CRISPR/Cas9-*GmSHMT05*-T3+T5, CRISPR/Cas9-*GmSHMT08*-T1+T4, and CRISPR/Cas9-*GmSHMT05&08*-T1+T2 dual gRNA systems in replicate 1. **(A)** The number of composite plants with GFP (+) roots relative to the total number of plants inoculated as a percentage in Essex. Data are presented as means ± SD of three biological replicates for each CRISPR/Cas9 target. Statistical analysis was performed by a two-tailed Student’s *t*-test (*p <*0.05). There was no significant difference between comparisons. **(B)** Plants with GFP (+) roots inoculated with K599 carrying EV. **(C)** GFP (+) roots of Essex plants inoculated with K599 carrying CRISPR/Cas9-*GmSHMT05*-T3+T5. **(D)** Gel image of amplified fragments from genomic DNA extracted from roots transformed with CRISPR/Cas9-*GmSHMT05*-T3+T5. Fragments flanking the T3 and T5 gRNA cleavage sites were amplified using F1 and R1 primers. **(E)** The sequence of the band extracted from plant EV and #5 from the gel image in **(D)**. **(F)** GFP (+) roots of Essex plants inoculated with K599 carrying CRISPR/Cas9-*GmSHMT08*-T1+T4. **(G)** Gel image of amplified fragments from genomic DNA extracted from roots transformed with CRISPR/Cas9-*GmSHMT08*-T1+T4. Fragments flanking the T1 and T4 gRNA cleavage sites were amplified using F2 and R2 primers. **(H)** GFP (+) roots of Essex plants inoculated with K599 carrying CRISPR/Cas9-*GmSHMT05&08*-T1+T2. Roots selected for DNA extraction are in red. **(I)** Gel image of amplified fragments of *GmSHMT05* from genomic DNA extracted from roots transformed with CRISPR/Cas9-*GmSHMT05&08*-T1+T2. Fragments flanking the T1 and T2 gRNA cleavage sites were amplified using F1 and R1 primers. **(J)** Gel image of amplified fragments of *GmSHMT08* from genomic DNA extracted from roots transformed with CRISPR/Cas9-*GmSHMT05&08*-T1+T2. Fragments flanking the T1 and T2 gRNA cleavage sites were amplified using F3 and R3 primers. Arrows indicate the gel bands extracted for direct sequencing. White arrows indicate bands with WT fragments. Red arrows indicate bands with edited sequences. **(K)** The sequences of *GmSHMT05* from plants inoculated with CRISPR/Cas9-*GmSHMT05&08*-T1+T2. **(L)** The sequences of *GmSHMT08* from plants inoculated with CRISPR/Cas9-*GmSHMT05&08*-T1+T2. WT indicates non-edited sequences. Chimeric indicates the mixture of WT and edited sequences. If WT is dominant in chimeric sequences, it is denoted as chimeric (WT dominant). The bolded bases indicate multiple peaks at that position, but the dominant base matches the WT sequence. The blue-colored “N” denotes chimeric peaks where bases differ from WT, representing a mixture of WT bases and newly introduced bases across multiple amplified alleles. L, 100 bp ladder; WT, wild type; NT, no template.

The SCN-susceptible soybean cv. Essex contains *GmSHMT05* and *GmSHMT08-b* (Kang, 2016). We hypothesized that at least one of these cytosolic *SHMT*s would be needed for root growth and that simultaneous editing of both cytosolic *SHMT*s would compromise root growth. We individually edited these genes using CRISPR/Cas9-*GmSHMT05*-T3+T5 and CRISPR/Cas9-*GmSHMT08*-T1+T4 constructs in the Essex background. The CRISPR/Cas9-*GmSHMT08*-T1+T4 was designed to target two regions in exon 1 of *GmSHMT08* that were 130 bp apart (**Figure 5B**) and was highly specific to *GmSHMT08*. The CRISPR/Cas9-*GmSHMT05&08*-T1+T2 construct, consisting of two sgRNA sequences common to both *GmSHMT05* and *GmSHMT08*, targeted two regions that were 253 bp apart in exon 1 in both genes (**Figure 5C**) and was used for simultaneous editing. Composite plants of Essex were generated by inoculation with K599 carrying EV or either CRISPR/Cas9-*GmSHMT05*-T3+T5, CRISPR/Cas9-*GmSHMT08*-T1+T4, or CRISPR/Cas9-*GmSHMT05&08*-T1+T2. Essex showed a 100% transformation rate when inoculated with K599 carrying the EV. The mean percentage of composite plants with GFP (+) roots after inoculation with K599 carrying CRISPR/Cas9-*GmSHMT05*-T3+T5, CRISPR/Cas9-*GmSHMT08*-T1+T4, and CRISPR/Cas9-*GmSHMT05&08*-T1+T2 were 79%, 97%, and 62%, respectively (**Figure 7A**). When *GmSHMT05* and *GmSHMT08-b* were targeted individually, the recovery of transformed roots carrying the construct was higher than when both genes were targeted simultaneously.

GFP (+) roots were recovered from 100% of Essex plants transformed with EV (**Supplementary Table 12**) and exhibited normal root growth (**Figure 7B**). Similarly, a high number of Essex plants with GFP (+) roots were recovered when transformed with either CRISPR/Cas9-*GmSHMT05*-T3+T5 or CRISPR/Cas9-*GmSHMT08*-T1+T4 (**Figure 7A**), and root growth was similar to EV roots (**Figures 7C, F**). Essex plant 5 edited with CRISPR/Cas9-*GmSHMT05*-T3+T5 harbored a full homozygous deletion in *GmSHMT05* (**Figures 7D, E**; **Supplementary Figure 11**) and exhibited normal root growth (**Figure 7C**), demonstrating that *GmSHMT08-b* is sufficient to support root growth in Essex in the absence of *GmSHMT05*.

Roots from Essex plants transformed with CRISPR/Cas9-*GmSHMT08*-T1+T4 also showed successful editing of *GmSHMT08-b* and normal root growth. Edits were confirmed by amplification of multiple bands (**Figures 7F, G**; plants 1, 3, 5, 11, 12, 13). The amplification of a 361 bp fragment indicated a homozygous deletion in *GmSHMT08-b*, and the intensity of the amplified product reflected the prevalence of the *GmSHMT08-b* deletion in the roots, demonstrating that *GmSHMT05* is sufficient to support root growth in Essex in the absence of *GmSHMT08-b*.

When both *GmSHMT0*5 and *GmSHMT08-b* were edited simultaneously in Essex using CRISPR/Cas9-*GmSHMT05&08*-T1+T2, fewer and weaker GFP (+) roots were recovered, compared to when they were edited individually (**Figure 7H**). We amplified and sequenced the fragments of *GmSHMT05* and *GmSHMT08-b* from genomic DNA to confirm the edits (**Figures 7I, J**). When root growth resembled that of the EV control (**Figure 7H**; plants 1, 2), either there were no edits, only one of the cytosolic *SHMT*s had been edited, or if both were edited, the roots contained a mixture of WT and edited sequences (**Figures 7K, L**; **Supplementary Figure 12**, **S13**). Thus, we were unable to recover roots carrying homozygous deletions for both *GmSHMT05* and *GmSHMT08-b* from Essex across three biological replicates (**Supplementary Figures 14**–**S19**), further supporting an essential role for cytosolic *SHMT*s in soybean root growth.

### A role for *GmSHMT08-b* in soybean basal resistance

Previous VIGS and RNAi experiments to silence *GmSHMT08-b* in SCN-susceptible soybean cultivars Essex and Williams 82 resulted in unexpected hypersusceptibility to SCN, suggesting a role for *GmSHMT08-b* in soybean basal resistance (Kandoth and Mitchum, unpublished). However, potential cross-silencing among closely related *GmSHMTs* limited attribution of this phenotype specifically to *GmSHMT08-b*. In another experiment, CRISPR/Cas9 knockout of *GmSHMT08-b* in susceptible soybean hairy roots increased SCN susceptibility. Still, this effect was not reproducible, likely due to root-to-root variability and low infection rates using an *in vitro* soybean hairy root transformation bioassay (Kang, 2016). Therefore, the experiment was repeated in this study using an optimized hairy root transformation protocol and greenhouse SCN bioassay (Gamage et al., 2025). Composite Essex plants carrying EV, CRISPR/Cas9-*GmSHMT05*-T3+T5, or CRISPR/Cas9-*GmSHMT08*-T1+T4 were inoculated with SCN HG type 0 across five biological replicates. ANOVA (genotype as the main effect; replicate as a fixed effect) showed a significant increase in SCN susceptibility of *GmSHMT08-b* edited plants compared with EV and *GmSHMT05***-**edited plants, supporting a role for GmSHMT08-b in soybean basal resistance to SCN (**Supplementary Figure 20**).

## Discussion

The discovery of a major role for the *GmSHMT08-a* gene in resistance to SCN came as quite a surprise to researchers, as it pointed to a novel mechanism of resistance to pathogens (Liu et al., 2012). This led researchers to question how nematodes might be exploiting the housekeeping function of this enzyme for successful parasitism, such that the soybean plant evolved modifications to SHMT activity to disrupt parasitic success, and whether this comes with a cost to the plant. The exact mechanism by which the gain-of-function of *GmSHMT08-a* contributes to SCN resistance remains unclear. However, evidence is mounting in support of a disruption to folate homeostasis as a result of GmSHMT08-a’s inability to bind folate, leading to the production of defense compounds, and perturbations to cellular methylation reactions and intracellular redox status that negatively impact nematode feeding cell establishment (Liu et al., 2012; Korasick et al., 2020). Metabolites downstream of GmSHMT, such as S-adenosyl-L-methionine (SAM), are known to induce ROS in response to pathogen infection. Pathogens may, therefore, target enzymes in this pathway to suppress plant defense responses. For example, *Xanthomonas campestris* pv. *campestris* produces effectors that target methyltransferase complexes to suppress ROS production, promoting susceptibility (Liu et al., 2017). Consistent with this possibility, we demonstrated that targeted knockout of the susceptible *GmSHMT08-b* allele in soybean cv. Essex resulted in an increase in basal plant susceptibility to SCN, supporting a role for *GmSHMT08-b* in soybean basal resistance to SCN. Thus, it is possible that SCN may directly or indirectly target GmSHMT08-b or components of downstream pathways to suppress basal defenses during infection of a susceptible soybean plant, and, in return, GmSHMT08-a gain-of-function modulates 1C metabolism in resistant soybean plants in a way that reinforces its defense against SCN.

Investigations of potential tradeoffs in development for defense against pathogens have received much attention in recent years (Karasov et al., 2017; He et al., 2022). Constitutive defense activation can impose a significant demand for resources, which has been linked to a reduction in plant growth (Smedegaardpetersen and Stolen, 1981). This compromised growth is not seen if the defense genes are instead primed for induced defense against pathogens. A recent study demonstrated that elevated salicylic acid (SA) levels resulted in decreased plant growth and reduced expression of cold-regulated (*COR*) genes, with the suppression being more severe at below-ambient temperatures. Interestingly, constitutive expression of *COR* genes was sufficient to overcome the growth inhibition caused by elevated SA at both ambient and below-ambient temperatures, while still preserving the benefits related to biotic and abiotic stress resistance (Ortega et al., 2024). In some cases, compromised plant fitness can occur in the absence of defense. For example, the fitness of field-grown Arabidopsis was reduced in the absence of NPR-1-dependent SA-mediated defense. Conversely, overexpression of NPR-1 increased pathogen resistance without negatively impacting growth and fitness (Heidel et al., 2004). SHMTs have been increasingly implicated in regulating the plant growth-defense balance. *Atshmt1* mutants showed retarded growth in response to bacterial and fungal pathogens, implicating its role in plant defense, growth, and development (Moreno et al., 2005), and AtSHMT6’s role in regulating ethylene and lignin production was recently linked to plant growth and defense against *Fusarium oxysporum* (Singh et al., 2024).

The genes coding for the soybean cytosolic SHMTs, *GmSHMT05* and *GmSHMT08*, are predominantly expressed at high levels in soybean roots and pods (Severin et al., 2010); as such, any changes in SHMT activity impacting 1C folate metabolism in these tissues may lead to alterations in amino acid metabolism, hormone pathways, and defense-related signaling that could impact root and pod development. The GmSHMT05 protein sequence is identical among resistant and susceptible soybeans and likely serves as a core housekeeping enzyme in soybean, whereas GmSHMT08 differs. The *GmSHMT08-b* susceptible allele encodes a functional enzyme (Korasick et al., 2020), whereas *GmSHMT08-a* encodes an impaired enzyme that cannot bind folate (Korasick et al., 2020), and this leads to a gain-of-function in SCN resistance. Other than a loss of SCN resistance, no obvious growth defects were previously observed in *Gmshmt08-a* mutants, suggesting *GmSHMT05* may fully complement any loss of function of *GmSHMT08-a* (Kandoth et al., 2017). Here we show through protein crystallization and kinetic/binding assays that GmSHMT05 and GmSHMT08-b are indeed similar in overall 3D structure, oligomeric state in solution, and folate binding; thus, the loss of one is likely complemented by the other.

We also used soybean composite plants coupled with CRISPR/Cas9 to evaluate the role of cytosolic *SHMT*s in soybean root growth and to assess whether *GmSHMT08-a* and *GmSHMT08-b* can complement a loss of *GmSHMT05*. We demonstrated that targeted knockout of *GmSHMT05* in Forrest and the Forrest *Gmshmt08-a* F234 null mutant impaired root growth, suggesting that *GmSHMT08-a* is unable to complement a loss of *GmSHMT05* in these soybean genotypes. This is consistent with GmSHMT08-a losing its normal enzymatic function in growth and development during domestication, acquiring a specific and potentially different activity that contributes to SCN resistance (Wu et al., 2016). That said, introduction of *GmSHMT08-a* and *GmSHMT08-b* transgenes into the same *Gmshmt05* null background would strengthen this claim. In contrast, targeted knockout of one functional cytosolic *SHMT* genes (either *GmSHMT05* or *GmSHMT08-b*) did not compromise root growth in the susceptible soybean cv. Essex, which supports a similar activity of these soybean cytosolic SHMTs, consistent with the aforementioned biochemical analysis. The weak interaction observed between GmSHMT05 and GmSHMT08 in yeast two-hybrid assays is consistent with the possibility that cytosolic SHMTs may form mixed oligomeric assemblies *in vivo*. If so, incorporation of the impaired GmSHMT08-a subunit into mixed oligomers could potentially exert a dominant-negative effect on cytosolic SHMT activity, although the extent and physiological relevance of such interactions remain unknown.

Our finding that *GmSHMT05* can complement the loss of function of *GmSHMT08-b* suggested a second copy of cytosolic *SHMT* might be dispensable for soybean growth and development, and therefore neofunctionalization would not come at a cost. However, in years past, soybean breeders noted yield drag in SCN-resistant soybeans in the absence of nematode pressure, and this was attributed to linkage of yield-suppressive factors with SCN resistance genes (Kopisch-Obuch et al., 2005; Donald et al., 2006; Chen and Potter, 2025). These reports, as well as the high level of expression of cytosolic *SHMT*s reported in pods and roots (Severin et al., 2010), which suggests a high demand for 1C folate metabolism in these tissues, led us to question whether the gain-of-function of *GmSHMT08-a* in SCN resistance comes with any expense to soybean growth and development.

Overall, field-grown *Gmshmt08-a* mutant plants did not exhibit any obvious aboveground phenotypes. However, we measured a significant increase in pod numbers/plant in the *Gmshmt08-a* mutants in the field environment and a numerical increase in pod numbers/plant in the greenhouse environment, consistent with the high level of expression of *GmSHMT08-a* in pods. We attribute the lack of significance in pods/plant in the greenhouse to the high level of variation we observed in this environment relative to the field. In yield trials, a significant interaction between genotype and location was detected for yield. Across locations, the F234 mutant produced numerically higher yield at three of four locations, while F1261 produced numerically higher yield at two of four locations. Both mutants showed a significant yield increase at the Portageville, MO location. Although maturity ratings were variable across tests, we consistently noted that *Gmshmt08-a* mutants exhibited delayed maturity (ranging from 1 to 5 days). This variation may be attributed to multiple individuals taking maturity ratings, coupled with the subjectivity of the rating system. Therefore, we cannot exclude the possibility that *GmSHMT08-a* is contributing to earlier maturity in Forrest, resulting in the observed decreased number of pods and seed relative to the mutants. In addition to an increase in pod number/plant, our root growth analyses revealed a significant increase in lateral root and total root length in *Gmshmt08-a* mutant plants, consistent with the high level of expression of *GmSHMT08-a* in roots. Collectively, these results indicate that *GmSHMT05* sustains overall soybean growth and development in the absence of *GmSHMT08*; however, the gain-of-function of *GmSHMT08-a* in SCN resistance negatively influences root growth and pod numbers, which may impact yield of resistant lines in the absence of SCN infestation. A similar scenario was previously reported in *Nicotiana attenuata*, where the disruption of plant trypsin proteinase inhibitors (TPIs), involved in jasmonic acid-induced defense, led to an increase in the number of seed capsules. Conversely, when TPI was constitutively expressed to restore its production in a natural genotype unable to produce TPI, capsule numbers decreased (Zavala et al., 2004).

If the presence of *GmSHMT08-a* reduces yield, then elimination or replacement of *GmSHMT08-a* in elite backgrounds when unnecessary, could improve yield. However, this strategy would result in the loss of resistance to HG type 0 (Race 3) nematode populations. That said, given that *GmSHMT08-a* is not required for resistance against the now widespread SCN HG type 2.5.7 (Race 1) or HG type 1.2.5.7 (Race 2) virulent populations (Howland et al., 2018), these high-yielding cultivars can still be used when other combinations of resistance genes known to target virulent SCN are present (Usovsky et al., 2023; Gamage et al., 2026). In summary, our findings highlight a potential trade-off between growth and defense when breeding soybean varieties for SCN resistance and high yield.

## Materials and methods

### Plant materials development

‘Forrest’ (PI 548655) is a maturity group (MG) V soybean cultivar released in 1973 by the USDA and Mississippi Agricultural and Forestry Experiment Station (Hartwig and Epps, 1973). It provides high resistance to SCN HG type 0 (race 3), which is bigenic, mediated by *rhg1-a* (*GmSNAP18-a*) and *Rhg4* (*GmSHMT08-a*) (Meksem et al., 2001). Previously, Forrest was used to develop an ethyl methanesulfonate (EMS)-mutagenized M2 population (Cooper et al., 2008; Meksem et al., 2008). Screening of this M2 population with SCN HG type 0 identified an allelic series of Forrest *Gmshmt08-a* mutants exhibiting increased susceptibility to SCN (Kandoth et al., 2017). Two Forrest *Gmshmt08-a* mutants, F1261 and F234, had nonsense mutations in *GmSHMT08-a* at amino acid positions 30 (Q30*) and 226 (Q226*), respectively (Kandoth et al., 2017). Each mutant was backcrossed (BC) to Forrest. The resulting BC_1_F_1_ plants were selfed to generate BC_1_F_2_ plants, of which 12 were screened for SCN HG type 0 resistance. Plants were also genotyped to confirm the mutations in *GmSHMT08-a* using primers listed in **Supplementary Table 13**. Plants exhibiting SCN female indices > 60% and homozygous for the mutations were selected for the second backcross with Forrest. The same procedure was conducted to produce BC_2_F_2_ plants susceptible to SCN HG type 0 and homozygous for the mutation in *GmSHMT08-a*. Both F234 and F1261 plants homozygous for *Gmshmt08-a* mutations were then genotyped using a SoySNP50K BeadChip array (Song et al., 2013) at the USDA-ARS Beltsville Agricultural Research Center. The hybridization, washing, and scanning procedures were conducted according to the manufacturer’s instructions (Illumina Inc., San Diego, CA). SNPs were filtered for missing data, and genotypes were called using GenomeStudio v2.0 (Illumina Inc., San Diego, CA). To confirm that the mutants were derived from Forrest, SNP genotypes were imported into Flapjack (version 1.22.04.01) (Milne et al., 2010).

### Nematode inoculum preparation

SCN (*Heterodera glycin*es Ichinohe) inbred population PA3 (HG type 0) was maintained on the susceptible soybean cultivar Williams 82 at the University of Georgia Plant Nematology Lab. Cysts from infested soil were extracted and collected on a no. 60-mesh (250-µm) sieve, gently crushed using a drill press, and the eggs collected on a no. 500-mesh (25-µm) sieve. The eggs were purified by sucrose centrifugation flotation and resuspended in water at a concentration of 1,000 eggs/mL.

### Phenotyping Forrest and *Gmshmt08-a* mutants against SCN HG type 0 population

Seed of Forrest, F234, F1261, and Lee 74 (SCN-susceptible check) were rolled in germination paper and incubated for three days in an incubator at 28°C. Three-day-old uniform seedlings were transplanted into thin-walled polyvinyl carbonate (PVC) tubes (15.24 cm long, 3 cm inside diameter) filled with a mixture of sterile sand and soil in a 1:1 ratio. These PVC tubes were arranged in closed-bottom plastic containers, or crocks, in a completely randomized design. Each seedling was inoculated with 1,000 eggs/mL inoculum. Crocks with plants were kept in the greenhouse suspended in a temperature-controlled water table set at 27°C for 28 days. At 28 days, females were extracted from roots and counted under a stereoscope. The SCN female index (FI) was calculated as the number of females on the soybean genotype of interest divided by the number of females on the susceptible check Lee 74 and multiplied by 100 to convert to a percentage.

### Plant phenotyping in the field and yield trials

Field phenotyping was conducted at the University of Georgia Iron Horse Farm (IHF) near Athens, GA, during the 2023 growing season. The genotypes Forrest, F234, and F1261 were planted with three replications in a randomized complete block design (RCBD). Each plot consisted of four rows with a row length of 1.83 m and 0.76 m row spacing. Seeds were planted at a planting density of approximately 33 seeds m^−1^. Days from seed planting to 50% of plants flowering in a plot were visually noted to determine the flowering time (FT). The maturity (MAT) was determined as the number of days when 95% of the plants in a plot reached maturity (R8 stage) (Fehr and Caviness, 1977). Ten plants were randomly collected across the two middle rows in a plot (five plants per row), while avoiding the outermost plants, to measure plant height and number of pods and seeds per plant, and these measurements were then averaged per plot. Seed from these 10 plants per plot was bulked to measure 100-seed weight and seed composition. Seed composition was analyzed using the near-infrared Perten DA 7250 Analyzer (PerkinElmer Inc., Waltham, MA, USA) and reported on a dry matter basis. Due to the shorter row length, plot yield from this experiment was not considered.

Yield trials for Forrest, F234, and F1261 were conducted during the summer of 2025 at four locations: Athens, GA; Plains, GA; Fisk, MO; and Portageville, MO. The experiment was arranged in an RCBD with three replications per location. In Athens and Plains, GA, each plot consisted of four rows with 76.2-cm row spacing and 4.9-m row length. Seeds were planted at a density of 27 seeds m^−1^. Before harvest, 122 cm was trimmed from both ends of each plot. The remaining 3.7 m of the two middle rows in each plot was harvested using a plot combine. In Fisk, MO, and Portageville, MO, plots were established in the same manner as those in Athens, GA, except that the plots with a length of 3.7 m were planted and harvested using a plot combine. At all locations, two center rows of each plot were also used for agronomic data collection and seed composition analysis. Maturity was determined as the number of days past 31 August for 95% of plants in a plot that reached maturity (R8). Plant height was measured as the average height, in centimeters, of three plants randomly selected from the middle two rows, from the ground to the top of the plant. Lodging was measured on a scale of 1 to 5, where 1 indicates all plants in a plot growing upright and 5 indicates plants fully lodged on the ground. Seed size was determined as 100-seed weight (g). Plot yield was adjusted to a 13% moisture basis and reported in kg ha^−1^.

### Greenhouse phenotyping

Greenhouse phenotyping for Forrest, F234, and F1261 was performed in the University of Georgia Athens greenhouse facility from January to May 2025. The experiment was arranged in an RCBD with three pots per genotype in each of three replications, and pots were spaced approximately 45 cm apart on a bench. Three seeds for each genotype were planted in an 11.4 L pot containing potting mix pretreated with Marathon^®^ 1% G (imidacloprid). After 10 days, seedlings were thinned to one plant per pot. Osmocote 14–14–14 (20–22 g) was applied at 9 days after planting, followed by weekly fertilization from 20 days using Southern Ag 20–20–20 (5.6 g/L) and Jackpot micronutrients (3.74 g/L; 400 mL per plant). Plants were watered twice daily using an automated irrigation system (~800 mL until flowering, then ~1,200 mL). A 16/8 h light/dark photoperiod was maintained for 30 days using supplemental high-pressure sodium (HPS, 400 W bulbs) lighting, then shifted to 12 h/12 h light/dark. Day/night temperatures were maintained at 28/21 °C with 70%–80% relative humidity. Plants were supported with bamboo stakes during growth. FT, MAT, plant height, number of pods, number of seeds, 100-seed weight, and seed composition were measured for each individual plant.

### Root phenotyping

Seeds of Forrest, F234, and F1261 were germinated in pouches at 28 °C for 4 days. Uniform seedlings were transferred individually to larger pouches (34 cm × 13 cm, Mega International, Minneapolis, MN, USA) trimmed at the base for water wicking and placed in black plastic bags (two pouches per bag) to maintain darkness. Bags were positioned in plastic containers (32 cm × 26 cm × 9.8 cm) containing 2.5 L water and maintained in a growth chamber at 26 °C under a 16/8 h light/dark cycle with ~200 µmol m^-2^ s^-1^ light intensity. Four biological replicates, each with three plants (12 total), were analyzed. Root images were captured at 10 days post-germination, and primary, lateral, and basal root lengths were measured using ImageJ (v1.46r). For root dry weight, 4-day-old seedlings were grown in soil-filled cones for 8 days. Roots were harvested, washed, oven-dried at 70 °C for 4 days, and weighed.

### Statistical analysis for field data and root phenotyping

Statistical analyses of the data were performed in R (v4.4.1). Field and greenhouse phenotyping data were analyzed using analysis of variance (ANOVA), followed by mean separation with the least significant difference (LSD) *post hoc* test (p <0.05). Yield trial data were analyzed using ANOVA to test the effects of genotype (G), location (L), G × L, and replications (R) within location. When G × L was significant, effects were then analyzed to compare genotypes within each location. For unbalanced data, harmonic LSD based on the harmonic mean of replicates was used for conservative mean separation.

### Protein expression and purification

The soybean *GmSHMT05* (NP_001355693.1) and *GmSHMT08-b* (NP_001355691.1) genes were commercially synthesized (GenScript) with codon optimization for expression in *Escherichia coli* and inserted into the pET14b vector with an N-terminal His_6_-affinity tag and a tobacco etch virus protease site. Expression plasmids were transformed into *E. coli* BL21(DE3) pLysS cells. Transformed *E. coli* cells were added to 10 mL of Luria broth medium containing 100 mg/mL ampicillin and 50 μg L^−1^ chloramphenicol, grown overnight at 37 °C, and diluted with 1 L of terrific broth (ratio 1:100). Cultures were incubated with shaking at 250 rpm until the *A*_600_ reached 0.7–0.8. After cooling to 19 °C, isopropyl-β-D-thiogalactopyranoside was added to a final concentration of 0.5 mM to induce expression. The culture was maintained for 18 h at 19 °C before harvesting by centrifugation at 7,000 rpm for 30 min at 4 °C.

For protein purification, cells were suspended in a lysis buffer [50 mM HEPES (pH 7.5), 20 mM imidazole, 500 mM NaCl, 10% (v/v) glycerol, 1% Tween 20, and 1 mM pyridoxal-5’-phosphate] and disrupted by ultrasonication in an ice bath. Cell debris was removed by centrifugation at 16,500 rpm for 1 h at 4 °C. The resulting supernatant was filtered through a 0.45 µm syringe-driven filter and loaded onto a nickel-nitrilotriacetic acid affinity column. The column was washed with 10 column volumes of 50 mM HEPES, pH 7.5, 500 mM NaCl, 20 mM imidazole, and 10% (v/v) glycerol. The protein was eluted with 50 mM HEPES, pH 7.5, 500 mM NaCl, 250 mM imidazole, and 10% (v/v) glycerol. Eluate was dialyzed using a 10-kDa-cutoff Slide-A-Lyzer cassette against [20 mM HEPES (pH 7.5), 150 mM NaCl, 0.05 mM Tris(carboxyethyl)phosphine (TCEP), and 5% glycerol] at 4 °C overnight. Flow-through, wash, and eluate fractions were analyzed for purity by SDS-PAGE. Protein-containing fractions were pooled, concentrated, and then flash-frozen in liquid nitrogen and stored at −80 °C.

### Size exclusion chromatography

A molecular weight standard kit, including bovine serum albumin, cytochrome c, carbonic anhydrase, and human DOPA decarboxylase (Cytiva), was used to calibrate the Superdex 200 10/300 GL (Cytiva). Samples of 100 µM GmSHMT05 or GmSHMT08-b (obtained after centrifugation at 10,000 rpm for 10 min) were loaded into the Superdex 200 10/300 GL and eluted at 0.5 mL/min using a buffer of 50 mM HEPES (pH 7.5), 150 mM NaCl, 0.5 mM TCEP, and 5% glycerol as the mobile phase. The estimated molecular weight of GmSHMT05 and GmSHMT08-b from size exclusion chromatography is 216 kDa, essentially equivalent to the calculated value for the tetramers (218 kDa).

### Crystallization

For crystallization experiments, GmSHMT05 samples were diluted to 12 mg mL^−1^ using a buffer containing 25 mM HEPES (pH 7.5), 50 mM NaCl, and 0.5 mM TCEP. All crystallization experiments were performed by hanging-drop vapor diffusion with a 1:1 protein:reservoir ratio. Initial crystals of GmSHMT05 were obtained in Index Screen condition 62 (Hampton Research), which contains 0.2 M trimethylamine N-oxide hydrate, 0.1 M Tris (pH 8.5), and 20% (w/v) PEG monomethyl ether 2000. Optimization screens were carried out using an Oryx8 crystallization robot (Douglas Instruments). To form GmSHMT05 ligand complexes with either PLP-Gly or PLP-Gly plus 5-formyl-tetrahydrofolate (FTHF), stock solutions of ligands were prepared in a buffer containing 25 mM HEPES (pH 7.5), 50 mM NaCl, and 0.5 mM TCEP. Cocrystallization experiments used ligand concentrations of 20 mM glycine and 10 mM FTHF in both the protein and reservoir solution. Crystals of GmSHMT05 in the reported structures were obtained in 0.08 M–0.22 M trimethylamine N-oxide hydrate, 0.1 M Tris (pH 8.5), and 20–26% (w/v) PEG monomethyl ether 2000. Crystals were cryoprotected in the reservoir solution supplemented with 15% (v/v) ethylene glycol and flash-cooled in liquid nitrogen.

### X-ray diffraction data collection, phasing, and refinement

X-ray diffraction data were collected on Advanced Light Source Beamline 4.2.2 using a Taurus-1 CMOS detector and Beamline 5.0.2 using a Pilatus 3 6M detector in shutterless mode. The data sets consisted of 900 images spanning 180°. The data were integrated and scaled with XDS (Kabsch, 2010), and intensities were merged and converted to amplitudes with Aimless (Evans and Murshudov, 2013). Initial phases for the GmSHMT05 complex with PLP-Gly and FTHF, and the GmSHMT05 complex with PLP-Gly, were determined by molecular replacement in Phaser (Zwart et al., 2008) using the structures of GmSHMT08-b complexed with PLP-glycine and FTHF [Protein Data Bank (PDB) ID 6UXJ] and PLP-glycine (PDB ID 6UXI) as templates, respectively (Korasick et al., 2020). The model was improved using the PHENIX software package (Adams et al., 2010) via iterative rounds of refinement with phenix.refine (Afonine et al., 2012) and manual building in COOT (Emsley and Cowtan, 2004). The structures were validated using MolProbity (Chen et al., 2010) and the wwPDB validation service (Gore et al., 2017). Data collection and refinement statistics are in **Supplementary Table 2**.

### Determination of specific activities for the retro-aldol cleavage reaction

To determine and compare the specific activities for the folate-independent retro-aldol cleavage, DL-phenylserine (Millipore-Sigma) was used as a substrate. The reactions were carried out in a buffer containing 25 mM HEPES (pH 7.5), 50 mM NaCl, and 0.5 mM TCEP in an Epoch II plate reader (BioTek). Each reaction contained 1 µM GmSHMT05 or GmSHMT08-b and 1 mM–250 mM DL-phenylserine. Benzaldehyde formation was monitored at 279 nm. The initial slopes of reactions were used to determine enzyme activity, and the calculation utilized a molar extinction coefficient of 1,400 M^−1^ cm^−1^ for benzaldehyde (Chiba et al., 2012). The results were graphed and analyzed in Origin 2024. Errors reported are standard error of the mean (SEM), where SEM = s/√n, s is standard deviation and n is number of observations.

### Steady-state kinetics for the SHMT two-substrate reaction

Steady-state kinetic parameters for the SHMT reaction in which L-serine and THF are converted to glycine and 5,10-MTHF were determined using the previously reported coupled reaction utilizing the NADP^+^-dependent enzyme 5,10-methylenetetrahydrofolate dehydrogenase (MTHFD) (Sopitthummakhun et al., 2012; Korasick et al., 2024). EcMTHFD was overexpressed and purified from EcMTHFD; FolD as previously reported (Sah and Varshney, 2015; Korasick et al., 2020). MTHFD-coupled assay studies were carried out in an Epoch II plate reader (BioTek) in a reaction buffer containing 50 mM HEPES (pH 7.5) and 0.5 mM EDTA. The reactions contained either 0.025 µM Essex SHMT8 or 0.025 µM SHMT5. NADPH production was continuously monitored at 375 nm. Assays to determine the apparent Michaelis constant of THF contained 1 mM NADP^+^, 5 µM EcMTHFD, 2 mM L-serine, and a concentration series of THF ranging from 0.003 mM to 0.4 mM. Reported kinetic parameters for either Essex SHMT8 or SHMT5 with THF as the variable substrate were determined by fitting the average of two technical replicates to a Michael–Menten model. Assays to determine the apparent Michaelis constant of L-serine contained 1 mM NADP^+^, 5 µM EcMTHFD, 1 mM THF, and a concentration series of L-serine ranging from 0.04 mM to 5 mM. Kinetic parameters with serine as the variable substrate were determined by fitting a Michaelis–Menten model to the average of two independent replicates. The initial slopes of the reactions were used to determine enzyme activity, and the calculation utilized a molar extinction coefficient of 1,920 M^−1^cm^−1^ for NADPH at 375 nm (Pinthong et al., 2014). The results were graphed and analyzed in Origin 2024b. The error bars in **Supplementary Figures 3D, E** are the SD values calculated from technical replicates. The uncertainties reported in the text and **Supplementary Table 2** are the standard errors from curve fitting with Origin 2024b.

### Folate-binding assays

Folate-binding assays were performed as previously reported (Owuocha et al., 2024) with minor modifications. Enzyme solutions (20 µM) were prepared and mixed in a ratio of 1:1 with a solution of either glycine or FTHF, respectively, in a 96-well plate. The working concentrations of glycine tested were 0.5 mM–20 mM. The working concentrations of FTHF tested were 10 µM–100 µM. The plate was analyzed by absorbance scan (450 nm–550 nm) in a BioTek Epoch II plate reader, following the formation of a peak at 500 nm, consistent with the formation of the quinonoid intermediate and representative of the total ternary complex in the enzyme solution (Strong et al., 1989). The absorbance at 500 nm was baseline subtracted from the absorbance at 540 nm. The data were analyzed by plotting absorbance versus substrate concentration and were fit to a one-site saturation binding model using the Michaelis–Menten equation, from which apparent *K*_d_ values were derived. Data were plotted and analyzed in Origin 2024.

### Yeast-two hybrid

Protein–protein interactions were examined using a GAL4-based yeast two-hybrid system (Creative Biolabs, NY, USA). Full-length coding sequences of SHMT05 and SHMT08 were synthesized and cloned into pGBKT7 (BD) and pGADT7 (AD) vectors, respectively, and sequence-verified. Bait and prey constructs were co-transformed into *Saccharomyces cerevisiae* Y2HGold. Bait toxicity and autoactivation were tested using empty pGADT7. pGBKT7-53/pGADT7-T and pGBKT7-Lam/pGADT7-T were used as positive and negative controls. Transformants were selected on SD/-Leu/-Trp and screened on SD/-Leu/-Trp/-His/-Ade containing X-α-Gal, with interactions scored by growth and blue coloration.

### Design and construction of CRISPR/Cas9 constructs

A dual single guide (sgRNA) plasmid construction system was used to target *GmSHMT05* and *GmSHMT08* for editing, as described in Kang (2016). Several gRNAs were designed targeting *GmSHMT05*, as well as for the simultaneous editing of *GmSHMT05* and *GmSHMT08*, using the CHOPCHOP webtool (Labun et al., 2016). The double-stranded sgRNA sequences were cloned into the AtU6-26-SK vector. Next, both the Cas9 cassette from 35S-Cas9-SK and two sgRNA expression cassettes were digested and cloned into the pCamGFP-CvMV-GWOX binary vector, which includes a GFP reporter gene driven by the CvMV promoter. This construct was introduced into the *Rhizobium rhizogenes* K599 strain using a freeze-thaw method (Höfgen and Willmitzer, 1988). Several dual guides were constructed to target only *GmSHMT05* or to target both *GmSHMT05* and *GmSHMT08* simultaneously. The effectiveness of dual guide systems was confirmed through sequencing of targeted genes in K599-inoculated plants in preliminary experiments. Based on these results, pcamGFP-CvMV-*GmSHMT05*-T3+T5 (CRISPR/Cas9-*GmSHMT05*-T3+T5), exclusively targeting *GmSHMT05*, and pcamGFP-CvMV-*GmSHMT05&08*-T1+T2 (CRISPR/Cas9-*GmSHMT05&08*-T1+T2), targeting both *GmSHMT05* and *GmSHMT08*, were selected as final constructs. The pcam-GFP-CvMV-GWOX construct without sgRNA was used as the empty vector (EV) control. The dual guide pcamGFP-CvMV-*GmSHMT08*-T1+T4 (CRISPR/Cas9-*GmSHMT08*-T1+T4) to target *GmSHMT08* was described previously in Kang (2016). Primers used for vector construction are listed in **Supplementary Table 13**.

### Generation of composite soybean plants

Composite soybean plants treated with K599 carrying CRISPR/Cas9-*GmSHMT05*-T3+T5, CRISPR/Cas9-*GmSHMT08*-T1+T4, and CRISPR/Cas9-*GmSHMT05&08*-T1+T2 were generated as previously described (Fan et al., 2020; Gamage et al., 2025). Explants with at least one root showing GFP expression were considered transformed. The percentage of transformed explants was calculated as the proportion of plants with GFP (+) roots relative to the number of plants inoculated with *R. rhizogenes* K599. The percentage of GFP (+) roots was calculated as (number of plants with GFP (+) roots when plants were infected with CRISPR/Cas9-EV/number of plants inoculated with K599) ×100 for the control. CRISPR/Cas9-*GmSHMT05*-T3+T5 or CRISPR/Cas9-*GmSHMT08*-T1+T4 or CRISPR/Cas9-*GmSHMT05&08*-T1+T2 number of plants inoculated with K599) ×100 as treatment. A two-tailed Student’s *t*-test was used to calculate the significant difference between the control and the treatments. Essex plants with GFP (+) roots were subjected to SCN bioassays in the greenhouse to assess the impact of *GmSHMT05* and *GmSHMT08* on nematode reproduction.

### RNA and DNA extraction, PCR, and sequencing

Total RNA was isolated from roots of 6-day-old Forrest, F234, and F1261 seedlings using the RNeasy Plant Mini kit (Qiagen), following the manufacturer’s protocol. Total RNA was used to synthesize cDNA using the PrimeScript 1st Strand cDNA Synthesis Kit (Takara). Full-length transcripts were amplified with *GmSHMT08-a*-specific primers using Q5^®^ High-Fidelity 2X Master Mix (NEB) in a Bio-Rad C1000 touch thermocycler. The cycling parameters were as follows: 30 s at 98 °C; 34 cycles of 10 s at 98 °C, 30 s at 60 °C, and 1.5 min at 72 °C; with a final extension at 72 °C for 2 min. The amplified PCR products were separated by 1% agarose gel electrophoresis.

Genomic DNA was extracted from root tissues using the CTAB method (Clarke, 2009) to confirm edits in *GmSHMT05* and *GmSHMT08*. Primer pairs F1/R1 and F2/R2 were used to amplify regions flanking the T3/T5 and T1/T4 sites in *GmSHMT05* and *GmSHMT08*, respectively. To confirm simultaneous editing of *GmSHMT05* and *GmSHMT08*, F1/R1 were used for *GmSHMT05*, while F3/R3 were used for *GmSHMT08* to amplify the region flanking the T1 and T2 target sites. PCR was performed using Q5^®^ High-Fidelity 2X Master Mix (NEB) on a Bio-Rad C1000 Touch thermocycler. The cycling parameters were as follows: 30 s at 98 °C; 34 cycles of 10 s at 98 °C, 30 s at 57 °C, and 1.5 min at 72 °C; with a final extension at 72 °C for 2 min. Amplicons were resolved on a 1% agarose gel; selected bands were purified (QIAquick^®^ Gel Extraction Kit, Qiagen) and Sanger sequenced using amplification primers.

## Data Availability

The original contributions presented in the study are included in the article/**Supplementary Material**. Further inquiries can be directed to the corresponding authors.
