# Supplementary material for: Functional divergence of two soybean cytosolic serine hydroxymethyltransferases in development and defense against soybean cyst nematode
Source: Front Plant Sci. 2026 Aug 31;17:1942553. doi: 10.3389/fpls.2026.1942553 (PMC13563002; doi:10.3389/fpls.2026.1942553)
Supplement: Supplementary file 1 [file DataSheet1.pdf]

Supplementary Table S1. X-ray diffraction and data collection statistics

Ligand Complex	PLP-Gly	PLP-Gly + FTHF
Space group	P 3_1 2 1	P 2_1
Unit cell parameters (Å, °)	a = 174.8, b = 174.8, c = 184.1<br>a = b = 90, g = 120	a = 60.7, b = 131.8, c = 117.4<br>b = 91.0
Beamline	ALS 4.2.2	ALS 4.2.2
Wavelength (Å)	1	1
Resolution (Å) [a]	48.67 - 1.90 (1.90 - 1.93)	41.17 - 1.96 (2.03 - 1.96)
Total reflections [a]	2537151 (120849)	462851 (18437)
Unique reflections [a]	254040 (12446)	130817 (6354)
Multiplicity [a]	10.0 (9.7)	3.5 (2.9)
Completeness (%) [a]	100.0 (100.0)	99 (98.1)
Mean I/s [a]	14.1 (1.7)	9.3 (1.4)
Wilson B-factor (Å^2)	27.48	28.89
R_{merge}(I) [a]	0.111 (1.648)	0.076 (0.736)
R_{meas}(I) [a]	0.118 (1.741)	0.089 (0.902)
R_{pim}(I) [a]	0.037 (0.557)	0.047 (0.512)
$CC_{1/2}$ [a]	0.999 (0.754)	0.997 (0.707)
R_{cryst} [a]	0.1964 (0.3317)	0.1757 (0.2512)
R_{free} [a, b]	0.2288 (0.3708)	0.2257 (0.3027)
Number of non-H atoms	22924	16250
macromolecules	21381	14569
ligands	132	376
solvent	1411	1305
Protein residues	2786	1884
rmsd bonds (Å)	0.008	0.008
rmsd angles (°)	0.97	0.99
Ramachandran favored (%)	97.27	97.07
Ramachandran allowed (%)	2.58	2.93
Ramachandran outliers (%)	0.15	0
Clashscore [c]	4.91	0.66
Average B-factor (Å^2)	36.64	6.3
macromolecules	36.47	33.79
ligands	37.06	33.51
solvent	39.2	30.71
PDB code	9DLT	9DHB

[a] Values for the outer resolution shell of data are given in parenthesis.
[b] 5% test set for refinement.
[c] Values from MolProbity.
Abbreviations: PLP-Gly, PLP-glycine external aldimine; FTHF, 5-formyl tetrahydrofolate

Supplementary Table S2. Steady-state kinetic and folate-binding parameters.

	GmSHMT05	GmSHMT08-b
Steady-state kinetic parameters: MTHFD-coupled reaction		
$k_{cat,\ THF}$ (s^{-1})	3 ± 0.4	2.7 ± 0.2
$K_{m,\ THF}$ (μM)	135 ± 39	91 ± 22
$k_{cat}/K_{m\ THF}$ ($mM^{-1}s^{-1}$)	23 ± 10	30 ± 9
$k_{cat,\ Ser}$(s^{-1})	9 ± 1.6	8 ± 0.5
$K_{m,\ Ser}$ (μM)	123 ± 58	115 ± 16
$k_{cat}/K_{m,\ Ser}$($mM^{-1}s^{-1}$)	73 ± 28	70 ± 31
Steady-state kinetic parameters: Retro-aldol cleavage of L-phenylserine		
k_{cat} (s^{-1})	1.5 ± 0.1	1.0 ± 0.04
K_m (mM)	85 ± 13	104 ± 7.6
k_{cat}/K$_m$($M^{-1}s^{-1}$)	18 ± 4.6	10 ± 2.4
Spectrophotometric FTHF-binding		
$K_{d,\ FTHF\text{-}glycine}$ (μM)‡	23	17*

Reported uncertainties are standard errors as calculated from curve fitting in Origin 2024b. ‡K_d determined from double reciprocal plot of data in Supplemental Figure S3, as described in (Korasick et al. 2020). Value determined by this method corresponds to the K_d from the highest concentration of glycine.

*Data previously published (Korasick et al. 2020).

Supplemental Table S3. Comparisons of major phenotypic traits among Forrest, F234 and F1261 from field phenotyping. Mean comparison was done using ANOVA followed by Least Significant Difference (LSD) test at $p < 0.05$. Different letters indicate significant differences. All data were as mean ± s.d. '***' if $p < 0.001$, **' if $p < 0.01$, *' if $p < 0.05$.

Source of variation	Flowering time[†]	Maturity[†]	Plant height	Number of pods/plant[£]	Pods per plant by seed number			Number of seeds/plant[£]	Seed Size	Protein content[‡]	Oil content[‡]
	Days	Days	cm		1 seed	2 seeds	3 seeds		g/100-seed	(%)	(%)
Forrest	48^{a}	119 ± 1^{a}	85.1 ± 4^{a}	108 ± 26^{a}	12 ± 8^{a}	54 ± 24^{a}	41 ± 16^{a}	244 ± 56^{a}	14.2 ± 0.4^{a}	41.1 ± 0.4^{a}	22.3 ± 0.5^{a}
F234	48 ± 1^{ab}	124 ± 2^{b}	86.9 ± 4^{a}	135 ± 14^{b}	20 ± 9^{b}	74 ± 29^{b}	41 ± 20^{a}	291 ± 32^{a}	13.9 ± 0.3^{a}	40.7 ± 0.5^{ab}	21.9 ± 0.3^{a}
F1261	49 ± 1^{b}	120 ± 2^{a}	82 ± 6.8^{a}	131 ± 19^{b}	14 ± 7^{a}	75 ± 39^{b}	42 ± 22^{a}	290 ± 46^{a}	15 ± 0.7^{b}	40.4 ± 0.7^{b}	22 ± 0.3^{a}
Genotype (G)	0.1	$1.8 \times 10^{-3**}$	0.2	$3.8 \times 10^{-2*}$	$1.1 \times 10^{-3**}$	$1.7 \times 10^{-2*}$	1	8.8×10^{-2}	$7.2 \times 10^{-3**}$	$4.6 \times 10^{-2*}$	7×10^{-2}
Block (B)	0.7	0.2	$3 \times 10^{-2*}$	6.7×10^{-2}	0.7	0.3	5.9×10^{-2}	$4.6 \times 10^{-2*}$	0.4	$2.6 \times 10^{-2*}$	5.5×10^{-2}
LSD	1	2.4	5.2	22	4	16	10	49	0.6	0.6	0.4

[†]Number of days since planting.
[£]Average number of pods and seeds per plant was calculated from 10 plants per plot (or block) per genotype. Therefore, the mean values were calculated from a total of 30 plants per genotype.
[‡]Protein and Oil (%) → Values are expressed on a dry matter basis.

Supplementary Table S4: Major amino acid composition of Forrest, F234 and F1261 seeds from field phenotyping. Mean comparison was done using ANOVA followed by Least Significant Difference (LSD) test at $p < 0.05$. Different letters indicate significant differences. All data were as mean ± s.d. '***' if $p < 0.001$, **' if $p < 0.01$, *' if $p < 0.05$. Values are expressed on a dry matter basis.

Source of variation	Lysine	Cysteine	Methionine	Threonine	Tryptophan	Isoleucine	Leucine	Histidine	Valine	Phenylalanine
					(%)					
Forrest	2.68 ± 0.03[a]	0.54 ± 0.01[a]	0.54 ± 0.00[a]	1.53 ± 0.01[a]	0.41 ± 0.01[a]	1.99 ± 0.02[a]	3.15 ± 0.04[a]	1.06 ± 0.01[a]	2.02 ± 0.03[a]	2.13 ± 0.03[a]
F234	2.65 ± 0.02[a]	0.54 ± 0.01[a]	0.54 ± 0.01[a]	1.51 ± 0.01[a]	0.41 ± 0.01[a]	1.96 ± 0.02[a]	3.11 ± 0.03[a]	1.04 ± 0.01[a]	1.99 ± 0.02[b]	2.10 ± 0.03[b]
F1261	2.65 ± 0.03[a]	0.55 ± 0.01[a]	0.54 ± 0.01[a]	1.51 ± 0.02[a]	0.41 ± 0.01[a]	1.96 ± 0.03[a]	3.10 ± 0.05[a]	1.04 ± 0.02[a]	1.99 ± 0.03[b]	2.10 ± 0.03[b]
Genotype (G)	0.18	0.62	0.72	0.09	0.95	0.09	0.09	0.11	0.06	$4.7 \times 10^{-2*}$
Block (B)	0.45	0.09	0.04	0.05	0.03	0.3	0.16	0.1	0.18	0.08
LSD	0.04	0.01	0.01	0.02	0.01	0.03	0.05	0.01	0.03	0.03

Supplementary Table S5. Important fatty acid content in Forrest, F234 and F1261 seeds from field phenotyping. Mean comparison was done using ANOVA followed by Least Significant Difference (LSD) test at $p < 0.05$. Different letters indicate significant differences. All data were as mean ± s.d. '***' if $p < 0.001$, **' if $p < 0.01$, *' if $p < 0.05$. Values are expressed on a dry matter basis.

Source of variation	Linoleic acid	Linolenic acid	Oleic acid
		(%)	
Forrest	48.9 ± 1.6[a]	6.8 ± 0.2[a]	28.1 ± 1.1[a]
F234	48.2 ± 1.5[a]	6.9 ± 0.4[a]	28.4 ± 0.7[a]
F1261	47.8 ± 1.3[a]	6.8 ± 0.4[a]	28.9 ± 0.9[a]
Genotype (G)	0.4	0.6	0.6
Block (B)	0.8	$1.6 \times 10^{-3**}$	0.5
LSD	1.8	0.3	1.2

Supplemental Table S6. Comparisons of major phenotypic traits among Forrest, F234 and F1261 from greenhouse phenotyping. Mean comparison was done using ANOVA followed by Least Significant Difference (LSD) test at $p < 0.05$. Different letters indicate significant differences. All data were as mean ± s.d. ‘***’ if $p < 0.001$, **’ if $p < 0.01$, *’ if $p < 0.05$.

Source of variation	Flowering time†	Maturity†	Plant height	Number of pods/plant£	Pods per plant by seed number				Number of seeds/plant£	Seed Size	Protein content‡	Oil content‡
	Days	Days	cm		1 seed	2 seeds	3 seeds	4 seeds		g/100-seed	(%)	(%)
Forrest	52 ± 1^{a}	107 ± 5^{a}	178.6 ± 24.2^{a}	276 ± 69^{a}	45 ± 17^{a}	140 ± 37^{a}	88 ± 30^{a}	3 ± 2^{a}	602 ± 152^{a}	10.6 ± 0.6^{a}	33.9 ± 1.3^{a}	24.9 ± 0.6^{a}
F234	52 ± 1^{a}	106 ± 5^{a}	188.6 ± 24.5^{a}	298 ± 33^{a}	64 ± 12^{ab}	141 ± 26^{a}	91 ± 18^{a}	2 ± 2^{a}	628 ± 71^{a}	10.8 ± 1^{a}	34.2 ± 1.1^{a}	24.7 ± 0.5^{a}
F1261	54 ± 2^{b}	108 ± 5^{a}	162.2 ± 41.6^{a}	295 ± 57^{a}	54 ± 11^{b}	154 ± 33^{a}	86 ± 24^{a}	2 ± 1^{a}	625 ± 129^{a}	10.9 ± 0.6^{a}	33.1 ± 1.1^{a}	24.5 ± 0.6^{a}
Genotype (G)	$2.8 \text{ x } 10^{-3**}$	0.7	0.2	0.7	$2.4 \text{ x } 10^{-2*}$	0.5	0.9	0.1	0.9	0.7	0.1	0.4
Block (B)	0.9	0.5	0.3	0.4	0.7	0.1	0.4	0.4	0.6	0.6	0.2	0.8
$LSD_{(0.05)}$	1.3	4.9	31.2	54	13	30	24	1.7	121	0.8	1.1	0.6

†Number of days since planting.
£Average number of pods and seeds per plant was calculated from total of 9 plants (3 plants per block) per genotype in greenhouse phenotyping.
‡Protein and Oil (%) → Values are expressed on a dry matter basis.

Supplementary Table S7: Major amino acid composition of Forrest, F234 and F1261 seeds from greenhouse phenotyping. Mean comparison was done using ANOVA followed by Least Significant Difference (LSD) test at $p < 0.05$. Different letters indicate significant differences. All data were as mean ± s.d. '***' if $p < 0.001$, **' if $p < 0.01$, *' if $p < 0.05$. Values are expressed on a dry matter basis.

Source of variation	Lysine	Cysteine	Methionine	Threonine	Tryptophan	Isoleucine	Leucine	Histidine	Valine	Phenylalanine
	Dry basis %									
Forrest	2.31 ± 0.06[a]	0.55 ± 0.02[a]	0.51 ± 0.01[a]	1.38 ± 0.03[a]	0.33 ± 0.01[a]	1.64 ± 0.06[a]	2.61 ± 0.1[a]	0.89 ± 0.03[a]	1.67 ± 0.05[a]	1.72 ± 0.07[a]
F234	2.32 ± 0.05[a]	0.56 ± 0.02[a]	0.51 ± 0.01[a]	1.39 ± 0.03[a]	0.34 ± 0.02[a]	1.65 ± 0.05[a]	2.63 ± 0.08[a]	0.9 ± 0.03[a]	1.68 ± 0.05[a]	1.73 ± 0.06[a]
F1261	2.28 ± 0.06[a]	0.55 ± 0.01[a]	0.50 ± 0.01[a]	1.36 ± 0.03[a]	0.34 ± 0.03[a]	1.60 ± 0.05[a]	2.55 ± 0.09[a]	0.88 ± 0.03[a]	1.64 ± 0.05[a]	1.68 ± 0.06[a]
Genotype (G)	0.22	0.22	0.13	0.26	0.79	0.15	0.19	0.51	0.16	0.23
Block (B)	0.21	0.76	0.60	0.27	0.78	0.21	0.27	0.18	0.32	0.19
LSD	0.06	0.02	0.01	0.03	0.02	0.05	0.09	0.03	0.05	0.06

Supplementary Table S8. Important fatty acid content in Forrest, F234 and F1261 seeds from greenhouse phenotyping. Mean comparison was done using ANOVA followed by LSD test at $p < 0.05$. Different letters indicate significant differences. All data were as mean ± s.d. '***' if $p < 0.001$, **' if $p < 0.01$, *' if $p < 0.05$. Values are expressed on a dry matter basis.

Source of variation	Linoleic acid	Linolenic acid	Oleic acid
		(%)	
Forrest	58.9 ± 2.7^a	8.8 ± 0.3^a	20.3 ± 2^a
F234	59 ± 1.4^a	8.9 ± 0.2^a	19.6 ± 1.2^a
F1261	55.6 ± 4^b	9.1 ± 0.4^a	22.4 ± 2.8^b
Genotype (G)	$3.1 \times 10^{-2*}$	0.1	$1.7 \times 10^{-2*}$
Block (B)	0.3	$2.1 \times 10^{-2*}$	$4.8 \times 10^{-2*}$
$LSD_{(0.05)}$	2.8	0.3	1.9

Supplementary Table S9: Major amino acid composition of Forrest, F234 and F1261 seeds from field trials. Mean comparison was done using ANOVA followed by Least Significant Difference (LSD) test at $p < 0.05$. Different letters indicate significant differences. All data were as mean ± s.d. '***' if $p < 0.001$, **' if $p < 0.01$, *' if $p < 0.05$. Values are expressed on a dry matter basis. Data not available for Fisk (MO).

Source of variation	Lysine	Cysteine	Methionine	Threonine	Tryptophan	Isoleucine	Leucine	Histidine	Valine	Phenylalanine
					(%)					
Genotype main effect										
Forrest	2.63 ± 0.07[a]	0.55 ± 0.03[a]	0.54 ± 0.01[a]	1.53 ± 0.03[a]	0.42 ± 0.03[a]	1.94 ± 0.06[a]	3.12 ± 0.09[a]	1.04 ± 0.04[a]	1.98 ± 0.06[a]	2.1 ± 0.06[a]
F234	2.61 ± 0.06[ab]	0.54 ± 0.03[a]	0.53 ± 0.01[a]	1.52 ± 0.03[a]	0.41 ± 0.02[b]	1.93 ± 0.05[a]	3.10 ± 0.07[a]	1.03 ± 0.03[a]	1.96 ± 0.05[a]	2.08 ± 0.05[ab]
F1261	2.57 ± 0.08[b]	0.55 ± 0.03[a]	0.53 ± 0.02[a]	1.5 ± 0.05[a]	0.41 ± 0.03[ab]	1.89 ± 0.06[b]	3.04 ± 0.10[b]	1.02 ± 0.04[a]	1.92 ± 0.07[b]	2.05 ± 0.07[b]
Location main effect										
Athens	2.55 ± 0.05[b]	0.53 ± 0.01[b]	0.52 ± 0.01[b]	1.48 ± 0.03[b]	0.39 ± 0.01[a]	1.9 ± 0.04[b]	3.03 ± 0.06[b]	1.01 ± 0.02[b]	1.93 ± 0.04[b]	2.04 ± 0.04[b]
Plains	2.67 ± 0.05[a]	0.54 ± 0.02[b]	0.54 ± 0.02[a]	1.54 ± 0.03[a]	0.41 ± 0.01[b]	1.98 ± 0.04[a]	3.19 ± 0.06[a]	1.07 ± 0.03[a]	2.02 ± 0.04[a]	2.15 ± 0.04[a]
Portageville	2.58 ± 0.03[b]	0.58 ± 0.01[a]	0.54 ± 0.01[a]	1.54 ± 0.02[a]	0.44 ± 0.02[c]	1.88 ± 0.02[b]	3.03 ± 0.03[b]	1.02 ± 0.01[b]	1.90 ± 0.03[c]	2.05 ± 0.02[b]
Genotype (G)	0.06	0.59	0.28	0.17	0.2	$7.63 \times 10^{-3**}$	$2.21 \times 10^{-2*}$	0.16	$5.77 \times 10^{-3**}$	$2.39 \times 10^{-2*}$
Location (L)	$2.22 \times 10^{-4***}$	$3.81 \times 10^{-4***}$	$1.11 \times 10^{-3**}$	$4.41 \times 10^{-3**}$	$1.41 \times 10^{-6***}$	$1.18 \times 10^{-5***}$	$3.49 \times 10^{-5***}$	$3.83 \times 10^{-4***}$	$1.54 \times 10^{-5***}$	$2.49 \times 10^{-5***}$
G x L	0.66	0.96	0.86	0.7	0.18	0.64	0.51	0.78	0.78	0.29
L(Block)	0.84	0.27	0.44	0.76	$2.45 \times 10^{-2*}$	0.73	0.89	0.9	0.59	0.83
$LSD_{G\ (0.05)}$, $LSD_{L\ (0.05)}$	0.05	0.02	0.01	0.03	0.01	0.03	0.05	0.03	0.03	0.03

Supplementary Table S10. Important fatty acid content in Forrest, F234 and F1261 seeds from field trials. Mean comparison was done using ANOVA followed by LSD test at $p < 0.05$. Different letters indicate significant differences. All data were as mean ± s.d. '***' if $p < 0.001$, **' if $p < 0.01$, *' if $p < 0.05$. Values are expressed on a dry matter basis.

Source of variation	Linoleic acid[d]	Linolenic acid[d]	Oleic acid[d]
		(%)	
Athens			
Forrest	-	8.1 ± 0.2[a]	-
F234	-	6.7 ± 0.5[b]	-
F1261	-	8.2 ± 0.4[a]	-
Plains			
Forrest	-	5.6 ± 0.3[a]	-
F234	-	5.7 ± 0.4[a]	-
F1261	-	6.7 ± 0.3[b]	-
Portageville			
Forrest	-	6.1 ± 1.1[a]	-
F234	-	6.2 ± 0.1[a]	-
F1261	-	6 ± 0.4[a]	-
Genotype Main Effect			
Forrest	51.2 ± 2.6[ab]	-	27.3 ± 3.1[a]
F234	52.2 ± 2.8[a]	-	26.6 ± 2.2[a]
F1261	50 ± 2.1[b]	-	28 ± 2.3[a]
Location Main Effect			
Athens	52.5 ± 2.2[a]	-	25.2 ± 1.7[a]
Plains	52 ± 2[a]	-	27.6 ± 2[b]
Portageville	48.4 ± 1.3[b]	-	29.5 ± 2[c]
Genotype (G)	0.1	8.3×10^{-3}**	0.4
Location (L)	3.7×10^{-4}***	4.1×10^{-6}***	7.9×10^{-4}***
G x L	0.5	1.1×10^{-2}*	0.3
L (Block)	0.2	0.9	0.1
$LSD_{G\ (0.05)}$, $LSD_{L\ (0.05)}$	1.6	0.8	1.7

Supplementary Table S11. Root phenotyping and statistical analysis of Forrest and Forrest *shmt08* mutants. Mean comparison was done using ANOVA followed by LSD test at $p < 0.05$. Different letters indicate significant differences. All data were as mean ± s.d. . '***' if $p < 0.001$, **' if $p < 0.01$, *' if $p < 0.05$.

Source of variation	Tap root length	Lateral root length	Total root length	Dry root weight
	cm			mg
Forrest	34.2 ± 9^{a}	269.2 ± 108.5^{a}	303.3 ± 112.9^{a}	43 ± 9.2^{a}
F234	33.6 ± 11.2^{a}	324.4 ± 106.5^{ab}	358.1 ± 107.5^{ab}	47.6 ± 10.9^{a}
F1261	34.8 ± 11.5^{a}	343.9 ± 128.6^{b}	378.7 ± 131.2^{b}	47.9 ± 16.5^{a}
Genotype (G)	0.9	$4.9 \times 10^{-2*}$	0.1	0.3
Replicate (R)	0.1	$6.4 \times 10^{-5***}$	$1.3 \times 10^{-4***}$	0.1
G x R	0.3	0.8	0.8	0.1
$LSD_{G\ (0.05)}$	6.5	62.7	76.3	7.2

Supplementary Table S12. Number of GFP (+) roots recovered – three replicates. Roots longer than 1 cm were selected for percentage calculation. Percentage was calculated as (Number of plants with GFP (+) roots/ Total number of plants inoculated with K599) * 100.

Replicate	Genotype	Construct	Total # of plants inoculated	Number of plants with GFP (+) roots	Percentage (%)
R1	Forrest	EV	14	11	78.57
		GmSHMT05 T5+T3	14	4	28.57
	F234	EV	14	13	92.86
		GmSHMT05 T5+T3	14	0	0.00
	Essex	EV	14	14	100.00
		GmSHMT05 T5+T3	14	13	92.86
		GmSHMT08 T1+T4	14	14	100.00
		GmSHMT05&08 T1+T2	14	8	57.14
R2	Forrest	EV	14	7	50.00
		GmSHMT05 T5+T3	14	2	14.29
	F234	EV	14	7	50.00
		GmSHMT05 T5+T3	14	2	14.29
	Essex	EV	14	14	100.00
		GmSHMT05 T5+T3	14	7	50.00
		GmSHMT08 T1+T4	14	13	92.86
		GmSHMT05&08 T1+T2	14	6	42.86
R3	Forrest	EV	14	12	85.71
		GmSHMT05 T5+T3	14	6	42.86
	F234	EV	14	12	85.71
		GmSHMT05 T5+T3	14	4	28.57
	Essex	EV	14	14	100.00
		GmSHMT05 T5+T3	14	13	92.86
		GmSHMT08 T1+T4	14	14	100.00
		GmSHMT05&08 T1+T2	14	12	85.71

Supplementary Table S13. Primers used for CRISPR and deletion check

Purpose	Primer name	Primer sequence 5'-3'
Primers to generate gRNA fragments to knockout *GmSHMT05*	T3-F	GATTGTGAGCTCGATGCCGCGGCAT
	T3-R	AAACATGCCGCGGCATCGAGCTCAC
	T5-F	GATTGGAAATTGCGGATAAGTGCG
	T5-R	AAACCGCACTTATCCGCAATTTCC
Primers to generate gRNA fragments to knock out both *GmSHMT05* & *GmSHMT08* simultaneously	T1-F	GATTGGGGCTGTTCACTTCCTGCG
	T1-R	AAACCGCAGGAAGTGAACAGCCCC
	T2-F	GATTGCACGGCTACTACACCTCCGG
	T2-R	AAACCCGGAGGTGTAGTAGCCGTGC
Primers to generate gRNA fragments to knockout *GmSHMT08*	T1-F	GATTGAGGGAAGTCGCTGATAAGTG
	T1-R	AAACCACTTATCAGCGACTTCCCTC
	T4-F	GATTGCTTGCGGGGCCCACGTGCG
	T4-R	AAACCGCACGTGGGCCCCGCAAGC
Primers for sequencing the Atu6-gDNA constructs in AtU6-26SK	M13R	CACAGGAAACAGCTATGAC
	SS42 (sequencing)	TCCCAGGATTAGAATGATTAGG
Primers to check final vector construct by PCR	SS42	TCCCAGGATTAGAATGATTAGG
	Cas9 - F	TCCCAGGATTAGAATGATTAGG
	Cas9 - R	CAGTTCGCCGGCAGAGGCCAGC
To check deletions in *GmSHMT05*	F1	ACCTCAAAGCAACACAAGAACA
	R1	TGAGATCTTGTTTTGAATTGAGTT
To check deletions in *GmSHMT08*	F2	TGGGGGCTAGATCTCCG
	R2	CTGCAGCGAAGGGAACAC
	F3	CTTCACTCACTCTCATTCATTC
	R3	CAAATCAAAACCAGAAGGTAGA
For GmSHMT08-a genotyping in backcrosses and cDNA amplification	F4	ACAACACTCTCTCTTCTCGC
	R4	CCGGAAGCTTCTATCCTTGTACTTCATTTCAG
