# Supplementary material for: Functional divergence of two soybean cytosolic serine hydroxymethyltransferases in development and defense against soybean cyst nematode
Source: Front Plant Sci. 2026 Aug 31;17:1942553. doi: 10.3389/fpls.2026.1942553 (PMC13563002; doi:10.3389/fpls.2026.1942553)
Supplement: Supplementary file 2 [file DataSheet2.pdf]

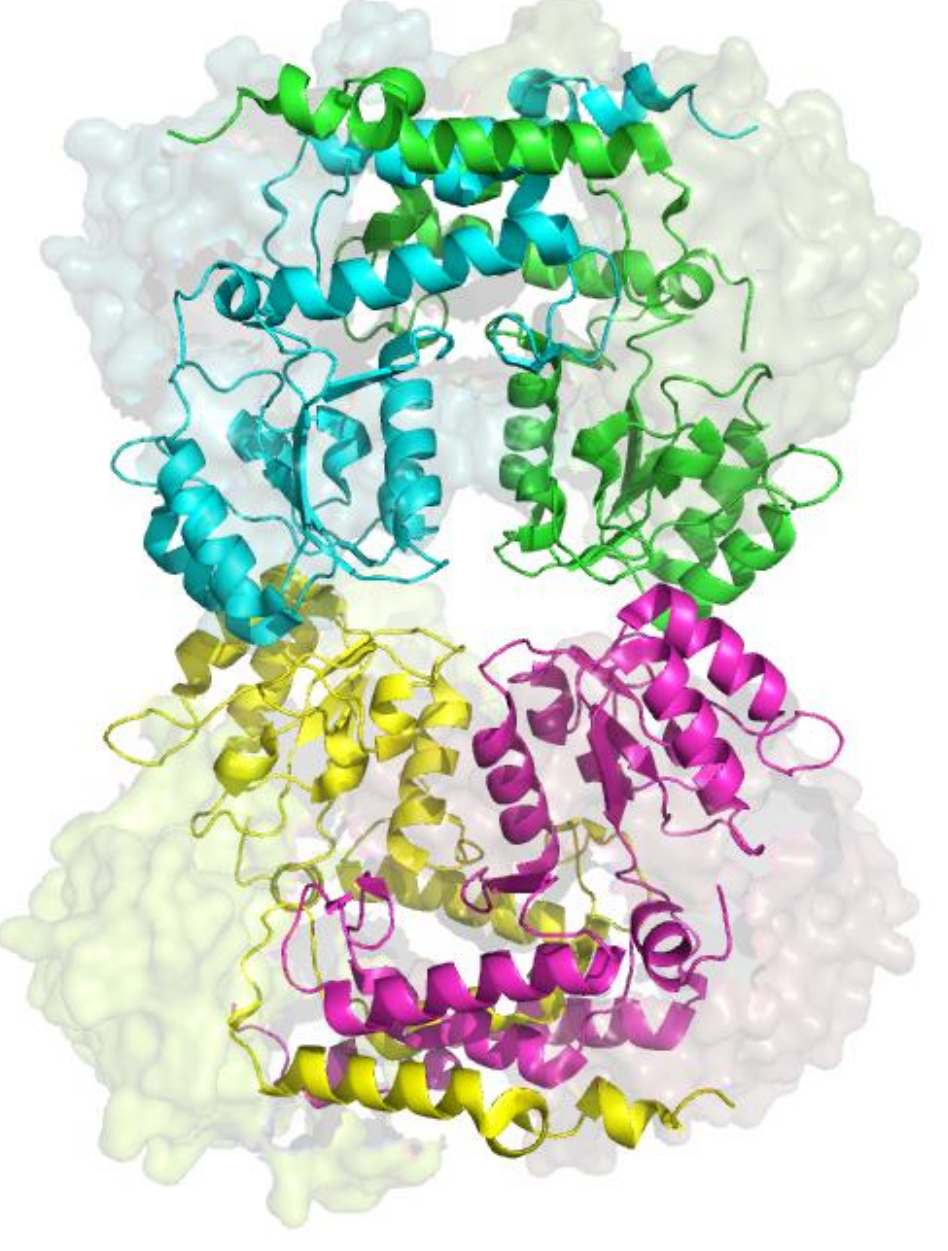

Supplementary Figure S1. The 3D structure of GmSHMT08-b tetramer (PDB ID 6UXJ), highlighting the missing regions in the F234 mutant, which has a Q226* premature stop codon. The first 226 residues of the protein are shown as ribbons. The missing region (residues 227-471) are represented by the surface. The four polypeptide chains of the tetramer are shown in contrasting colors.

	Forrest	F234	F1261
Forrest	1	0.981	0.975
F234	0.981	1	0.985
F1261	0.975	0.985	1

Supplementary Figure S2. Pairwise similarity matrix among soybean genotypes generated using the SoySNP50K Chip and analyzed in Flapjack (ver. 1.22.04.21). Similarity values indicate the degree of genetic relatedness among the genotypes based on genome-wide SNP marker profiles.

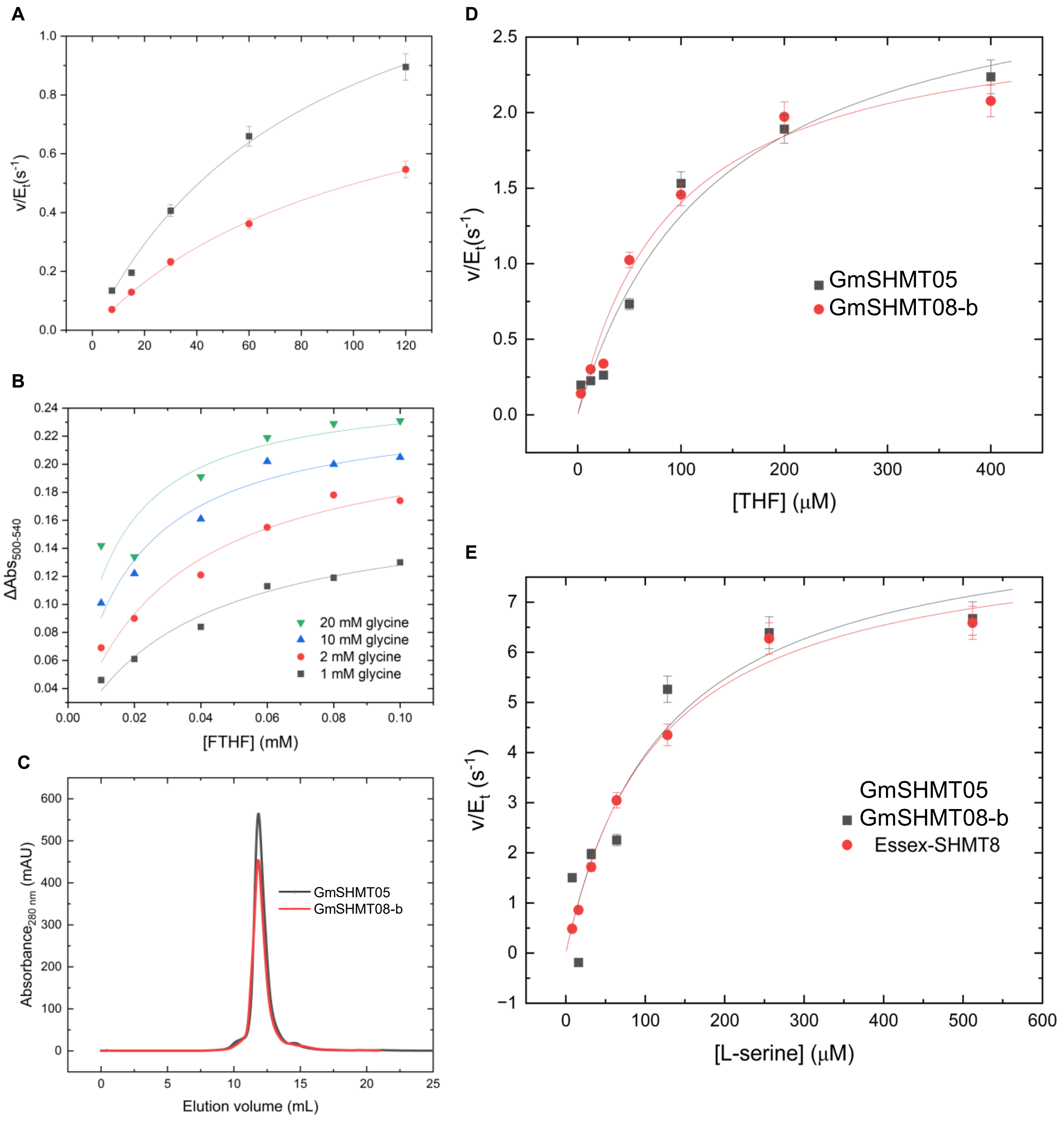

Supplementary Figure S3. Enzyme activity, ligand binding, and size exclusion chromatography data on GmSHMT05. **A)** Steady-state kinetics plots comparing GmSHMT05 (black) and GmSHMT08-b (red) for the THF-independent retro-aldol conversion of L-phenylserine, with substrate concentration varied from 0 - 120 mM. Data represent the average of three measurements; errors calculated as described in Methods. **B)** Data for the folate binding assay with GmSHMT05. Curves show Michaelis-Menten fits to the absorbance binding data as a function 5-formyl tetrahydrofolate (FTHF) concentration. Enzyme solutions at various glycine concentration were prepared and then mixed 1:1 with a solution of FTHF prior to assaying. Glycine concentrations are 1 mM (black), 2 mM (red), 10 mM (blue), and 20 mM (green). As seen with GmSHMT08-b, the binding affinity for FTHF increases as a function of glycine concentration. **C)** An overlay of the chromatograms of GmSHMT05 (black) and GmSHMT08-b (red) from size exclusion chromatography. The flow rate was 0.5 mg/ml. Both proteins elute as apparent tetramers of the same molecular weight. Fits from the MTHFD coupled assay where THF is the varied substrate **D)** at a fixed concentration of L- serine (2 mM) and where L-serine is the varied substrate **E)** at a fixed concentration of THF (1 mM). Both datasets from GmSHMT08-b and GmSHMT05 were fit to the Michaelis-Menten equation.

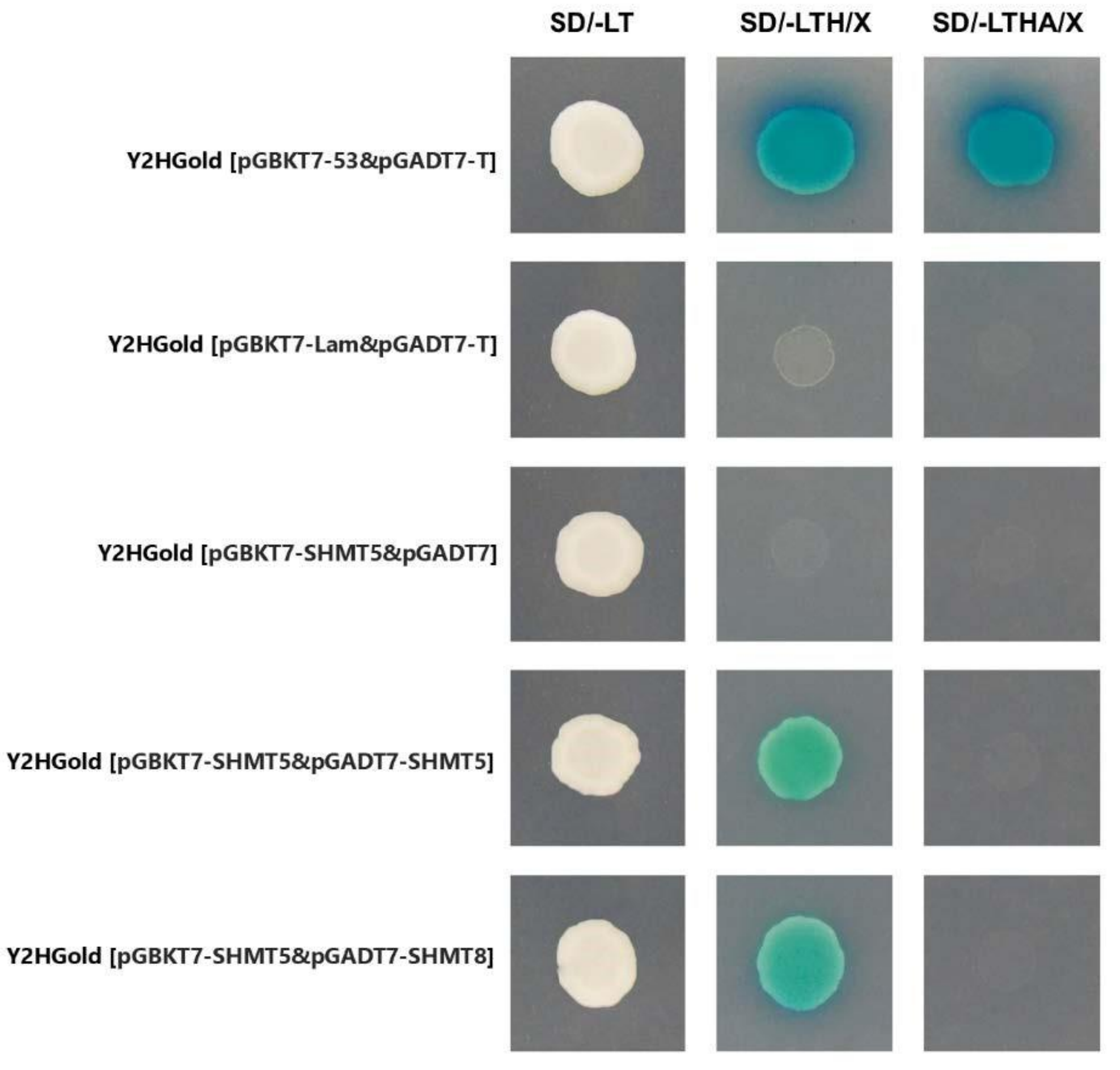

Supplementary Figure S4. The speckle pattern of interaction between SHMT5/SHMT5 and SHMT5/SHMT8 based on yeast two-hybrid system. Representative growth of Y2HGold yeast cells co-transformed with pairs of prey and bait constructs on three SD medium plates with increasing selection stringency. The treatment Y2HGold[pGBKT7-53&pGADT7-T] was a positive control; Y2HGold[pGBKT7-lam&pGADT7-T], negative control; Y2HGold[pGBKT7-SHMT5&pGADT7], the empty prey plasmids as control; Y2HGold[pGBKT7-SHMT5&pGADT7-SHMT5] and Y2HGold[pGBKT7-SHMT5&pGADT7-SHMT8] were the interaction tests. All groups had normal growth in the SD/-Leu/-Trp. Bott interaction tests had a normal growth and turned blue on SD/-Trp/-Leu/-His/X-a-gal but had no growth on SD/-Trp/-Leu/-His/-Ade/X-a-gal.

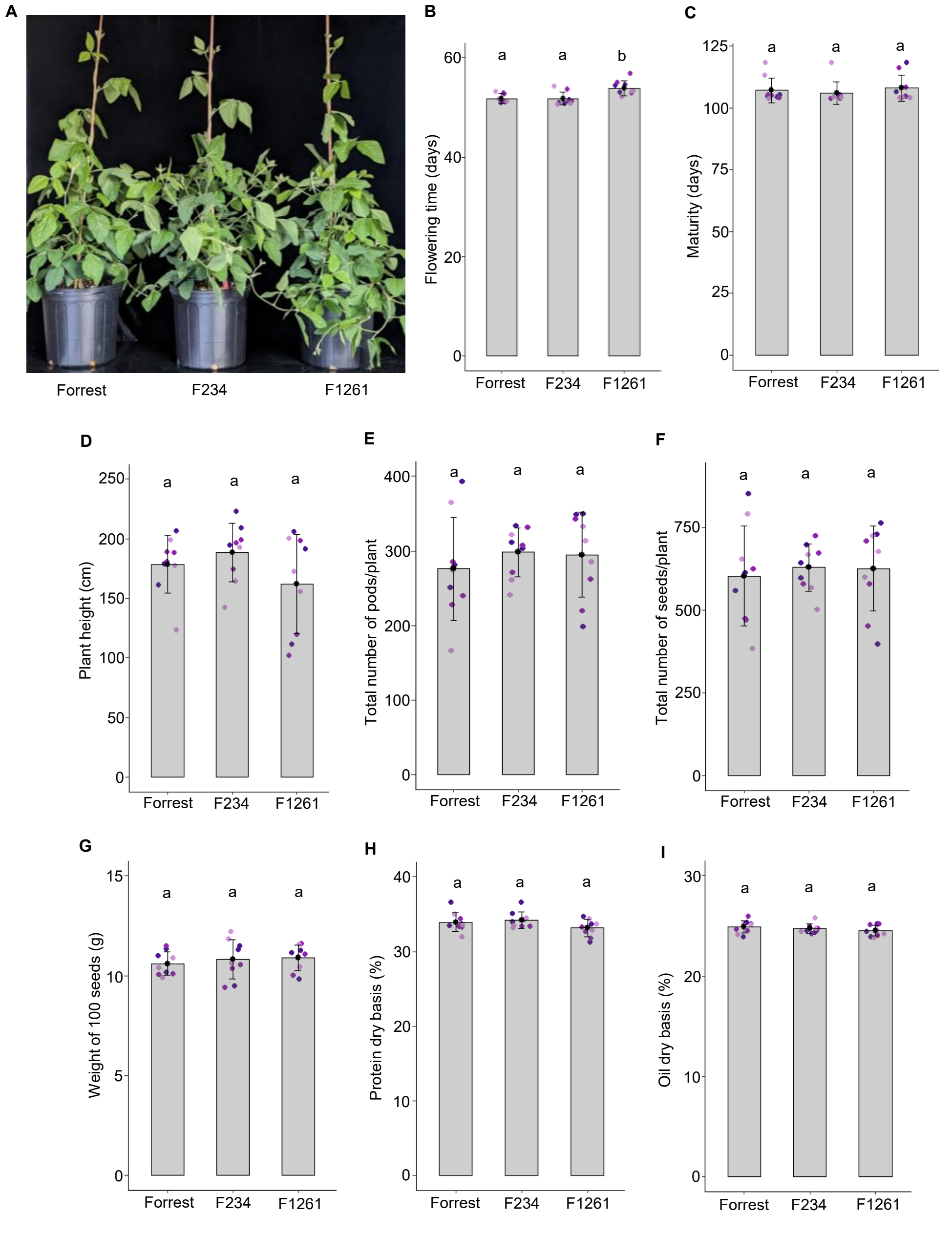

Supplementary Figure S5. Agronomically important phenotypic characters between Forrest, and two Forrest *Gmshmt08-a* mutants, F234 and F1261 in greenhouse condition. **A)** The growth of Forrest, F234 and F1261 at the flowering time. **B)** Flowering time. **C)** Maturity. D) Plant height at maturity. E) Total number of pods per plant. F) Total number of seeds per plant. G) Average weight of 100 seeds per plant. H) Average protein content dry matter basis (%). I) Average oil content dry matter basis (%). Means were compared using ANOVA followed by LSD test at $p < 0.05$. Different letters indicate significance differences. All data were as mean ± s.d. Each point represents an individual plants. Colors denote blocks.

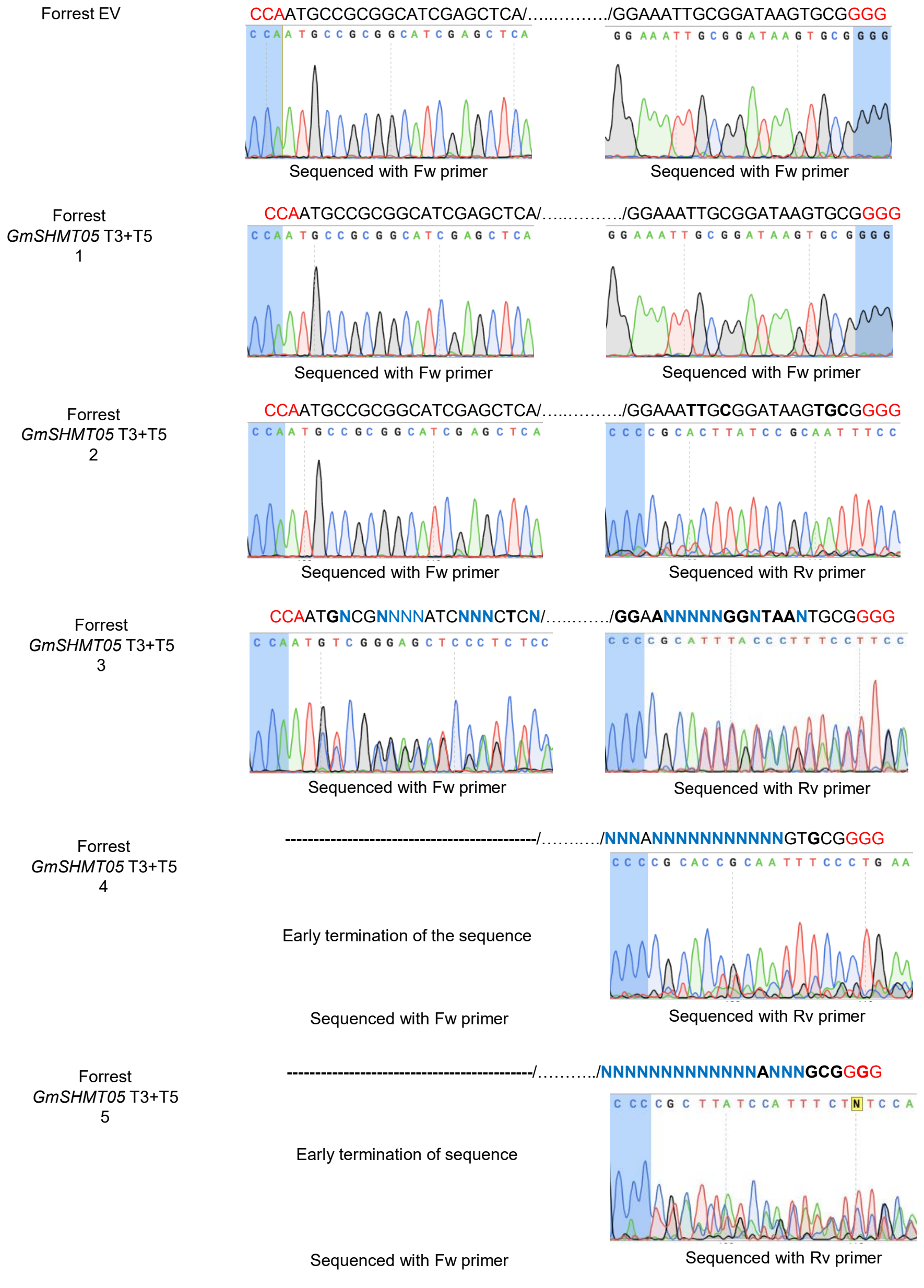

Supplementary Figure S6. Amplicon sequencing to confirm editing of *GmSHMT05* in Forrest targeted by CRISPR/Cas9-*GmSHMT05*-T3+T5 in replicate 1. Genomic DNA was extracted from transgenic hairy roots and the targeted fragments were amplified by PCR. Direct sequencing of PCR products confirmed successful gene edits, as indicated by the presence of deletions/insertions and re-annealing at regions targeted by sgRNA. The **bolded** bases indicate multiple peaks at that position, but the dominant base matches the WT based on the sequence. The blue colored "**N**" denotes chimeric peaks where bases differ from WT, representing a mixture of WT bases and newly introduced bases across multiple amplified alleles. PAM sequence is in red color.

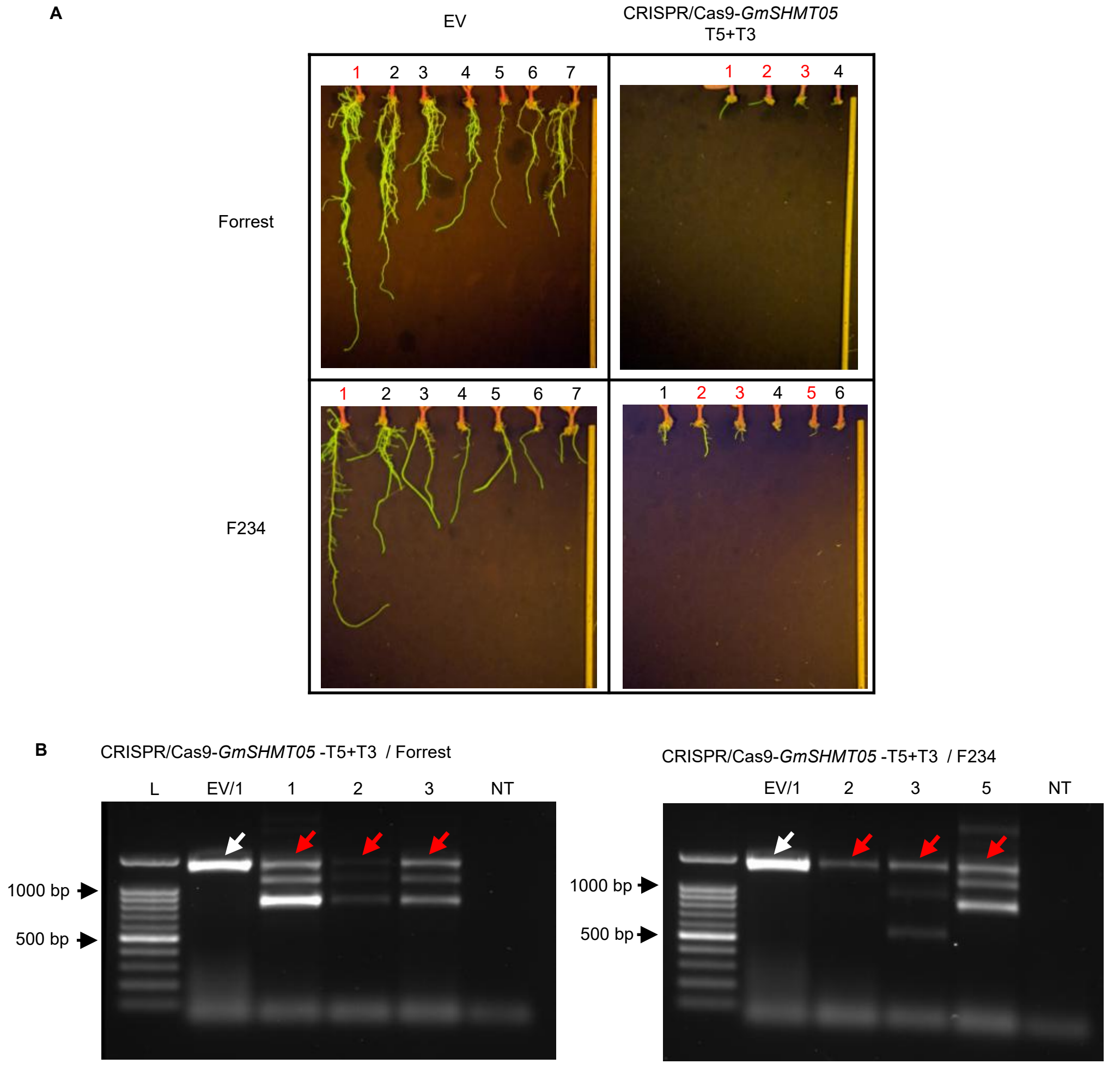

C

	T3 … 496 bp … T5	WT/ Edited (chimeric/indel)
Forrest/F234-EV	CCAATGCCGCGGCATCGAGCTCA/............ /GGAAATTGCGGATAAGTGCGGGG	WT
Forrest/1	CCAATNCCGCGGCATCGAGCTCA/............/GGAAATTGCGGAT---------GCGGGG	Chimeric
Forrest/2	CCAATGNCGCGGCATCGAGCTCA/............/NNNNNNNNNNNNNNNNTGCGGGG	Chimeric
Forrest/3	CCAATGCCGCGGCATCGAGCTCA/............/GGAAATTGNGGANAAGTGCGGGG	Chimeric
F234/2	CCAATGCCGCGGCATCGAGCTCA/ ..../GGAAATTGCGGATAAGTGCGGGG	Chimeric (WT dominant)
F234/3	CNNNNNNNNNNNNNNNNNNNNNNN/............/NNNNNNNNNNNNNNNNTGCGGGG	Chimeric
F234/5	CCAATGNCNNNNNNTNNNNNNNN/............/NNNNNNNNNNNNNNNGTGCGGGG	Chimeric

Supplementary Figure S7. Targeted genome editing of *GmSHMT05* in Forrest and F234 by CRISPR/Cas9-*GmSHMT05*-T3+T5 in replicate 2. **A**. Plants with GFP (+) roots inoculated with K599 carrying EV and CRISPR/Cas9-*GmSHMT05*-T3+T5. Genomic DNA was extracted from transgenic hairy roots from plants indicated by red color. **B**. Gel picture of *GmSHMT05* amplification flanking T3 and T5 region using F1 and R1. Red arrows indicate the bands with edits and white arrows indicate the band with WT sequences. **C**. Sequences of extracted bands indicated by arrows in B. The **bolded** bases indicate multiple peaks at that position, but the dominant base matches the WT based on the sequence. The blue colored "**N**" denotes chimeric peaks where bases differ from WT, representing a mixture of WT bases and newly introduced bases across multiple amplified alleles. PAM sequence is in red color. L = 100 bp ladder. WT = Wild Type. NT = No Template

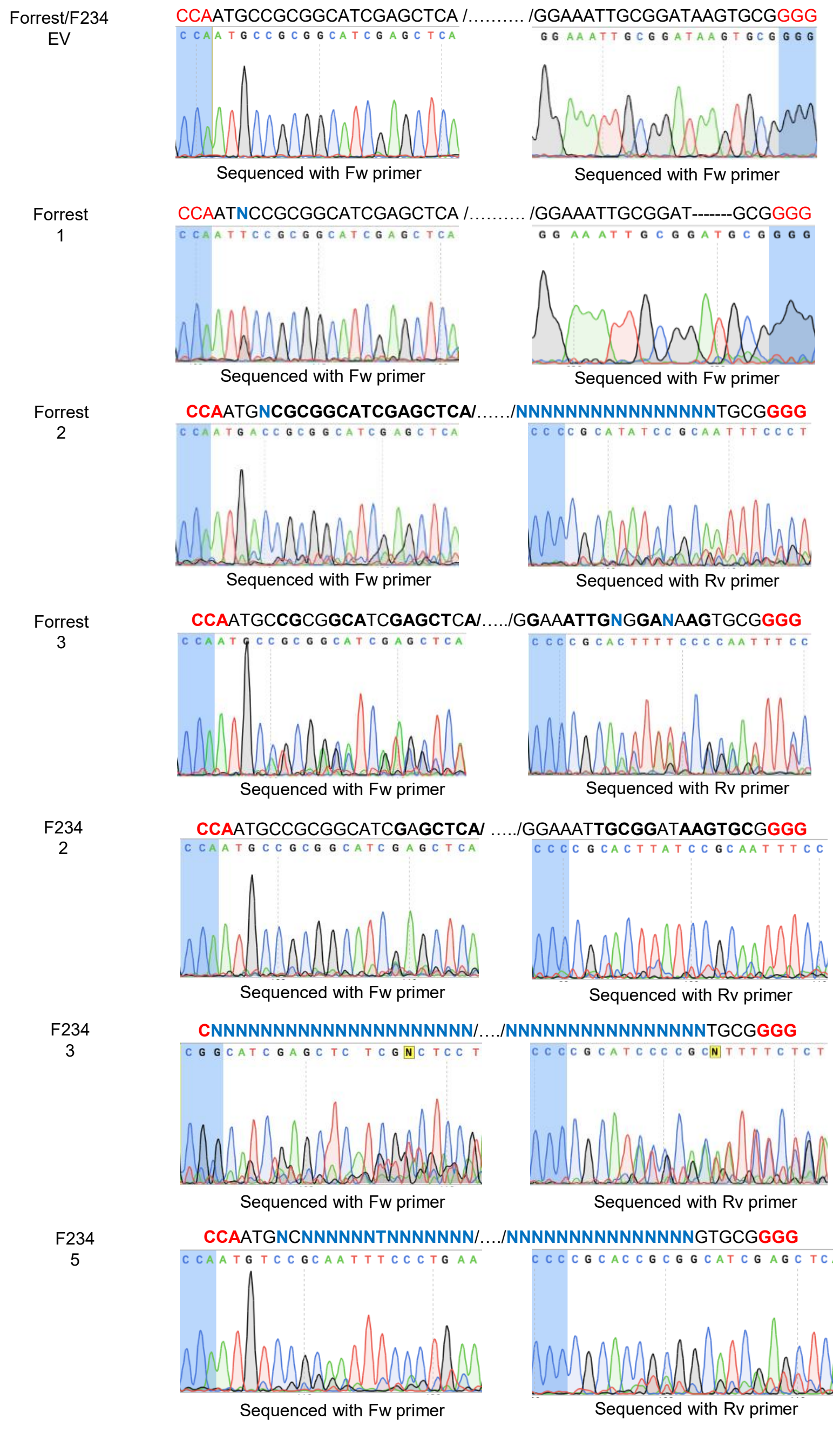

Supplementary Figure S8. Amplicon sequencing to confirm editing of *GmSHMT05* in Forrest and F234 by CRISPR/Cas9-*GmSHMT05*-T3+T5 in replicate 2. Genomic DNA was extracted from transgenic hairy roots and the targeted fragments were amplified by PCR. Direct sequencing of PCR products confirmed successful gene edits, as indicated by the presence of deletions/insertions and re-annealing at regions targeted by sgRNA. The **bolded** bases indicate multiple peaks at that position, but the dominant base matches the WT based on the sequence. The blue colored "**N**" denotes chimeric peaks where bases differ from WT, representing a mixture of WT bases and newly introduced bases across multiple amplified alleles. PAM sequence is in red color.

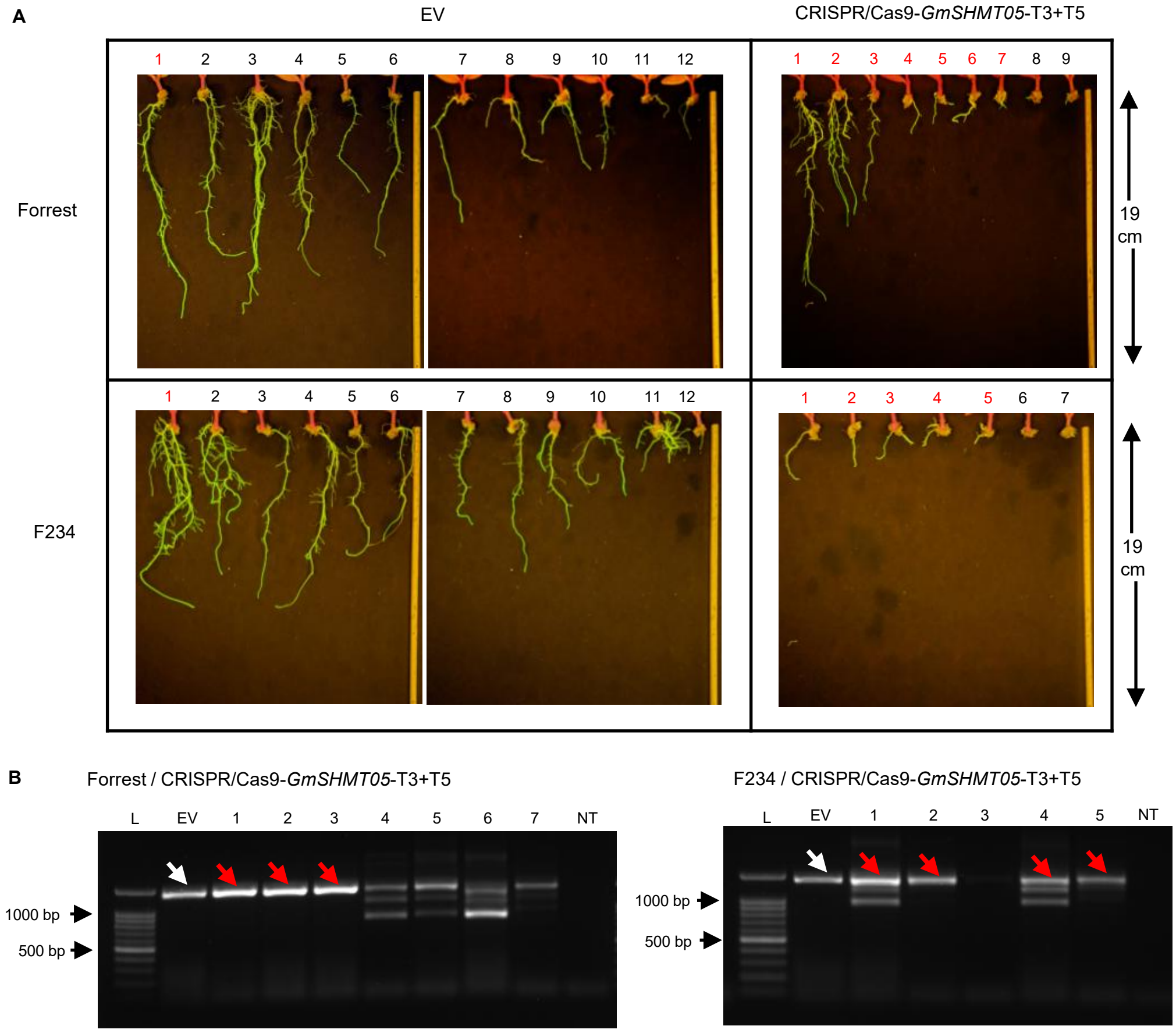

C

	T3 — 496 bp — T5	WT/ Edited (chimeric/indel)
Forrest/F234-EV	CCAATGCCGCGGCATCGAGCTCA /.............. /GGAAATTGCGGATAAGTGCGGGG	WT
Forrest/1	CCAATG**CCNCNGNATNNANCTNA/....**.........../GGAAATTGCGGATAAGTGCGGGG	Chimeric
Forrest/2	CCAATGCCGCGGCATC**GAGCTCA/....** .... /GGAAATTGCGGATAAGTGCGGGG	Chimeric (WT dominant)
Forrest/3	CCAATGCCGCGGCATC**G**AGCTCA /............... /GGAAATTGCGGATAAGTGCGGGG	Chimeric (WT dominant)
F234/1	CCAATGCCGCGGCATC**G**A**GCTCA** /............/GG**AAATTGCGGATAAGT**GCGGGG	Chimeric (WT dominant)
F234/2	**NNANTNCNNCNNCNNCNTCGNNCTCA** /.........../**NNNNNNNNNNNNNNNN**TGCGGGG	Chimeric
F234/4	CCAATGC**CNCGNNNNNNNNNNNN** /............/----------------did not sequence-----------	Chimeric
F234/5	CCAATGCC**NNNNNNNNNNNNNNN** /............ /---------------did not sequence------------	Chimeric

Supplementary Figure S9. Targeted genome editing of *GmSHMT05* in Forrest and F234 by CRISPR/Cas9-*GmSHMT05*-T3+T5 in replicate 3. **A**. Plants with GFP (+) roots inoculated with K599 carrying EV and CRISPR/Cas9-*GmSHMT05*-T3+T5. Genomic DNA was extracted from transgenic hairy roots from plants indicated by red color. **B**. Gel picture of *GmSHMT05* amplification flanking T3 and T5 region using F1 and R1. Red arrows indicate the bands with edits and white arrows indicate the band with WT sequences. **C**. Sequences of extracted bands indicated by arrows in B. The **bolded** bases indicate multiple peaks at that position, but the dominant base matches the WT based on the sequence. The blue colored "**N**" denotes chimeric peaks where bases differ from WT, representing a mixture of WT bases and newly introduced bases across multiple amplified alleles. PAM sequence is in red color. L = 100 bp ladder. WT = Wild Type. NT = No Template

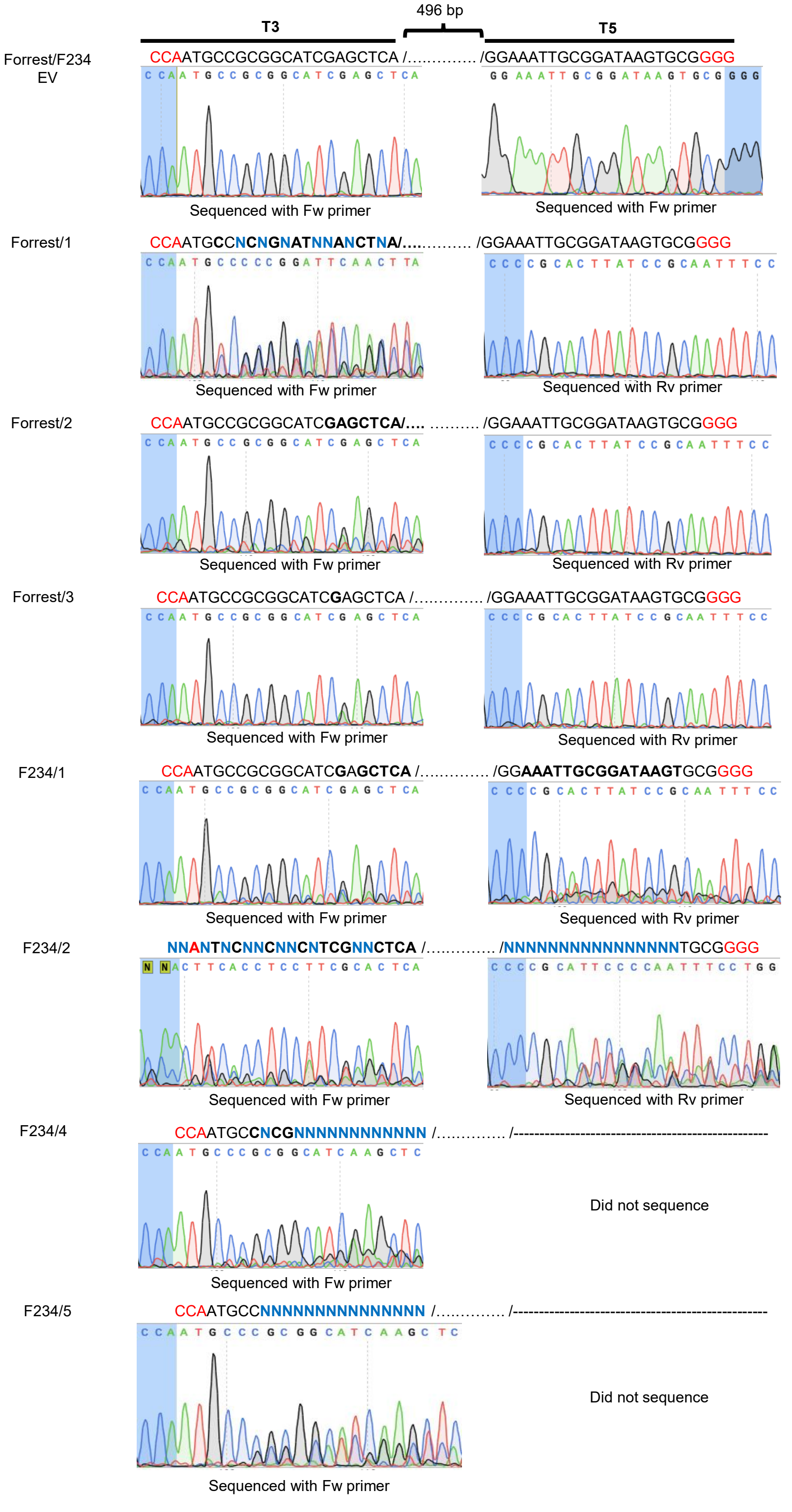

Supplementary Figure S10. Targeted genome editing of *GmSHMT05* in Forrest and F234 by CRISPR/Cas9-*GmSHMT05*-T3+T5 dual gRNA system in replicate 3. Genomic DNA was extracted from transgenic hairy roots and the targeted fragments were amplified by PCR. Direct sequencing of PCR products confirmed successful gene edits, as indicated by the presence of deletions/insertions and re-annealing at regions targeted by sgRNA. The **bolded** bases indicate multiple peaks at that position, but the dominant base matches the WT based on the sequence. The blue colored "**N**" denotes chimeric peaks where bases differ from WT, representing a mixture of WT bases and newly introduced bases across multiple amplified alleles. PAM sequence is in red color.

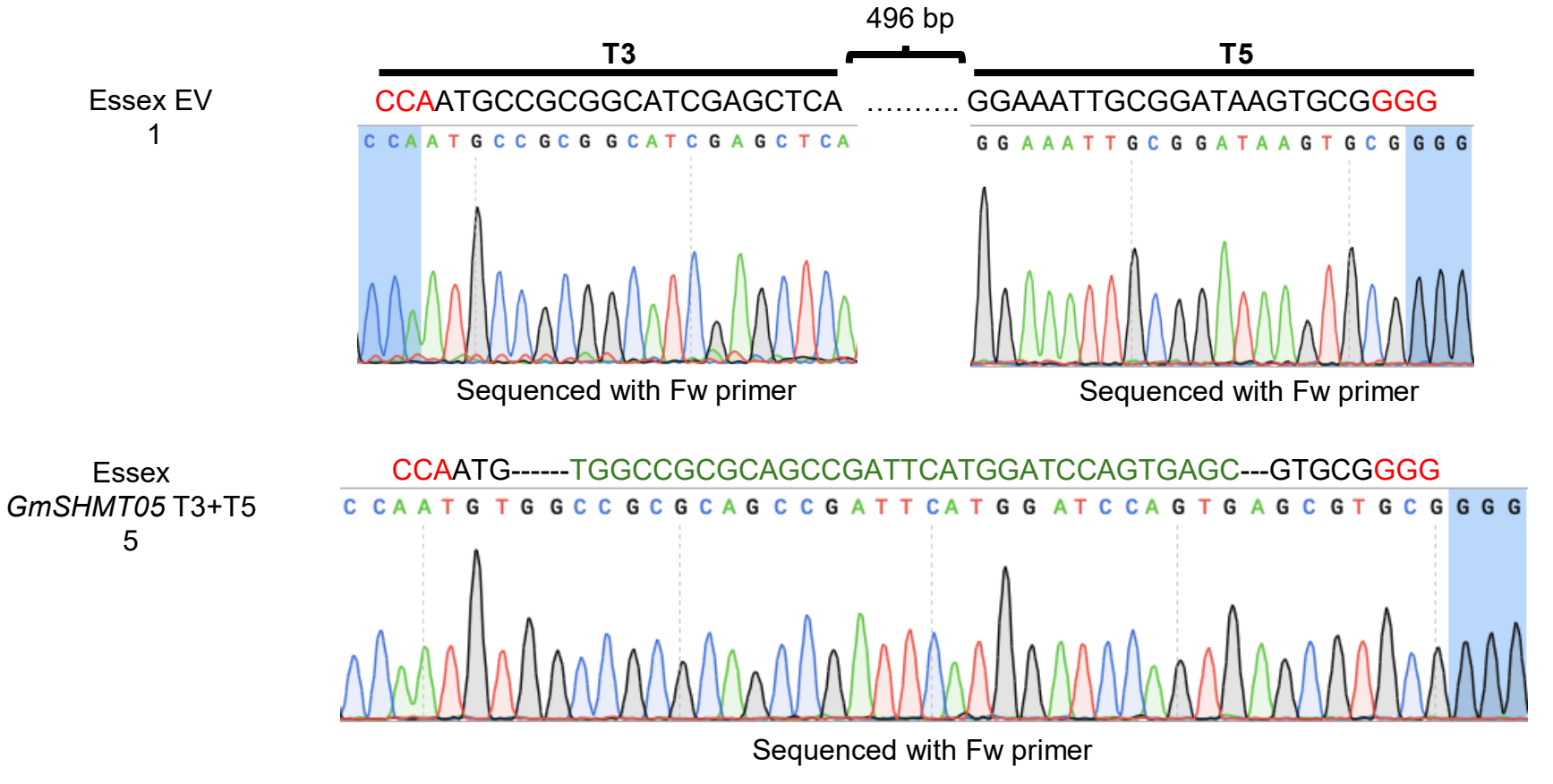

Supplementary Figure S11. Amplicon sequencing to confirm editing of *GmSHMT05* in Essex by CRISPR/Cas9-*GmSHMT05*-T3+T5 in replicate 1. Genomic DNA was extracted from transgenic hairy roots and the targeted fragments were amplified by PCR. Direct sequencing of PCR products confirmed successful gene edits, as indicated by the presence of deletions/insertions and re-annealing at regions targeted by sgRNA. The **bolded** bases indicate multiple peaks at that position, but the dominant base matches the WT based on the sequence. The blue colored "**N**" denotes chimeric peaks where bases differ from WT, representing a mixture of WT bases and newly introduced bases across multiple amplified alleles. PAM sequence is in red color.

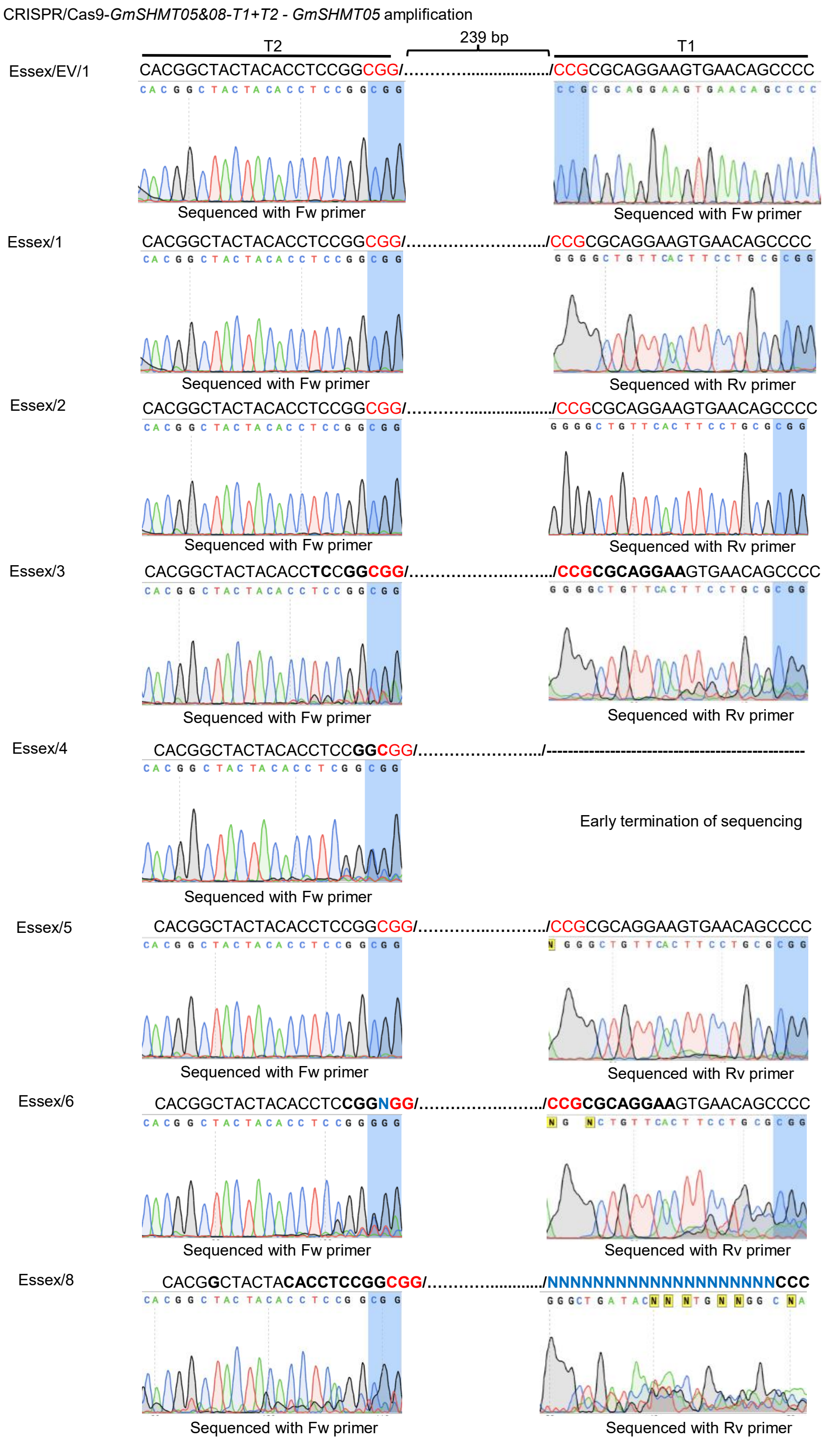

Supplementary Figure S12. Amplicon sequencing to confirm editing of *GmSHMT05* in Essex targeted by CRISPR/Cas9-*GmSHMT05&08*-T1+T2 in replicate 1. Genomic DNA was extracted from transgenic hairy roots and the targeted fragments were amplified by PCR. Direct sequencing of PCR products confirmed successful gene edits in *GmSHMT05*, as indicated by the presence of deletions/insertions and re-annealing at regions targeted by sgRNA. The **bolded** bases indicate multiple peaks at that position, but the dominant base matches the WT based on the sequence. The blue colored "**N**" denotes chimeric peaks where bases differ from WT, representing a mixture of WT bases and newly introduced bases across multiple amplified alleles. PAM sequence is in red color.

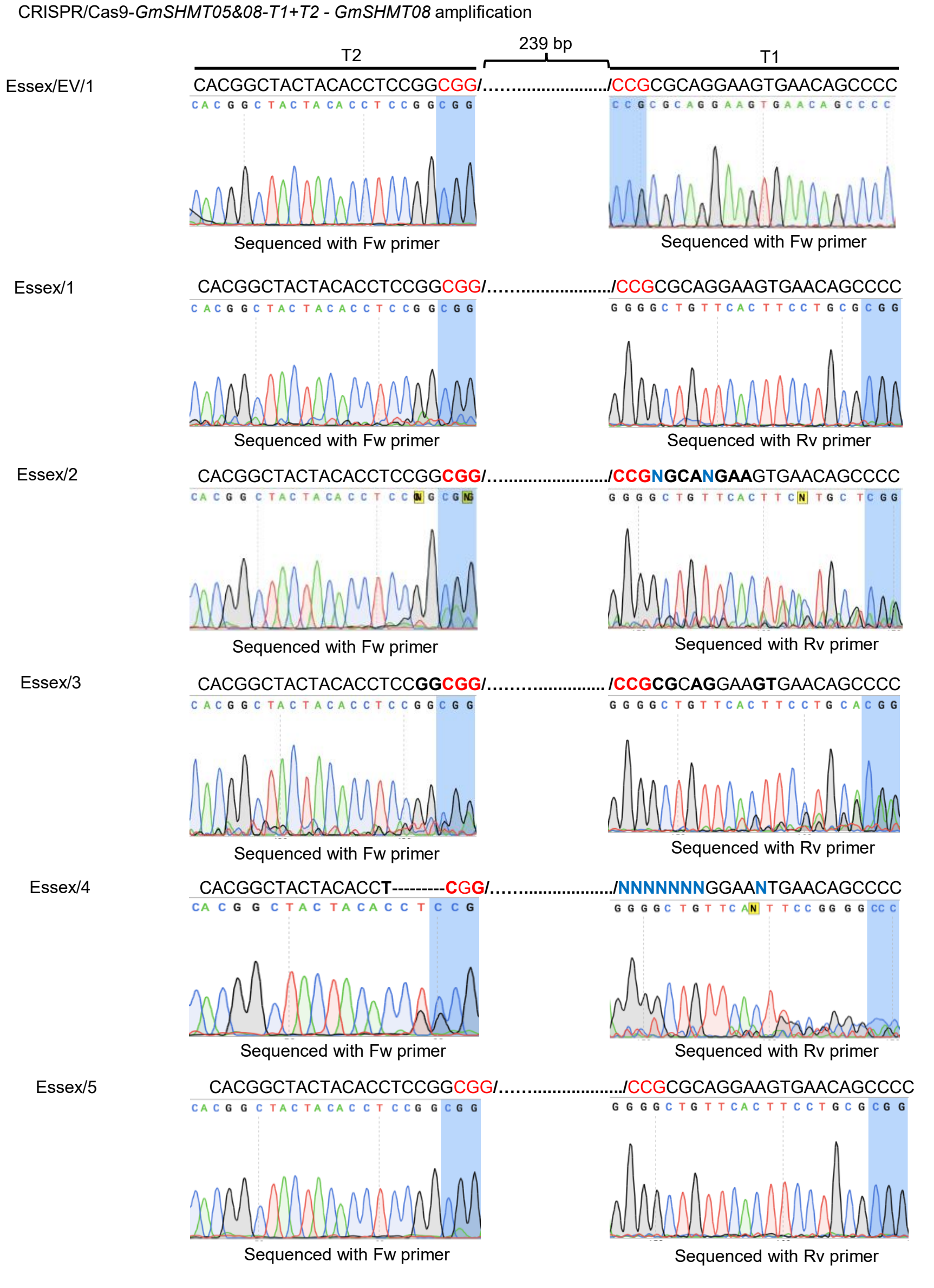

Supplementary Figure S13. Amplicon sequencing to confirm the editing of *GmSHMT08* in Essex targeted by CRISPR/Cas9-*GmSHMT05&08*-T1+T2 in replicate 1. Genomic DNA was extracted from transgenic hairy roots and the targeted fragments were amplified by PCR. Direct sequencing of PCR products confirmed successful gene edits in *GmSHMT08*, as indicated by the presence of deletions/insertions and re-annealing at regions targeted by sgRNA. The **bolded** bases indicate multiple peaks at that position, but the dominant base matches the WT based on the sequence. The blue colored "**N**" denotes chimeric peaks where bases differ from WT, representing a mixture of WT bases and newly introduced bases across multiple amplified alleles. PAM sequence is in red color.

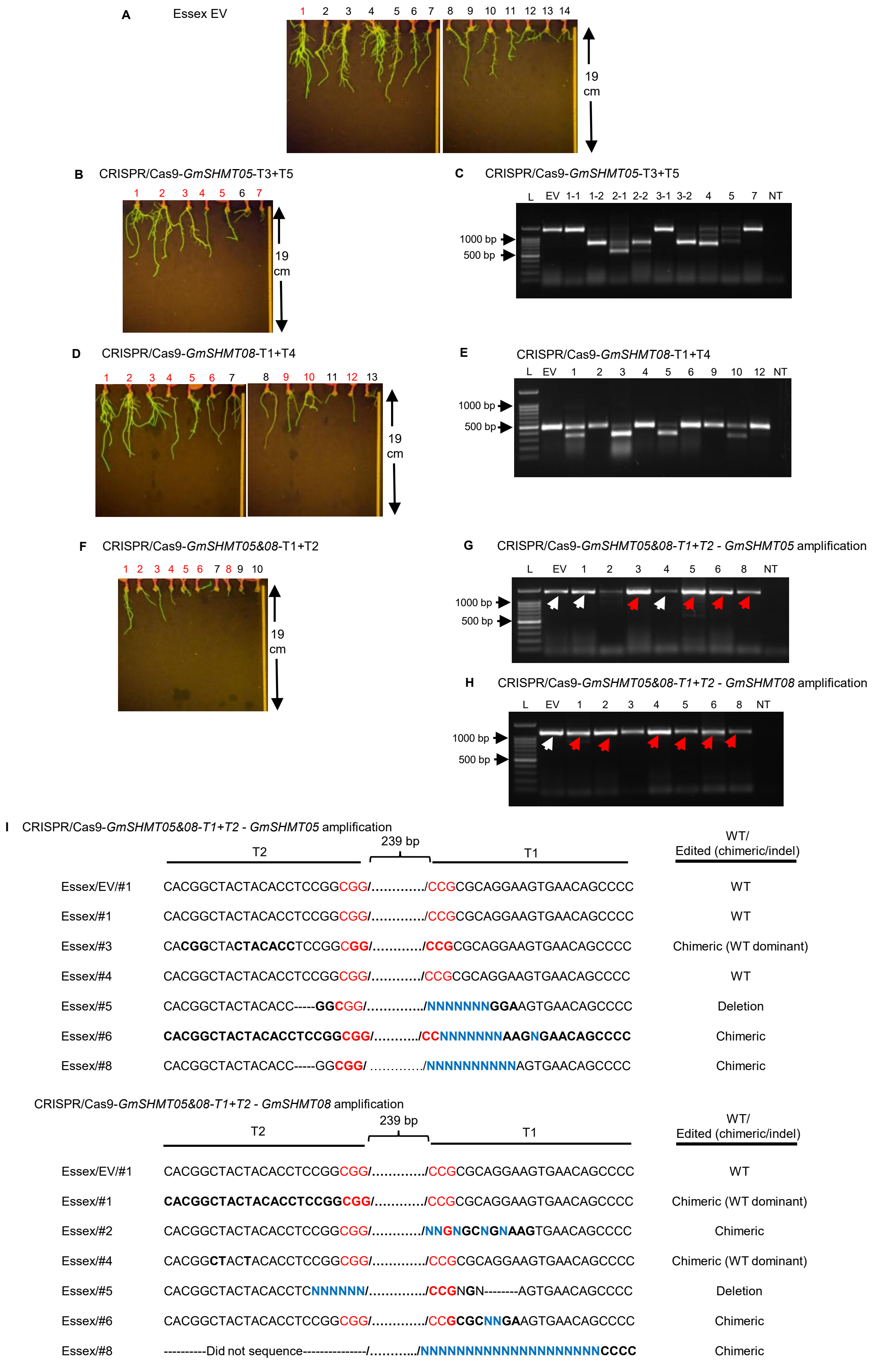

A
Essex EV
1 2 3 4 5 6 7 8 9 10 11 12 13 14
19 cm
B
CRISPR/Cas9-GmSHMT05-T3+T5
1 2 3 4 5 6 7
19 cm
C
CRISPR/Cas9-GmSHMT05-T3+T5
L EV 1-1 1-2 2-1 2-2 3-1 3-2 4 5 7 NT
1000 bp
500 bp
D
CRISPR/Cas9-GmSHMT08-T1+T4
1 2 3 4 5 6 7 8 9 10 11 12 13
19 cm
E
CRISPR/Cas9-GmSHMT08-T1+T4
L EV 1 2 3 4 5 6 9 10 12 NT
1000 bp
500 bp
F
CRISPR/Cas9-GmSHMT05&08-T1+T2
1 2 3 4 5 6 7 8 9 10
19 cm
G
CRISPR/Cas9-GmSHMT05&08-T1+T2 - GmSHMT05 amplification
L EV 1 2 3 4 5 6 8 NT
1000 bp
500 bp
H
CRISPR/Cas9-GmSHMT05&08-T1+T2 - GmSHMT08 amplification
L EV 1 2 3 4 5 6 8 NT
1000 bp
500 bp
I
CRISPR/Cas9-GmSHMT05&08-T1+T2 - GmSHMT05 amplification
T2
239 bp
T1
WT/ Edited (chimeric/indel)
Essex/EV/#1 CACGGCTACTACACCTCCGGCGG/............./CCGCGCAGGAAGTGAACAGCCCC WT
Essex/#1 CACGGCTACTACACCTCCGGCGG/............./CCGCGCAGGAAGTGAACAGCCCC WT
Essex/#3 CACGGCTACTACACCTCCGGCGG/............/CCGCGCAGGAAGTGAACAGCCCC Chimeric (WT dominant)
Essex/#4 CACGGCTACTACACCTCCGGCGG/............/CCGCGCAGGAAGTGAACAGCCCC WT
Essex/#5 CACGGCTACTACACC-----GGCGG/............../NNNNNNNGGAAGTGAACAGCCCC Deletion
Essex/#6 CACGGCTACTACACCTCCGGCGG/.........../CCNNNNNNNAAGNGAACAGCCCC Chimeric
Essex/#8 CACGGCTACTACACC-----GGCGG/ ..../NNNNNNNNNAGTGAACAGCCCC Chimeric
CRISPR/Cas9-GmSHMT05&08-T1+T2 - GmSHMT08 amplification
T2
239 bp
T1
WT/ Edited (chimeric/indel)
Essex/EV/#1 CACGGCTACTACACCTCCGGCGG/............./CCGCGCAGGAAGTGAACAGCCCC WT
Essex/#1 CACGGCTACTACACCTCCGGCGG/............/CCGCGCAGGAAGTGAACAGCCCC Chimeric (WT dominant)
Essex/#2 CACGGCTACTACACCTCCGGCGG/............/NNGNGCNGNAAGTGAACAGCCCC Chimeric
Essex/#4 CACGGCTACTACACCTCCGGCGG/............./CCGCGCAGGAAGTGAACAGCCCC Chimeric (WT dominant)
Essex/#5 CACGGCTACTACACCTCNNNNNN/............../CCGNGN--------AGTGAACAGCCCC Deletion
Essex/#6 CACGGCTACTACACCTCCGGCGG/............./CCGCGCNNGAAGTGAACAGCCCC Chimeric
Essex/#8 ----------Did not sequence---------------/.........../NNNNNNNNNNNNNNNNNNNNCCCC Chimeric

Supplementary Figure S14. Targeted genome editing of *GmSHMT05* and *GmSHMT08* individually and simultaneously in Essex by CRISPR/Cas9-*GmSHMT05*-T3+T5, CRISPR/Cas9-*GmSHMT08*-T1+T4 and CRISPR/Cas9-*GmSHMT05&08*-T1+T2 dual gRNA system in replicate 2. **A**. Plants with GFP (+) roots inoculated with K599 carrying EV **B**. GFP (+) roots of Essex plants inoculated with K599 carrying CRISPR/Cas9-*GmSHMT05*-T3+T5. **C**. GFP (+) roots of Essex plants inoculated with K599 carrying CRISPR/Cas9-*GmSHMT08*-T1+T4. **D**. GFP (+) roots of Essex plants inoculated with K599 carrying CRISPR/Cas9-*GmSHMT05&08*-T1+T2. Roots selected for DNA extraction are in red. **E**. Gel picture of amplified fragments from genomic DNA extracted from roots transformed with CRISPR/Cas9-*GmSHMT05*-T3+T5. Fragments flanking T3 and T5 gRNA cleavage sites were amplified using F1 and R1 primers. **F**. Gel picture of amplified fragments from genomic DNA extracted from roots transformed with CRISPR/Cas9-*GmSHMT08*-T1+T4. Fragments flanking T1 and T4 gRNA cleavage sites were amplified using F2 and R2 primers. **G**. Gel picture of amplified fragments of *GmSHMT05* from genomic DNA extracted from roots transformed with CRISPR/Cas9-*GmSHMT05&08*-T1+T2. Fragments flanking T1 and T2 gRNA cleavage sites were amplified using F1 and R1 primers. **H**. Gel picture of amplified fragments of *GmSHMT08* from genomic DNA extracted from roots transformed with CRISPR/Cas9-*GmSHMT05&08*-T1+T2. Fragments flanking T1 and T2 gRNA cleavage sites were amplified using F3 and R3 primers. Arrows indicate the gel bands extracted for direct sequencing. White arrows indicate bands with WT fragments. Red arrows indicate bands with edited sequences. **I**. Sequences of selected bands in G and H. The editing of genes were confirmed. WT indicates non edited sequences. Chimeric indicates the mixture of WT and edited sequences. If WT is dominant in chmeric sequences, it is denoted as chimeric (WT dominant). The **bolded** bases indicate multiple peaks at that position, but the dominant base matches the WT based on the sequence. The blue colored "**N**" denotes chimeric peaks where bases differ from WT, representing a mixture of WT bases and newly introduced bases across multiple amplified alleles. PAM sequence is in red color. L = 100 bp ladder. WT = Wild Type. NT = No Template

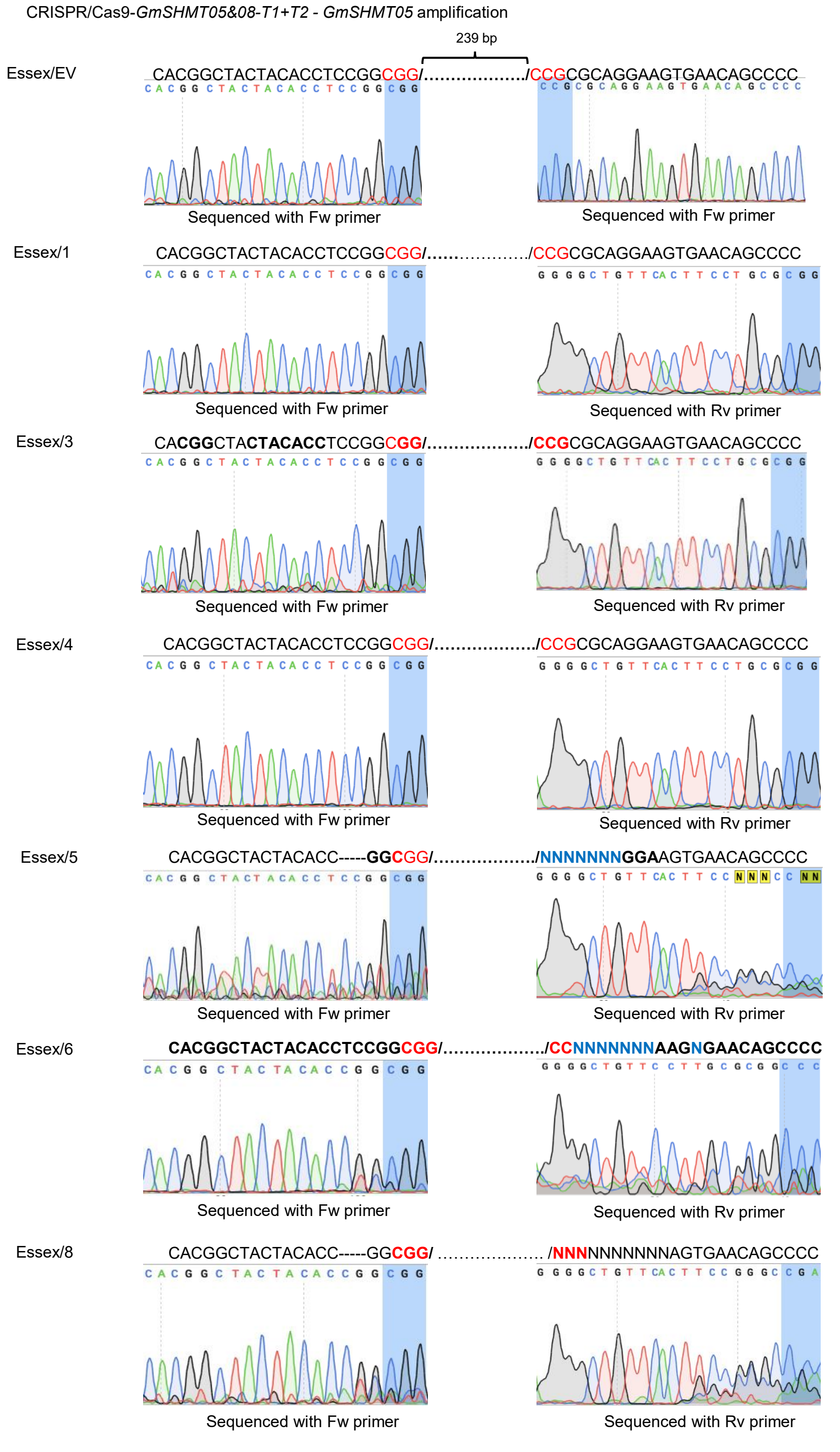

Supplementary Figure S15. Amplicon sequencing to confirm editing of *GmSHMT05* in Essex targeted by CRISPR/Cas9-*GmSHMT05&08*-T1+T2 in replicate 2. Genomic DNA was extracted from transgenic hairy roots and the targeted fragments were amplified by PCR. Direct sequencing of PCR products confirmed successful gene edits in *GmSHMT05*, as indicated by the presence of deletions/insertions and re-annealing at regions targeted by sgRNA. The **bolded** bases indicate multiple peaks at that position, but the dominant base matches the WT based on the sequence. The blue colored "**N**" denotes chimeric peaks where bases differ from WT, representing a mixture of WT bases and newly introduced bases across multiple amplified alleles. PAM sequence is in red color.

CRISPR/Cas9-*GmSHMT05&08-T1+T2* - *GmSHMT08* amplification

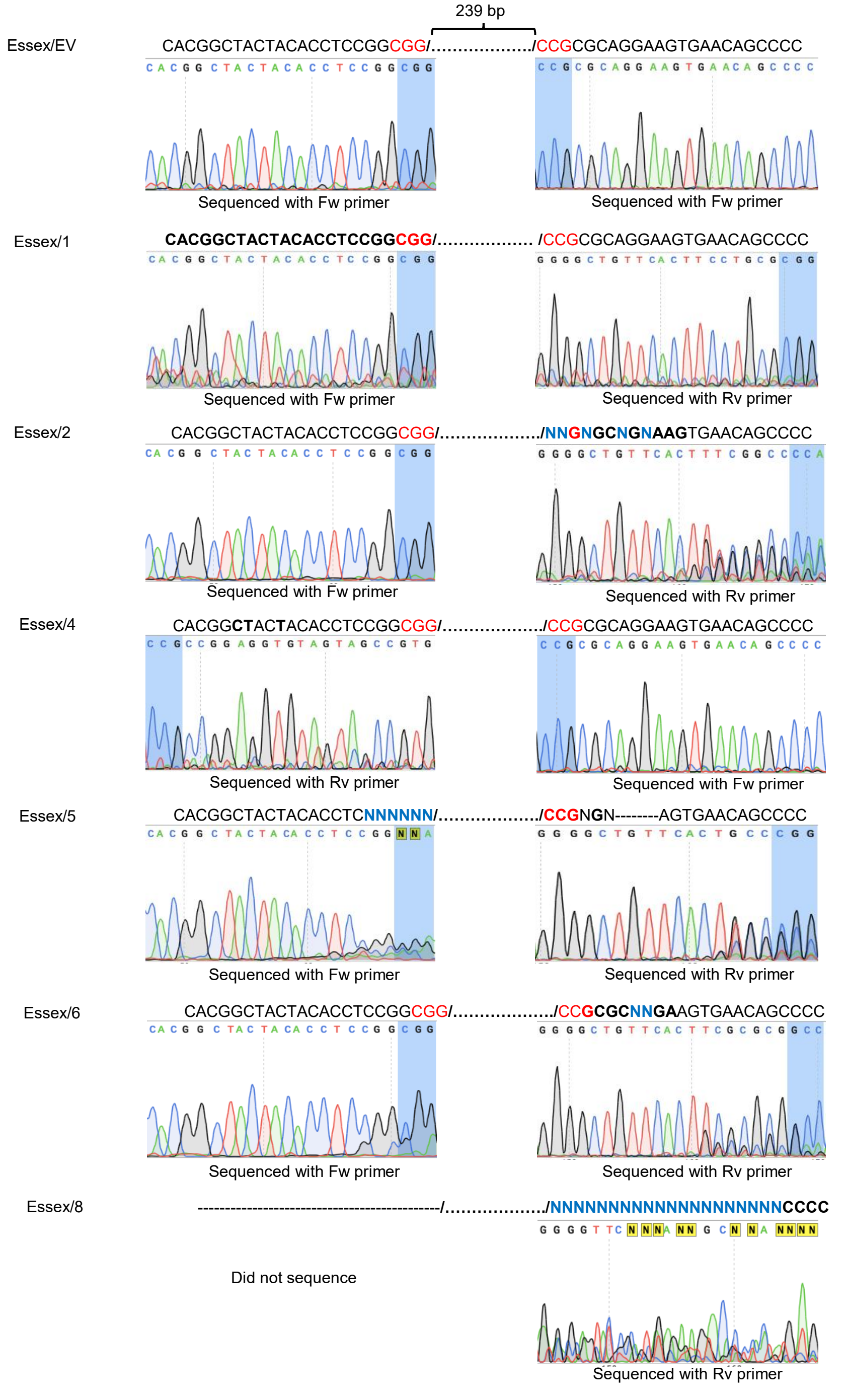

Supplementary Figure S16. Amplicon sequencing to confirm the editing of *GmSHMT08* in Essex by CRISPR/Cas9-*GmSHMT05&08*-T1+T2 in replicate 2. Genomic DNA was extracted from transgenic hairy roots and the targeted fragments were amplified by PCR. Direct sequencing of PCR products confirmed successful gene edits in *GmSHMT08*, as indicated by the presence of deletions/insertions and re-annealing at regions targeted by sgRNA. The **bolded** bases indicate multiple peaks at that position, but the dominant base matches the WT based on the sequence. The blue colored "**N**" denotes chimeric peaks where bases differ from WT, representing a mixture of WT bases and newly introduced bases across multiple amplified alleles. PAM sequence is in red color.

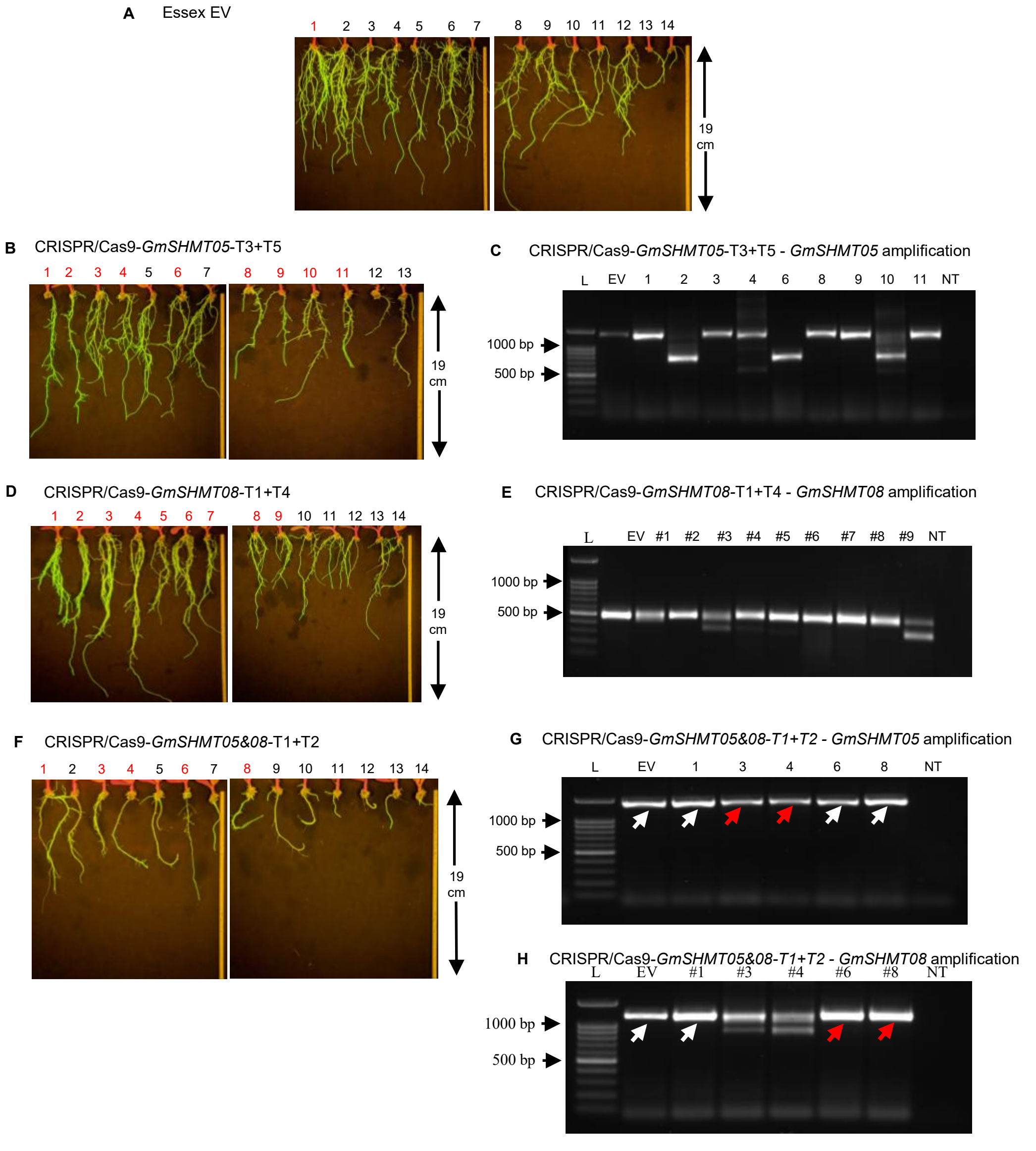

I CRISPR/Cas9-*GmSHMT05&08-T1+T2* - *GmSHMT05* amplification

	T2 — 239 bp — T1	WT/ Edited (chimeric/indel)
Essex/EV/1	CACGGCTACTACACCTCCGGCGG/............./CCGCGCAGGAAGTGAACAGCCCC	WT
Essex/1	CACGGCTACTACACCTCCGGCGG/............./CCGCGCAGGAAGTGAACAGCCCC	WT
Essex/3	CACGGCTACTACA**CCTCCGGCGG**/............./**CCGCGCA**GG**AA**GTGAACAGCCCC	Chimeric (WT dominant)
Essex/4	CACGGCTACTACACC**T**CCGG**CGG**/............./**CCGCGCAGGAAG**TGAACAGCCCC	Chimeric (WT dominant)
Essex/6	CACGGCTACTACACCTCCGGCGG/............./CCGCGCAGGAAGTGAACAGCCCC	WT
Essex/8	CACGGCTACTACACCTCCGGCGG/............./CCGCGCAGGAAGTGAACAGCCCC	WT

CRISPR/Cas9-*GmSHMT05&08-T1+T2* - *GmSHMT08* amplification

	T2 — 239 bp — T1	WT/ Edited (chimeric/indel)
Essex/EV/1	CACGGCTACTACACCTCCGGCGG/............./CCGCGCAGGAAGTGAACAGCCCC	WT
Essex/1	CACGGCTACTACACCTCCGGCGG/............./CCGCGCAGGAAGTGAACAGCCCC	WT
Essex/6	**CA**C**NNN**T**A**N**T**ACA**CC**T**C**CCG**N**CGG/............./CCGCGCA**N**G**AA**N**TG**AAC**NN**C**C**C**C**	Chimeric
Essex/8	CACGGCTACTACACCTC**CG**GCGG/............./**NNNN**GCNGNNNGTGAACAGCCCC	Chimeric

Supplementary Figure S17. Targeted genome editing of *GmSHMT05* and *GmSHMT08* individually and simultaneously in Essex by CRISPR/Cas9-*GmSHMT05*-T3+T5, CRISPR/Cas9-*GmSHMT08*-T1+T4 and CRISPR/Cas9-*GmSHMT05&08*-T1+T2 dual gRNA system in replicate 3. **A**. Plants with GFP (+) roots inoculated with K599 carrying EV **B**. GFP (+) roots of Essex plants inoculated with K599 carrying CRISPR/Cas9-*GmSHMT05*-T3+T5. **C**. GFP (+) roots of Essex plants inoculated with K599 carrying CRISPR/Cas9-*GmSHMT08*-T1+T4. **D**. GFP (+) roots of Essex plants inoculated with K599 carrying CRISPR/Cas9-*GmSHMT05&08*-T1+T2. Roots selected for DNA extraction are in red. **E**. Gel picture of amplified fragments from genomic DNA extracted from roots transformed with CRISPR/Cas9-*GmSHMT05*-T3+T5. Fragments flanking T3 and T5 gRNA cleavage sites were amplified using F1 and R1 primers. **F**. Gel picture of amplified fragments from genomic DNA extracted from roots transformed with CRISPR/Cas9-*GmSHMT08*-T1+T4. Fragments flanking T1 and T4 gRNA cleavage sites were amplified using F2 and R2 primers. **G**. Gel picture of amplified fragments of *GmSHMT05* from genomic DNA extracted from roots transformed with CRISPR/Cas9-*GmSHMT05&08*-T1+T2. Fragments flanking T1 and T2 gRNA cleavage sites were amplified using F1 and R1 primers. **H**. Gel picture of amplified fragments of *GmSHMT08* from genomic DNA extracted from roots transformed with CRISPR/Cas9-*GmSHMT05&08*-T1+T2. Fragments flanking T1 and T2 gRNA cleavage sites were amplified using F3 and R3 primers. Arrows indicate the gel bands extracted for direct sequencing. White arrows indicate bands with WT fragments. Red arrows indicate bands with edited sequences. **I**. Sequences of selected bands in G and H. The editing of genes were confirmed. WT indicates non edited sequences. Chimeric indicates the mixture of WT and edited sequences. If WT is dominant in chmeric sequences, it is denoted as chimeric (WT dominant). The **bolded** bases indicate multiple peaks at that position, but the dominant base matches the WT based on the sequence. The blue colored "**N**" denotes chimeric peaks where bases differ from WT, representing a mixture of WT bases and newly introduced bases across multiple amplified alleles. PAM sequence is in red color. L = 100 bp ladder. WT = Wild Type. NT = No Template

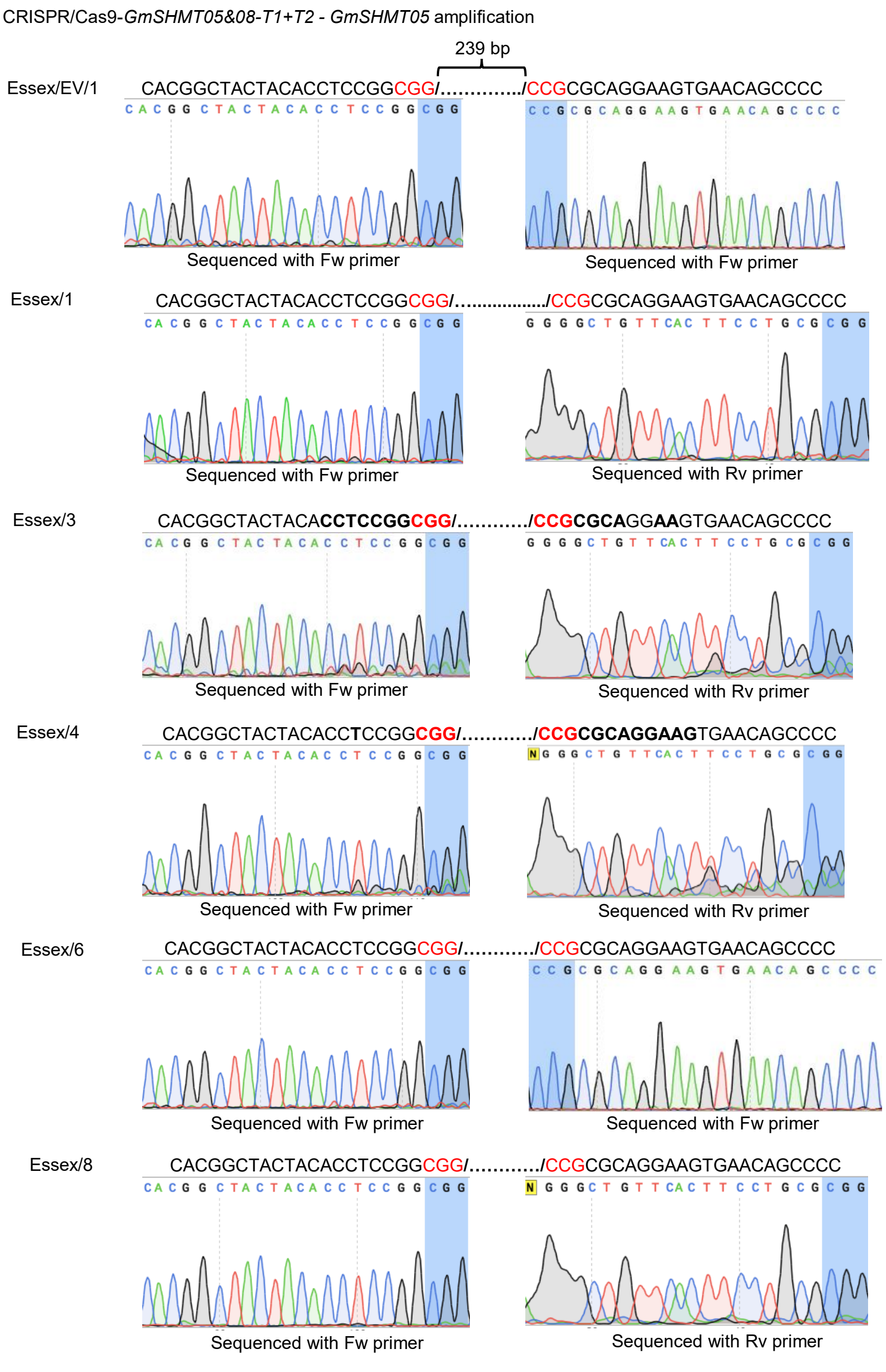

Supplementary Figure S18. Amplicon sequencing to confirm the editing of *GmSHMT05* in Essex targeted by CRISPR/Cas9-*GmSHMT05&08*-T1+T2 in replicate 3. Genomic DNA was extracted from transgenic hairy roots and the targeted fragments were amplified by PCR. Direct sequencing of PCR products confirmed successful gene edits, as indicated by the presence of deletions/insertions and re-annealing at regions targeted by sgRNA. The **bolded** bases indicate multiple peaks at that position, but the dominant base matches the WT bases on the sequence. The blue colored "**N**" denotes chimeric peaks where bases differ from WT, representing a mixture of WT bases and newly introduced bases across multiple amplified alleles. PAM sequence is in red color

CRISPR/Cas9-*GmSHMT05&08-T1+T2* - *GmSHMT08* amplification

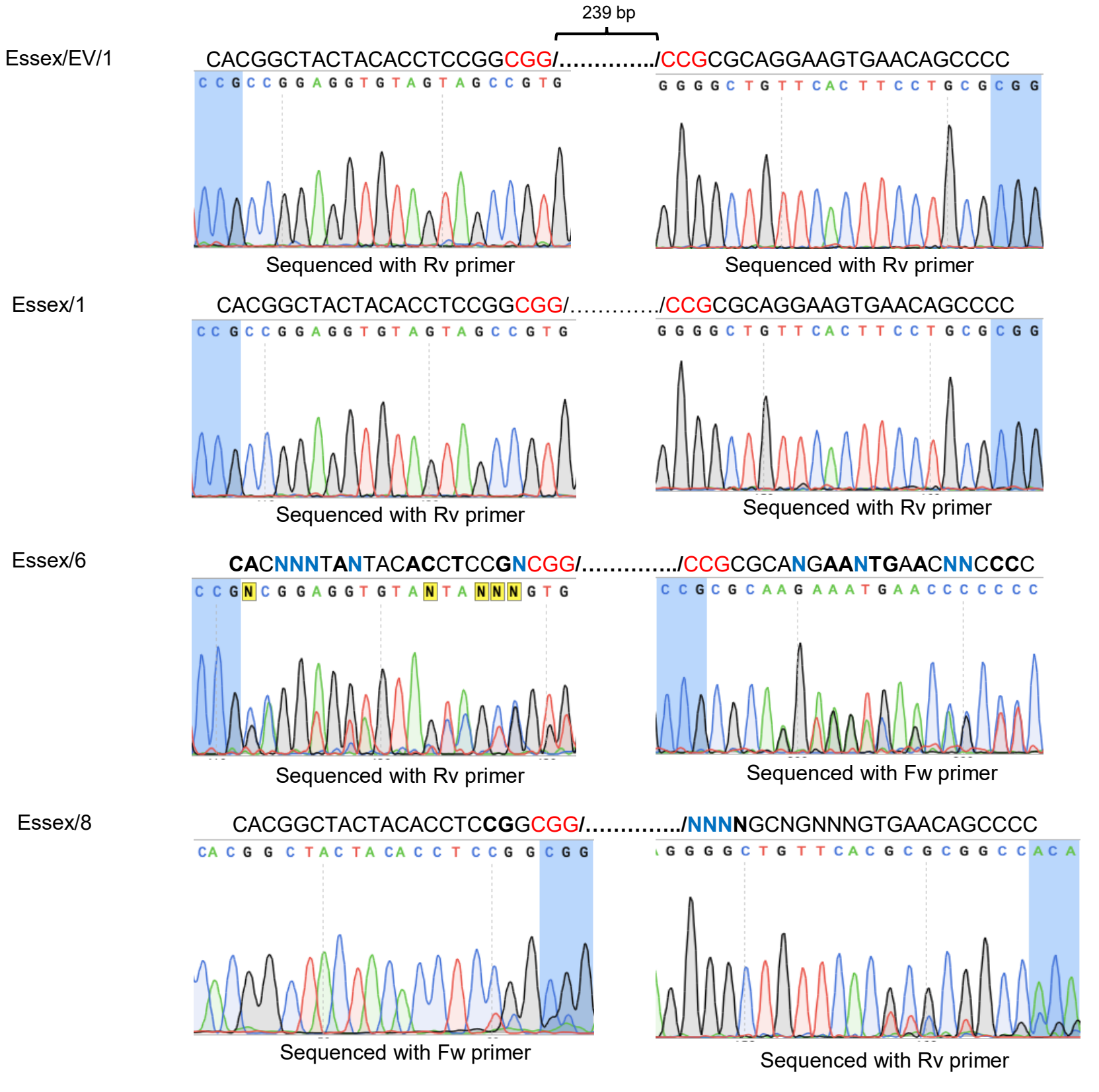

Supplementary Figure S19. Amplicon sequencing to confirm the editing of *GmSHMT08* in Essex by CRISPR/Cas9-*GmSHMT05&08*-T1+ in replicate 3. Genomic DNA was extracted from transgenic hairy roots and the targeted fragments were amplified by PCR. Direct sequencing of PCR products confirmed successful gene edits, as indicated by the presence of deletions/insertions and re-annealing at regions targeted by sgRNA. The **bolded** bases indicate multiple peaks at that position, but the dominant base matches the WT bases on the sequence. The blue colored "**N**" denotes chimeric peaks where bases differ from WT, representing a mixture of WT bases and newly introduced bases across multiple amplified alleles. PAM sequence is in red color.

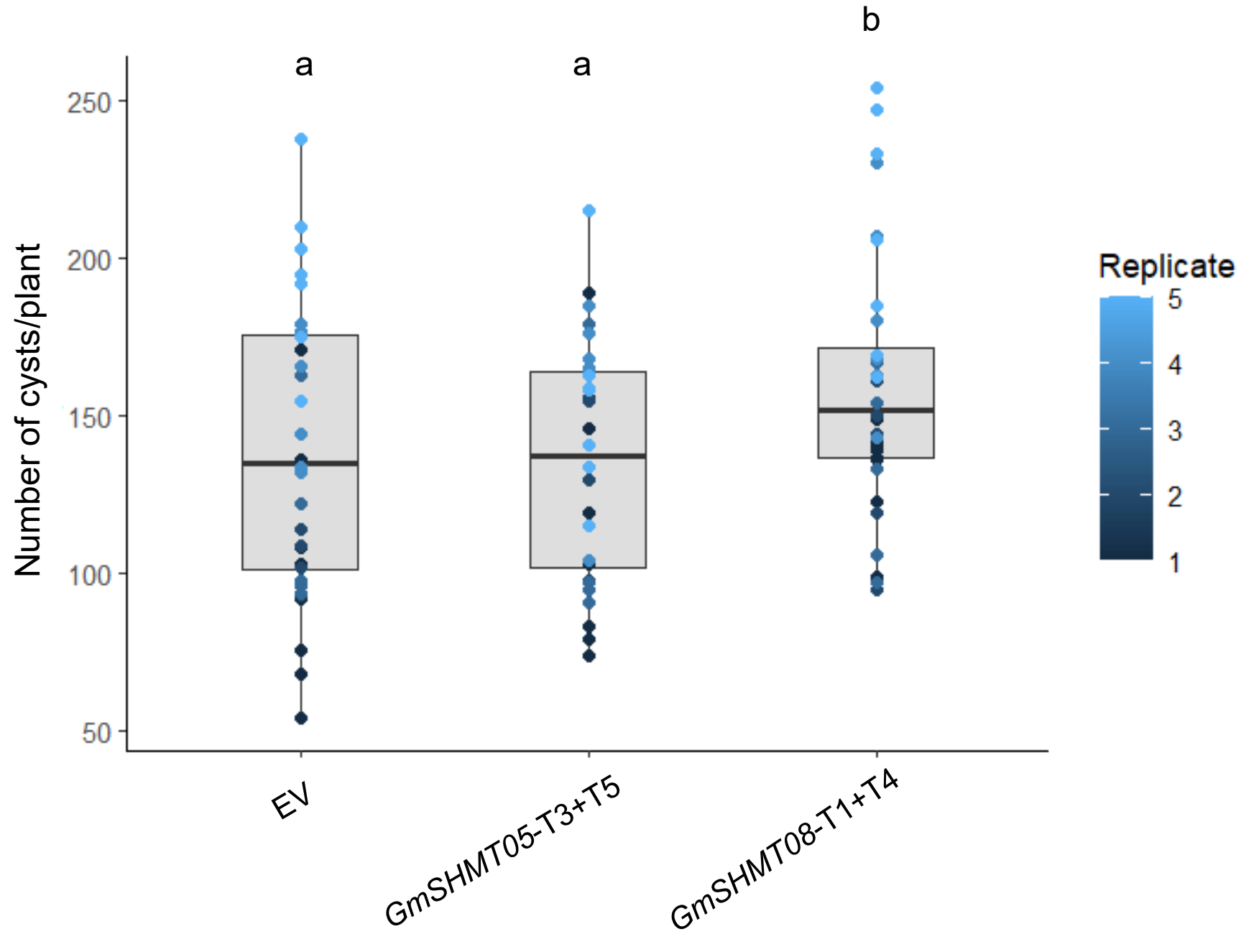

Supplementary Figure S20. SCN phenotyping in Essex inoculated K599 carrying EV, CRISPR/Cas9-*GmSHMT05*-T3+T5, or CRISPR/Cas9-*GmSHMT08*-T1+T4. Essex with CRISPR/Cas9-*GmSHMT08*-T1+T4 (or knockout line) displays significantly higher cyst counts compared to the Essex EV and Essex/*GmSHMT05*-T3+T5 lines, indicating an increase in basal susceptibility. Data represent means ± s.d. Different letters above bars indicate significant differences at $p < 0.05$ according to ANOVA followed by Fisher's Least Significant Difference (LSD) test.
